# Dihydrogen contacts observed by through-space indirect NMR coupling†
†Electronic supplementary information (ESI) available: Experimental procedures, spectroscopic data of all new compounds, and NMR and calculation details. See DOI: 10.1039/c8sc02859a


**DOI:** 10.1039/c8sc02859a

**Published:** 2018-08-13

**Authors:** Martin Dračínský, Michal Buchta, Miloš Buděšínský, Jana Vacek-Chocholoušová, Irena G. Stará, Ivo Starý, Olga L. Malkina

**Affiliations:** a Institute of Organic Chemistry and Biochemistry , Czech Academy of Sciences , Flemingovo nám. 2 , 166 10 Prague 6 , Czech Republic . Email: dracinsky@uochb.cas.cz; b Institute of Inorganic Chemistry , Slovak Academy of Sciences , Dúbravská cesta 9 , SK-84536 Bratislava , Slovakia . Email: Olga.Malkin@savba.sk

## Abstract

“Through-space” indirect spin–spin couplings between hydrogen atoms formally separated by up to 18 covalent bonds have been detected by NMR experiments in model helical molecules. It is demonstrated that this coupling can provide crucial structural information on the molecular conformation in solution. The coupling pathways have been visualised and analysed by computational methods. The conformational dependence of the coupling is explained in terms of orbital interactions.

## Abstract 2

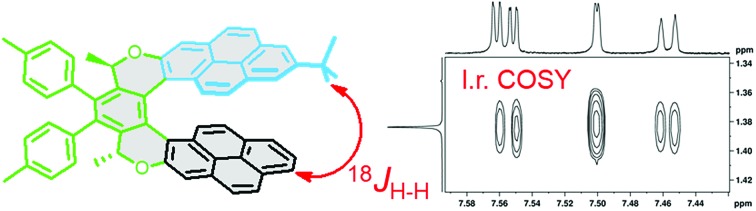
Through-space NMR indirect couplings between hydrogen atoms formally separated by 18 covalent bonds have been detected. The coupling pathway has been visualised and analysed by computational methods.

## Introduction

Unambiguous determination of a molecular structure by X-ray analysis has been central to many achievements in chemistry and biology.^1^ The method provides the exact atomic spatial coordinates, under a specific set of conditions where molecules are frozen in place in the crystal lattice of a solid. However, most of the interesting behaviour of molecules takes place in solution, raising the question of whether, and how much, the molecular structures in crystal and solution really resemble each other. This can be an issue for molecules exhibiting significant conformational freedom, as solvation and crystal packing may affect their structure.^2^,^3^

**Fig. 1:**
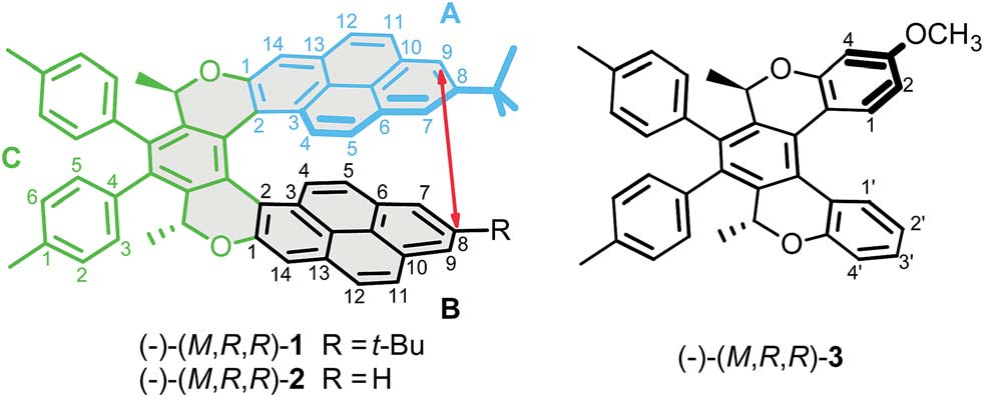
The structures of the *C*_2_-symmetric pyrene oxa[7]helicene (–)-(*M*,*R*,*R*)-**1**, its asymmetrical analogue (–)-(*M*,*R*,*R*)-**2**, and the smaller model compound (–)-(*M*,*R*,*R*)-**3**. The pyrene subunits **A** (blue) and **B** (black) in **2** are shown in different colours, as is the central part **C** (green). The helix pitch of the molecule is indicated by a red arrow, and the helical backbone is highlighted in grey.

Helicenes and their analogues^4^–^15^ illustrate this problem well. Their helix pitch is a parameter well-documented in the crystalline state (see the Cambridge Structural Database)^16^ but it has been shown experimentally that this structural parameter may be substantially different in solution.^17^ Moreover, a striking difference has been found between the compressed single-crystal structure of the *C*_2_-symmetric pyrene oxa[7]helicene (–)-(*M*,*R*,*R*)-**1** (Fig. 1) and the more elongated structure obtained by DFT calculation.^18^ In this context, knowledge of the relationship between the single-crystal, solution, and calculated structures would be important for a proper understanding of the physico-chemical properties involved. For instance, recent theoretical studies showed that the helix pitch of helicenes, which might, in principle, be tuned by applying an external mechanical force, dramatically influences the single molecule conductivity,^19^,^20^ piezoelectric properties,^21^ and thermopower efficiency.^22^

An estimate of internuclear distances for molecules in solution may be provided by NMR experiments employing the homonuclear proton–proton dipole–dipole cross-relaxation effect, known as the nuclear Overhauser effect (NOE). However, the interacting spins are rarely isolated from interaction with other spins, and the resulting spin diffusion complicates quantitative interpretation of the NOE-type experiments.^23^ Indirect spin–spin (scalar) *J*-coupling of nuclear magnetic momenta may provide a promising alternative for determination of atomic positions and interactions in molecules in solution. Because this interaction is mediated by bonding electrons, its presence is normally considered proof of a covalent link between two atoms, and implicitly reflects the bond strength as well. However, measurements of scalar couplings between nuclei not connected by conventional chemical bonds^24^–^33^ have been made for a variety of systems. Such through-space coupling (TSC)^34^ can be inferred, for example, where an unexpectedly large coupling arises between two atoms close in space but separated by several bonds.^32^ Indirect coupling transmitted through hydrogen bonds is well documented in biomolecular structure determination.^35^–^38^ TSC has usually been observed between atoms with overlapping electron lone pairs, particularly between two fluorines;^39^–^42^ but it has also been detected for fluorine–carbon, fluorine–nitrogen,^43^,^44^ fluorine–hydrogen,^39^,^45^ phosphorus–phosphorus,^46^,^47^ and carbon–phosphorus^48^,^49^ interactions, as well as – more rarely – between metallic nuclei.^50^ Interestingly, TSC has also been detected between hydrogen nuclei formally separated by seven covalent bonds in *para*-cyclophanes, and the magnitude of the coupling was found to be strongly distance- and conformation-dependent.^51^ Spin–spin coupling of 2.0 Hz has been detected very recently between two exceptionally close non-bonded hydrogen atoms in a substituted triptycene.^52^

In this paper, we report on the “through-space” scalar coupling (TSC) between hydrogen nuclei, which may be used for accurate determination of molecular conformation. This coupling has been detected in the model compound (–)-(*M*,*R*,*R*)-**3** and in the unsymmetrical pyrene dioxa[7]helicene (–)-(*M*,*R*,*R*)-**2** (Fig. 1) by a long-range COSY experiment. In compound (–)-(*M*,*R*,*R*)-**2**, the coupling is observed between hydrogen nuclei that are formally separated by 18 covalent bonds. The TSC has been analysed in terms of the coupling pathways, and the consequences for our understanding of chemical bonding are discussed.

## Results and discussion

Long-range COSY experiments were used to detect small *J*-coupling interactions, including those transmitted through-space, in the studied molecules. A two-dimensional *J*-resolved experiment was used to measure the magnitudes of small couplings that were not observable as line splitting in 1D ^1^H NMR spectrum. The *J*-resolved experiment separates chemical shifts (observed in one dimension) and *J*-couplings (observed in the second dimension) and allows for the detection of coupling patterns with much better resolution than conventional 1D spectra. Full description of the experimental methods and of the synthesis of compound (–)-(*M*,*R*,*R*)-**2** is given in the ESI.†

### NMR spectroscopy of compound (–)-(*M*,*R*,*R*)-**3**

To demonstrate the possibility of detecting through-space coupling between hydrogen atoms in helical molecules, we first turned our attention to compound (–)-(*M*,*R*,*R*)-**3**. In crystals of the related molecule 2,5-dimethyl-2,5-dihydrobenzo[3,4]isochromeno[6,5-*c*]chromene, which is (–)-(*M*,*R*,*R*)-**3** without methoxy and tolyl groups, the distance between H-1 and H-1′ was determined by X-ray crystallography to be 2.68 Å.^53^

The signals of hydrogen atoms H-1 and H-1′ are well separated from other signals in the ^1^H NMR spectrum of compound (–)-(*M*,*R*,*R*)-**3**. The long-range COSY experiment unambiguously showed spin–spin interaction between these hydrogens (ESI, Fig. S2†). This interaction is formally through seven covalent bonds, but only the interaction between H-1 and H-1′, which are close in space, is apparent in the spectrum; no other interaction through six or more bonds could be detected. Judging from the cross-peak intensities, the TSC is larger than the ^5^*J* couplings H1–H4 and H1′–H4′. However, the TSC is still too small to cause observable line splitting in the 1D proton spectrum.

**Fig. 2:**
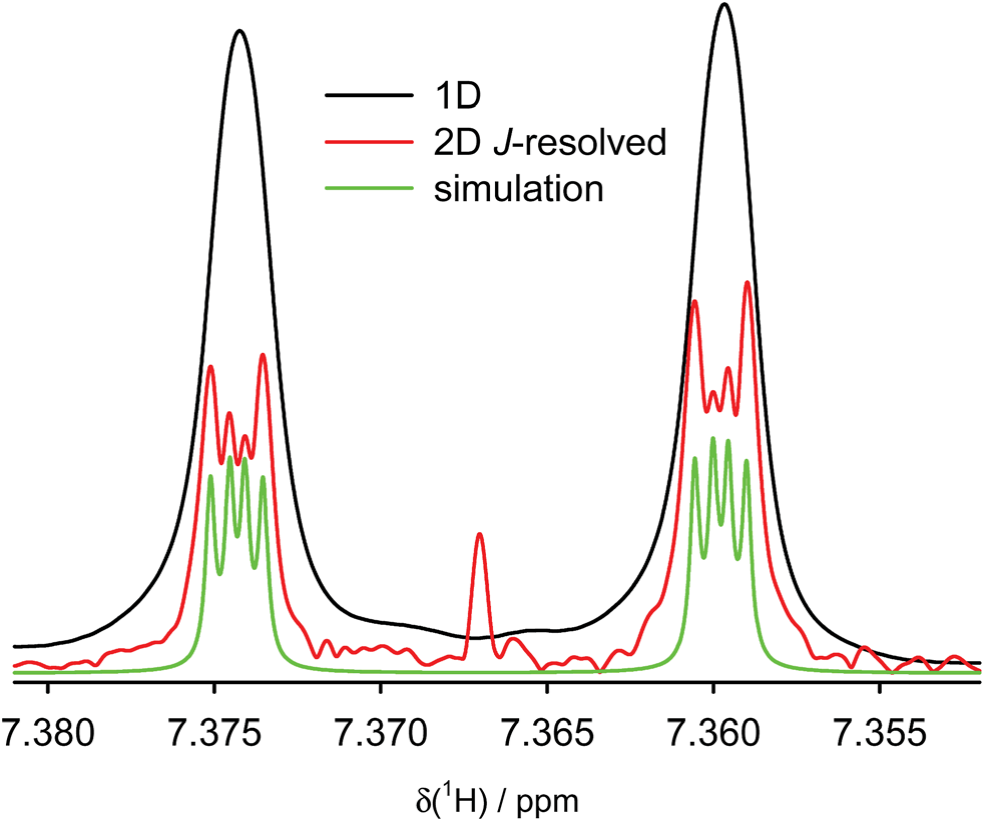
Experimental NMR signal of hydrogen H-1 of compound (–)-(*M*,*R*,*R*)-**3** in conventional 1D ^1^H NMR spectrum (black), extracted from 2D homonuclear *J*-resolved experiment (red) and simulated with the experimental *J*-coupling values listed in Table 1 and line broadening of 0.2 Hz (green).

In order to determine the magnitude of the H1–H1′ coupling, we carried out a two-dimensional homonuclear *J*-resolved experiment. Fig. 2 shows the trace of the signal of hydrogen H1 in the *J*-resolved experiment; the full spectrum is shown in the ESI (Fig. S3 and S4†). It is clear that the signal resolution is significantly improved by this technique and that very small couplings can be detected and quantified. Comparison of the line splitting of H1 with that of other signals allowed determination of the magnitude of the TSC as ^7^*J*_H1,H1′_ = 0.61 Hz, while that of the conventional through-five-bonds coupling between H1 and H4 was found to be 0.33 Hz (Table 1).

**Table 1:** DFT-calculated (B3LYP) and experimental *J*-couplings (Hz) in compound (–)-(*M*,*R*,*R*)-**3**, with calculated FC contributions to the total through-space 1–1′ coupling

Coupling	Model-A	Model-B	Experiment
TSC 1–1′	0.62	0.62	0.61
FC 1–1′	0.56	0.56	
1–2	8.48	8.59	8.73
1–4	0.36	0.43	0.33
1′–2′	7.64	7.79	7.88
1′–3′	1.42	1.20	1.66
1′–4′	0.45	0.62	0.51

### Computations – compound (–)-(*M*,*R*,*R*)-**3**

To verify the experimental findings and to provide additional insight into the through-space coupling, we performed a detailed computational analysis of the phenomenon. We mostly relied on DFT calculations; however, to confirm that the calculated TSCs are not artefacts of the selected DFT computational protocol, we performed coupled cluster singles and doubles (CCSD) calculations for a small model of the studied compounds. CCSD is highly computationally demanding but provides a very accurate description of molecules with electron correlation. Detailed description of the computational methods is given in the ESI.†

As discussed before,^54^ reliable calculation of an indirect coupling requires a large atomic orbital basis set. It has been found that adding polarisation or diffuse functions on heavy atoms, and going from a double- to a triple-zeta basis, have a particularly dramatic effect on computed through-space couplings. The use of a small basis set typically leads to an overestimation of the TSCs.^55^ It has been found that the 6-311++G(2df,2pd) or IGLO-III basis set with the B3LYP functional provides coupling constants in good agreement with experiment.^51^ Adding a polarisable continuum model to simulate solvation does not significantly affect the calculated couplings. The remaining errors in the TSC calculation are attributed to the inaccuracy of the B3LYP method and the lack of anharmonic and dynamical averaging.^56^–^58^

**Fig. 3:**
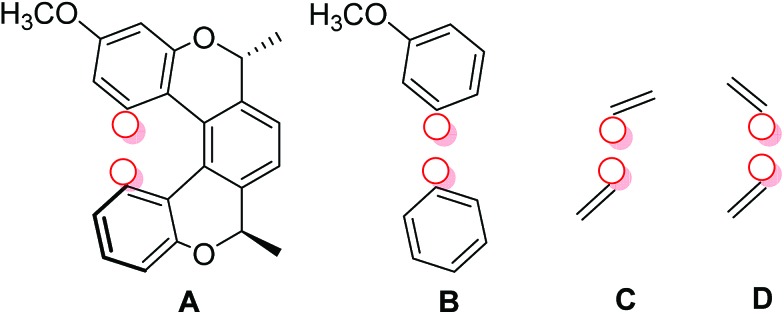
The structure of truncated models of compound (–)-(*M*,*R*,*R*)-**3** used for the calculation of TSC. The position of the coupled hydrogen atoms is shown by red circles.

We calculated the *J*-couplings in molecule (–)-(*M*,*R*,*R*)-**3** using the B3LYP functional and IGLO-III basis set. However, as the IGLO-III set is rather computationally demanding, several truncated molecular models were used for the calculation; four of them are shown in Fig. 3 and the remaining (E–G) in the ESI.† Model A contains the pentahelicene unit with the tolyl groups exchanged for hydrogen atoms; the positions of all atoms were optimised at the B3LYP/6-31g(d,p)/GD3 level before the NMR calculations. In model B, only two terminal benzene segments were left from the original molecule. The most simplified models C and D consist only of two ethylene molecules. The position of the individual units in the truncated models was fixed in the same place as in model A, and only the positions of the hydrogen atoms added to all the dangling bonds were optimised.

The TSC and through-bond couplings calculated for model A agree very well with the experimental values (Table 1). More importantly, the magnitude of the TSC is almost the same in all the truncated models (Tables 1 and 2 and ESI†), which further confirms the through-space character of the coupling, because the coupled nuclei are not connected by covalent bonds in models B–G. Even for the very small models, the agreement of the calculated TSC with experiment is very good.

**Table 2:** DFT- and CCSD-calculated through-space *J*-couplings (Hz) and FC contributions to the total *J*-coupling in the smallest models of compound (–)-(*M*,*R*,*R*)-**3** (depicted in Fig. 3). All calculations were performed with the IGLO-III basis set

Coupling	Method	Model-C	Model-D	Experiment
TSC 1–1′	DFT(B3LYP)	0.66	0.68	0.61
FC 1–1′	DFT(B3LYP)	0.61	0.68	
FC 1–1′	DFT(PP86)	0.51	0.54	
FC 1–1′	CCSD	0.46	0.50	

According to the classic theory of Ramsey,^59^ four different terms contribute to the indirect spin–spin coupling: the Fermi contact (FC, usually the most important), spin–dipole (SD), paramagnetic spin–orbit (PSO), and diamagnetic spin–orbit (DSO) terms. The Fermi contact dominates the contributions to the total TSC calculated for models A–G (FC always forms more than 90% of the total TSC).

To confirm that the calculated TSC is not an artefact of the DFT computational level, we performed calculations of the Fermi contact contribution using another exchange-correlation functional at the DFT level (PP86) and at the coupled cluster CCSD level with the same basis set (IGLO-III) and geometry as used in the DFT calculations. The CCSD-calculated FCs (Table 2) are only *ca.* 25% smaller than those calculated at the DFT(B3LYP) level; the DFT(PP86) results are even closer. These calculations clearly indicate that TSC between hydrogen atoms not connected by covalent bonds is not a computational artefact but a real phenomenon that can be analysed by DFT calculations.

We also investigated the basis set dependence of the leading contribution to TSC arising from the Fermi contact interaction at the DFT and CCSD level (see details in ESI†). These calculations demonstrated that the IGLO-III basis set provides well-converged results.

### NMR spectroscopy of (–)-(*M*,*R*,*R*)-**2**

Encouraged by the detection of TSC in compound (–)-(*M*,*R*,*R*)-**3**, we turned our attention to the unsymmetrical pyrene dioxa[7]helicene (–)-(*M*,*R*,*R*)-**2**, which is a close analogue of the intriguing (–)-(*M*,*R*,*R*)-**1**,^18^ and employed NMR spectroscopy to detect through-space indirect coupling, which we believed might be used to obtain information about internuclear distances.

Through-space indirect *J*-coupling of nuclear magnetic momenta may provide a promising alternative for determination of hydrogen atom positions and interactions in molecules in solution. Accordingly, we decided to study the helix pitch in (–)-(*M*,*R*,*R*)-**2** by employing a long-range COSY experiment to observe the indirect coupling that should arise between hydrogen nuclei close in space.

**Fig. 4:**
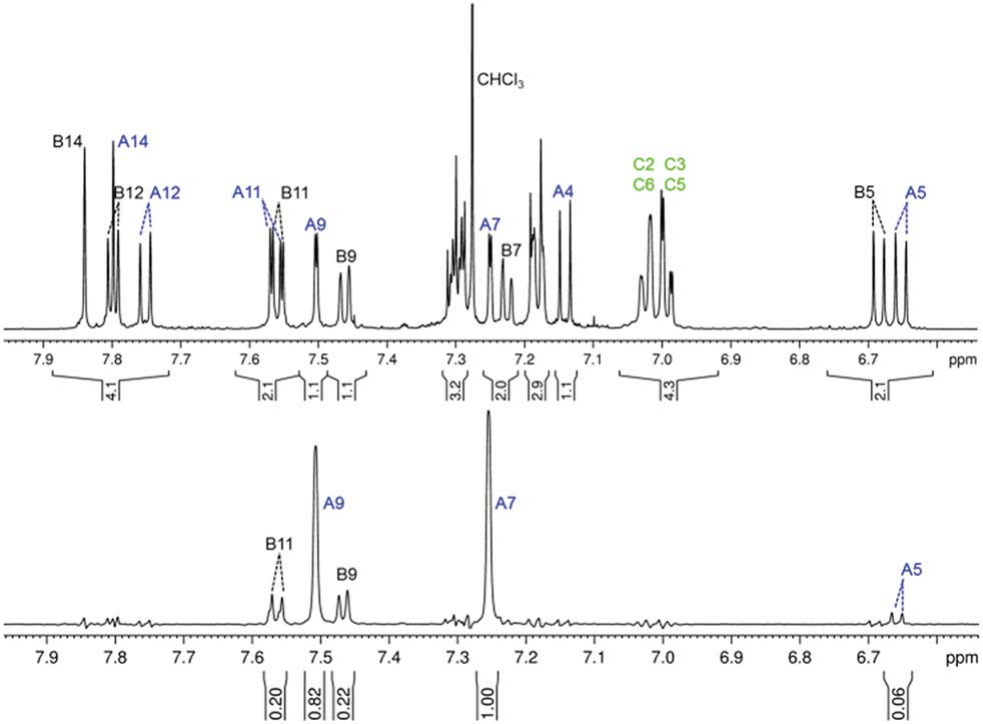
An aromatic region of the ^1^H NMR spectrum of (–)-(*M*,*R*,*R*)-**2** measured on a 600 MHz spectrometer in CDCl_3_ (top), and a selective differential 1D ROESY experiment with irradiation of the signal of the *tert*-butyl hydrogen nuclei (at 1.38 ppm).

An aromatic region in the ^1^H NMR spectrum of compound (–)-(*M*,*R*,*R*)-**2** is depicted in Fig. 4 (top). The signals in the spectrum are well separated, which allows for a complete signal assignment by a combination of one-dimensional ^1^H and ^13^C and 2D homo- and heteronuclear correlation experiments (H,H-COSY, H,C-HSQC, H,C-HMBC), see details in the ESI.† The structure of the molecule and the signal assignment was further confirmed by a selective 1D rotating frame Overhauser spectroscopy (ROESY) experiment with irradiation of the *tert*-butyl hydrogen nuclei at 1.38 ppm (Fig. 4, bottom), which confirmed that the *tert*-butyl group attached to part A of the compound (–)-(*M*,*R*,*R*)-**2** is close in space to part B of the molecule, particularly the hydrogen atoms B9 and B11.

**Fig. 5:**
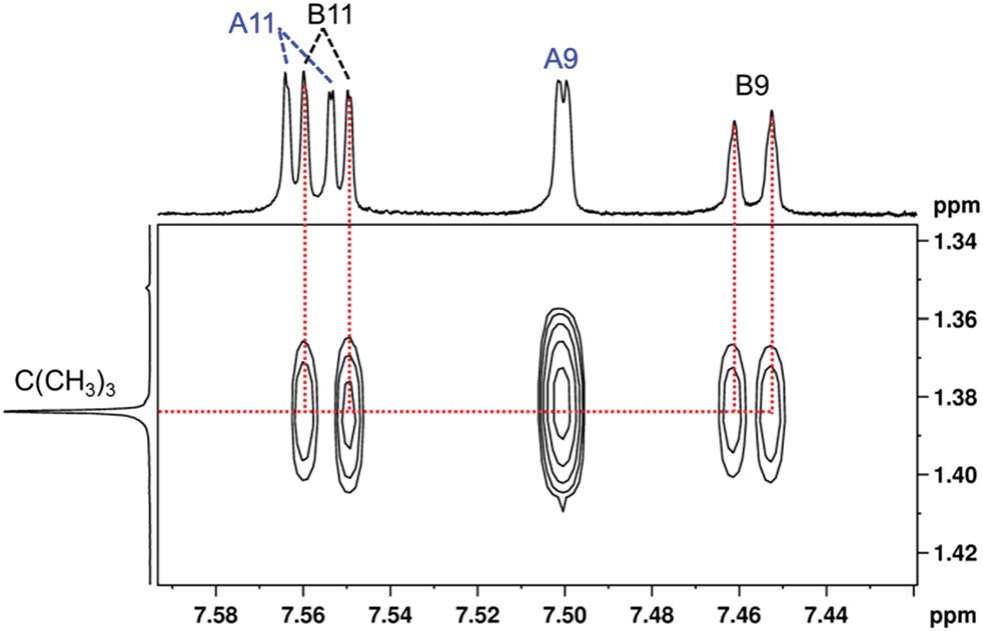
A part of the long-range COSY spectrum of (–)-(*M*,*R*,*R*)-**2** measured on an 850 MHz spectrometer in CDCl_3_. Full spectrum in the ESI (Fig. S8†).

The long-range COSY experiment confirmed the existence of through-space indirect coupling between the *tert*-butyl hydrogen atoms and aromatic protons from the opposite end of the molecule. Part of the long-range COSY spectrum, containing the interactions of the *tert*-butyl hydrogens, is depicted in Fig. 5. The strongest interactions observable in the spectrum are those involving hydrogen atoms A9 and A7 (A7 not shown in Fig. 5). These nuclei are separated by 5 covalent bonds from the *tert*-butyl hydrogens, and the observation of weak indirect coupling is, therefore, not surprising. On the other hand, the measurement of weaker cross-peaks corresponding to interactions with the hydrogen atoms B9 and B11 represents a hitherto-unprecedented observation of hydrogen–hydrogen interactions across (formally speaking) 18 covalent bonds. Importantly, it is only interactions with hydrogen atoms B9 and B11, which are close in space to the *tert*-butyl group (as confirmed by ROESY), that are observed; no other interaction with ring B hydrogens appears in the spectrum.

This through-space interaction is very weak and does not lead to an observable signal splitting in the 1D proton spectrum, and even the resolution of the 2D homonuclear *J*-resolved experiment is not sufficient to quantify the magnitude of the TSCs. The interaction results only in a subtle line broadening, as confirmed by selective homonuclear decoupling experiments (see Fig. S5 in ESI†); the spectral resolution is insufficient for precise determination of the coupling constants. The relative values of the coupling constants may be estimated from a 1D trace extracted from the 2D long-range COSY spectrum (shown in Fig. S7 in ESI†). Integration of the 1D trace allowed us to estimate that the through-space couplings of the *tert*-butyl group with hydrogens B9 and B11 are of the same magnitude, while the interaction with the A7 and A9 hydrogens is about three times larger.

### Computations – compound (–)-(*M*,*R*,*R*)-**2**

The molecular structure of (–)-(*M*,*R*,*R*)-**2** was optimised at the DFT level using the B3LYP functional and 6-31g(d) basis set. As van der Waals interactions between the two pyrene sections (A and B) of the molecule probably play an important role in determining the distance between them, we examined the effect of empirical dispersion corrections on the optimised geometry. Indeed, including the dispersion corrections in the calculations leads to much closer contacts between the pyrene sections (ESI†). The helix pitch of the molecule is characterised by the distance *d*, which is defined as the distance between the carbon atoms A9 and B8. Using the dispersion correction D2 (developed by Grimme)^60^ reduces *d* by almost half (7.0 Å → 3.8 Å), while the more advanced dispersion correction D3 (ref. 61) results in a slightly longer value (4.0 Å). The effect of solvation and functional choice is minor (Table S3 in the ESI†). Regardless of the method used for geometry optimisation, the *tert*-butyl group attached to part A of the molecule remains in close proximity to the hydrogen atoms B9 and B11, in agreement with the results of NOE and the long-range COSY experiment.

A one-dimensional potential for the ‘opening’ motion of the compound (–)-(*M*,*R*,*R*)-**2**, calculated as the energy of the optimised geometry for a particular fixed value of *d*, is shown in Fig. S12 in ESI.† The potential is relatively shallow (an increase of *d* by 1 Å leads to an increase of the potential energy by about 1.5 kcal mol^–1^). At room temperature, the Boltzmann thermal energy quantum *k*_B_*T* corresponds to *ca.* 0.6 kcal mol^–1^, so the distance *d* may fluctuate significantly around the equilibrium value.^62^ It is worth noting that such soft-spring behaviour of helicenes has also been described, or predicted, by other theoretical and experimental studies.^19^,^22^,^63^–^66^ Fast molecular dynamics may lead to the observation of an averaged TSC value.

For the calculation of *J*-couplings in (–)-(*M*,*R*,*R*)-**2**, three different molecular models were used: (i) the full molecule, (ii) a truncated model containing the two terminal pyrene sections, A and B, and (iii) the most simplified model consisting only of a *tert*-butylbenzene segment of part A and a naphthalene segment of part B. In the truncated models, the individual units were stacked over each other and fixed in positions reflecting the structure of the full molecule (hydrogen atoms were added to all the dangling bonds). The largest model (i, the full molecule) was used for geometry optimisation and *J*-coupling calculation with a smaller basis set (6-31g(d)); the two truncated models (ii and iii) were used to investigate the effect of the size of the basis set and to inspect the coupling pathway. We found that the size of the model had a very small effect on the calculated couplings (Table S4 in ESI†). Partial optimisation of the positions of added hydrogen atoms had no effect at all.

**Fig. 6:**
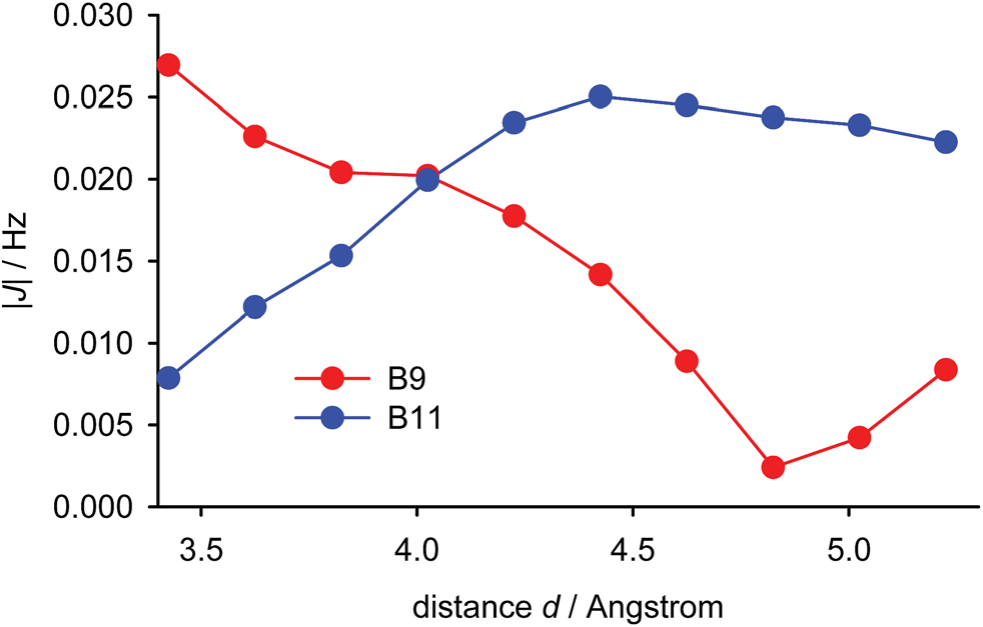
The dependence of the calculated absolute values of the indirect couplings between *tert*-butyl and B9 (red)/B11 (blue) hydrogen atoms in (–)-(*M*,*R*,*R*)-**2** (geometry model iii, B3LYP/IGLO-III) on the distance *d* between the carbon atoms A9 and B8. Note that the calculated coupling constants of the *tert*-butyl and B9 hydrogens are negative for distances 3.4–4.8 Å, but the sign of the experimental coupling constants is not determined.

The experimentally-observed couplings between the pyrene hydrogen atoms and those of the *tert*-butyl group reflect averaging of all the nine individual couplings of the *tert*-butyl hydrogen atoms. As shown in Fig. 6 for the most simplified model (iii, *vide supra*), the calculated dependence of the average of these through-space couplings on the distance *d* (utilising the IGLO-III basis set) differs significantly for the B9 and the B11 hydrogen atoms. The magnitudes of the two couplings are close only for *d* = 4 Å. Given the fact that the experimental couplings of the *tert*-butyl hydrogens with the B9 and B11 hydrogens are identical we can conclude that the distance *d* in (–)-(*M*,*R*,*R*)-**2** in solution is close to this value. Thus, the equilibrium geometry of (–)-(*M*,*R*,*R*)-**2** calculated with the empirical correction D3 reflects its actual structure. Furthermore, the magnitude of the aforementioned couplings for this distance is close to 0.02 Hz and the magnitudes of the five-bond couplings of the *tert*-butyl hydrogens to the A7 and A9 hydrogens are (in agreement with experiment) around three times larger (–0.061 and –0.075 Hz, respectively). The TSC distance dependence is close to linear around distances of 4.0 Å. Fast fluctuations of the distance *d* will therefore not change the magnitude of the coupling significantly.

The calculated distance dependence of the indirect couplings between the individual hydrogen atoms of the *tert*-butyl group and the hydrogen atoms B9 and B11 in (–)-(*M*,*R*,*R*)-**2** is shown in Fig. S13 in ESI.† Between 2 and 3 Å, the couplings increase almost linearly from –0.25 to 0.10 Hz, before dropping almost to zero for distances larger than 4 Å.

An analysis of the calculated values of the individual terms of the TSC for the *tert*-butylbenzene–naphthalene model iii shows that the FC, SD and DSO terms of the through-space couplings are well converged even with relatively small basis sets. In contrast, the PSO term requires large basis sets for convergence (see Table S5 in ESI†). The DSO and PSO terms have opposite signs and their sum is close to zero at convergence, leaving the FC term to dominate the through-space coupling (SD is small in all cases). With smaller basis sets, the PSO term does not fully converge, which leads to incomplete cancellation of DSO and overestimation of the total through-space coupling. Therefore, a reasonable estimate of through-space indirect couplings may be obtained from calculation of the FC term with a small basis set.

Similarly to compound (–)-(*M*,*R*,*R*)-**3**, we performed CCSD calculations of the Fermi contact term of the TSC for a very small model of compound (–)-(*M*,*R*,*R*)-**2** consisting of methane and ethylene molecules corresponding to fragments of the *tert*-butyl group and the pyrene of part B of the molecule. The DFT-calculated FC values agreed very well with the CCSD ones (see the ESI†).

The observation of the ‘through-space’ *J*-coupling between the hydrogen atoms under discussion calls for a new interpretation of the chemical bonding phenomenon. For indirect NMR spin–spin coupling two things are necessary: a pair of two magnetically-active nuclei, and the presence of electron density along some path connecting these two nuclei. The latter serves as a medium for transmitting the magnetic interaction. Experimental detection of a *J*-coupling confirms the presence of electron density between the coupled hydrogen atoms, which might be interpreted as one or more chemical bonds. However, the observation of *J*-coupling does not necessarily mean that the interaction between the coupled nuclei is attractive. There is an ongoing discussion in the literature as to whether close contacts between the congested hydrogen atoms in polycyclic hydrocarbons (*e.g*., phenanthrene) can be interpreted as chemical bonds or not;^67^ and in fact, the presence of typical values of ^1^H–^1^H coupling constants has been used as an argument against a bonding interaction.

### Visualisation of NMR spin–spin coupling pathways

Interpretation of such small NMR spin–spin couplings is very challenging. Given the through-space nature of *J*-couplings under study, it would be appropriate to visualise the pathways involved using real-space functions.^68^ The principal question is about the source of the electron density providing the coupling pathway; to answer this, we decided to plot the coupling deformation density (CDD), which shows the difference between the electron density distributions when the magnetic moments of the two interacting nuclei are parallel and antiparallel. From a physical point of view, CDD is an observable quantity (in theory), and reflects physical reality within the accuracy of the applied quantum–chemical method. It indicates which parts of the electronic structure are involved in the indirect spin–spin coupling between two particular nuclei, and nothing else. From the mathematical point of view, CDD is the bilinear response of the total electron density to the magnetic moments of the coupled nuclei. It can therefore be expressed as a linear combination of the products of pairs of unperturbed localized molecular orbitals (LMOs), *i.e.* the molecular orbitals of the system in the absence of nuclear magnetic moments (see ref. 69 for more details). The pairs can be formed by two occupied or two unoccupied orbitals, as well as cross terms between the occupied and unoccupied ones. A contribution to the CDD from a pair of LMOs shows the contribution of that pair to the changes in the total electron density occurring due to the magnetic interaction of the coupled nuclei. Contributions from individual pairs of molecular orbitals can be plotted separately or in groups. For all the plots shown below, the isosurface values were chosen such that all plotted surfaces would cover approximately the same volume.

**Fig. 7:**
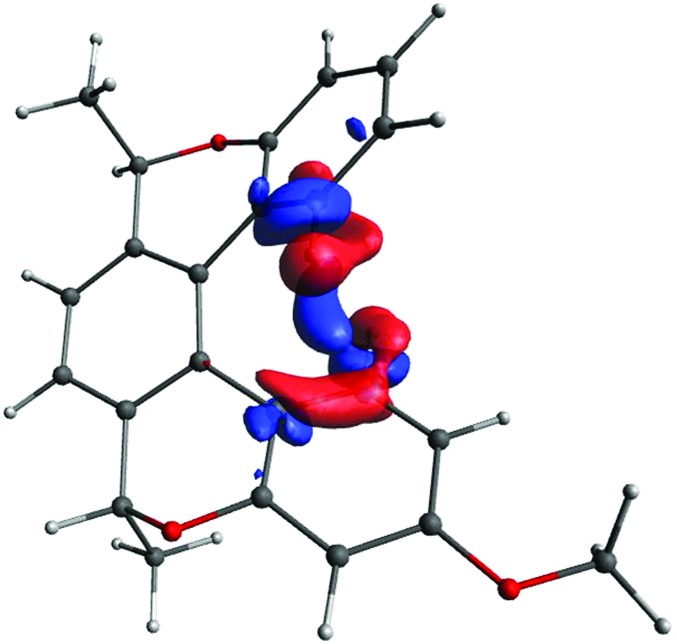
The coupling pathway for *J*(1–1′) in compound (–)-(*M*,*R*,*R*)-**3**. The isosurface value is 0.00008 a.u.

We start with visualisation of the spin–spin coupling pathway for compound (–)-(*M*,*R*,*R*)-**3** (see Fig. 7). The plot shows that it is indeed a through-space coupling rather than spin–spin coupling through seven bonds: the interaction between protons 1 and 1′ occurs not along the bonds, but along a path through the empty space between the coupled protons.

**Fig. 8:**
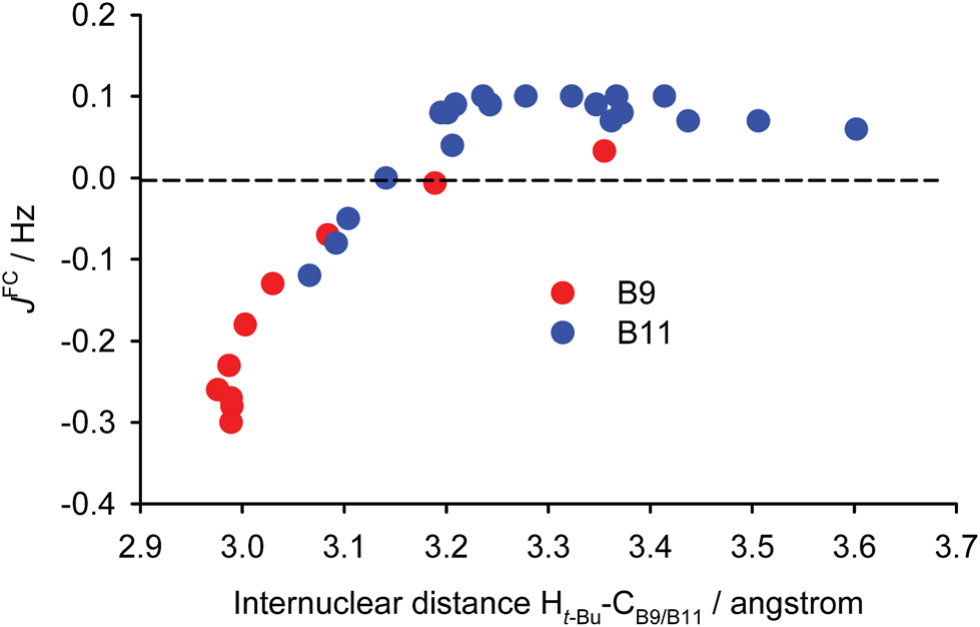
Dependence of the FC contribution to *J*(H_*t*-Bu_–H_B9/B11_) on the distance between the *tert*-butyl hydrogen and the B9/B11 carbon. The plot shows the results for the *tert*-butyl hydrogens with *R*(H_*t*-Bu_–C_B9/B11_) < 3.7 Å in model iii (B3LYP).

Before applying the visualisation tools to TSC spin–spin couplings in compound (–)-(*M*,*R*,*R*)-**2**, let us look again at the dependence on *d* of the calculated proton–proton couplings for the small model iii (see Fig. S13 in ESI†). For short and long distances the curves for B9 and B11 are close to each other. However, at a distance of about 4 Å, the curve for B9 is not uniquely defined and the values for B9 and B11 have opposite signs. This indicates that *d* is probably not the most relevant parameter. Therefore, we have chosen another variable – the distance between the *tert*-butyl (*t*-Bu) proton and the B9/B11 carbon bound to the second coupled proton (H_B9_ or H_B11_). Furthermore, we decided to concentrate only on the Fermi-contact term. This results in the data for the two protons, B9 and B11, lying practically on the same curve (Fig. 8). The *J*-couplings exhibit an almost linear dependence over shorter distances, starting with negative values, crossing the zero line at about 3.15 Å, and peaking at about 0.1 Hz. They then remain more or less constant for distances up to 3.7 Å before gradually decreasing for longer distances (not shown in the plot). To double-check this dependence, we performed a series of calculations with a different exchange-correlation potential (PP86) and using a different computational protocol, finite perturbation theory, instead of response theory (see Fig. S15 in ESI†). Since the admixture of Hartree–Fock exchange into the B3LYP functional gives more diffuse MOs than using pure DFT, the range of the PP86 results is somewhat smaller; and the numerical aspects of the finite perturbation theory likely lead to more numerical fluctuations. Nevertheless, the similarity between these two graphs is remarkable and indicates that the dependence under examination is not some artefact arising from a quirk in the theoretical calculations. This further indicates that the PP86 exchange-correlation potential can be used for visualisation.

**Fig. 9:**
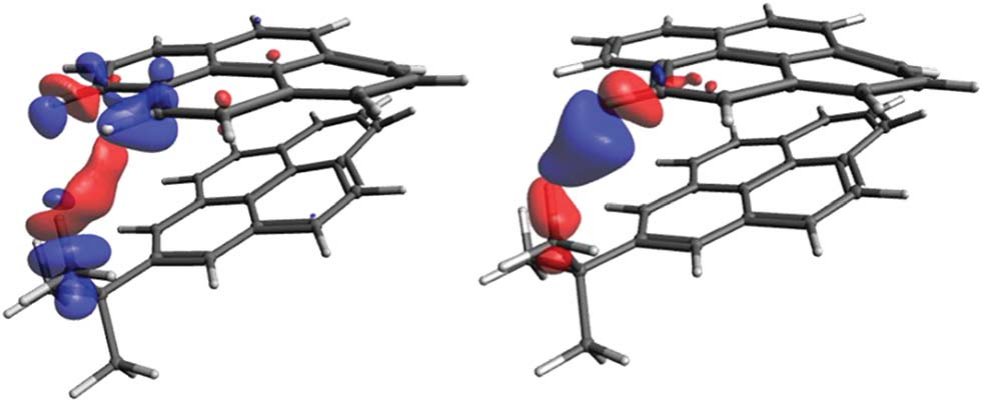
The coupling pathway for *J*(H_*t*-Bu_–H_B9_) (left) and for *J*(H_*t*-Bu_–H_B11_) (right). The isosurface values are 0.00007 a.u. and 0.0003 a.u, respectively.

The graph in Fig. 8 suggests that there are probably two competing pathways, one being stronger at shorter distances and leading to negative *J*, while the second one is probably dominant at moderate distances and leads to positive *J*. An obvious goal for further analysis is to try to disentangle these pathways. To do this, we will compare the coupling pathways for the B9 and B11 protons (see Fig. 9) in order to identify their common features and differences.

The coupling pathways for the B9 and B11 protons look very different. For B11, the pathway between the coupled protons contains two red lobes (negative) near the protons and a blue one (positive) in the middle. The pathway for proton B9 is more complicated. The CDD in the proximity of the coupled protons is positive (blue) and the curved lobe in the middle is red. In contrast to *J*(H_*t*-Bu_–H_B11_), the coupling pathway H_*t*-Bu_–H_B9_ also involves the electron density at and around an adjacent hydrogen (B11). In order to understand what makes the coupling pathways for protons B9 and B11 so different, a more detailed analysis involving localized molecular orbitals is needed. Fig. S16 in ESI† shows the coupling pathways for the model iii. The pathways in this model have similar topologies to those of the larger model shown in Fig. 9, justifying the use of the smaller model for more detailed analysis.

As mentioned above, the presence of electron density in the space between the interacting nuclei is a necessary condition for indirect spin–spin coupling. Usually this density is provided by the overlap of occupied molecular orbitals representing chemical bonds and/or lone pairs, which are there regardless of the presence of magnetic nuclei. As a result the main contributions to CDD typically come from the overlap of occupied molecular orbitals^70^,^71^ – in other words, from the occupied–occupied block of the CDD matrix. However, in our case, there is practically no electron density in the space between fragments A and B in the unperturbed system, *i.e.* when the magnetic interaction between H_*t*-Bu_ and H_B9/11_ is switched off – yet an indirect nuclear spin–spin coupling is observed experimentally, and confirmed by quantum-chemical calculations. Separation of the LMO pairs into three main groups – occupied–occupied, vacant–vacant and occupied-vacant – reveals that the contribution from the occupied–occupied and vacant–vacant blocks to CDD in the “through-space” area between the A- and B-planes is minor (see Fig. S17 and S18 in ESI†).

In contrast, the occupied-vacant block (see Fig. S19 in ESI†) contributes significantly to the through-space part for both couplings (even though they look rather different when plotted). This means that the Fermi-contact interaction of the proton nuclear magnetic moments perturbs the ground state density by admixing vacant orbitals. These perturbed orbitals, being more diffuse, make the through-space coupling possible. In other words, in this case the medium for transmitting the magnetic interaction is mainly provided by the Fermi-contact interaction, rather than by a chemical bond between the interacting protons. The same is true for compound (–)-(*M*,*R*,*R*)-**3** (see Fig. S20–S22 in ESI† showing the contributions to CDD from the occupied–occupied, occupied-vacant and vacant–vacant blocks of the CDD matrix for (–)-(*M*,*R*,*R*)-**3**).

**Fig. 10:**
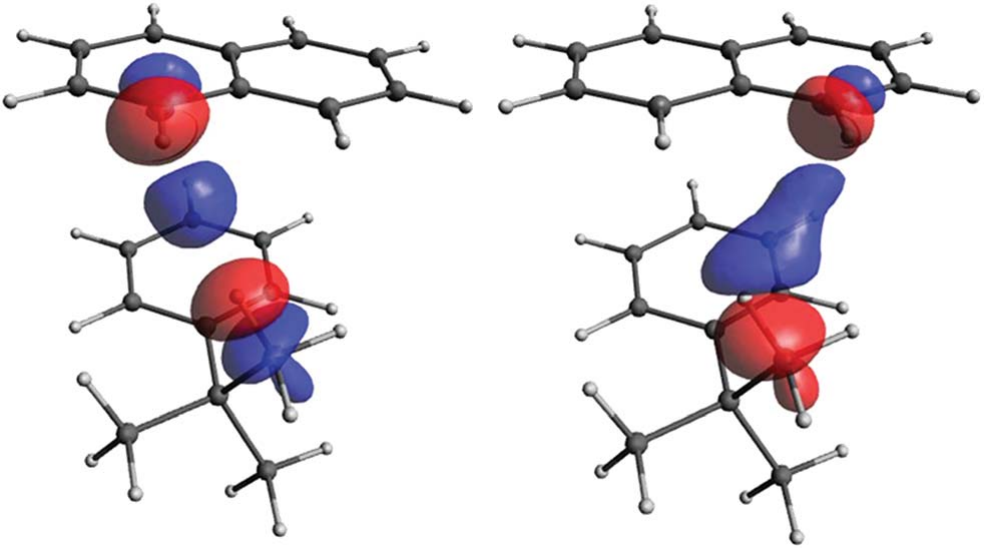
The primary pathway for the H_*t*-Bu_–H_B9_ (left) and H_*t*-Bu_–H_B11_ (right) couplings. The isosurface value is 0.00004 in both cases.

The occupied orbitals most affected by the perturbation are those closest to the coupled protons, and represent the C–H_*t*-Bu_ and C–H_B9/11_ bonds. The perturbation couples them primarily with their antibonding counterparts, σ*(C–H_*t*-Bu_) and σ*(C–H_B9/11_), which are also close to the coupled protons. These antibonding orbitals are more delocalised than the C–H bonds (see S23 in ESI). We decided to consider the total contribution to CDD from these four orbitals (that is, all their pairwise combinations, including occupied–occupied, vacant–vacant, and occupied–vacant) as the primary pathway (see Fig. 10). Of course, the primary pathway is not fully equivalent for the two couplings, due to the different positions of the C–H bonds involved. Nevertheless, it has some common features for the two protons. Topologically it resembles the pathway for the through-space coupling between atoms with lone pairs, for example two phosphorus atoms^69^ or two nitrogens.^71^ Since the through-space coupling, mediated *via* the overlap of lone pairs, is positive, one may reasonably expect the primary pathway to have a positive contribution to the value of the spin–spin coupling, as is typical for one-bond couplings between two nuclei with gyromagnetic ratios of the same sign.

**Fig. 11:**
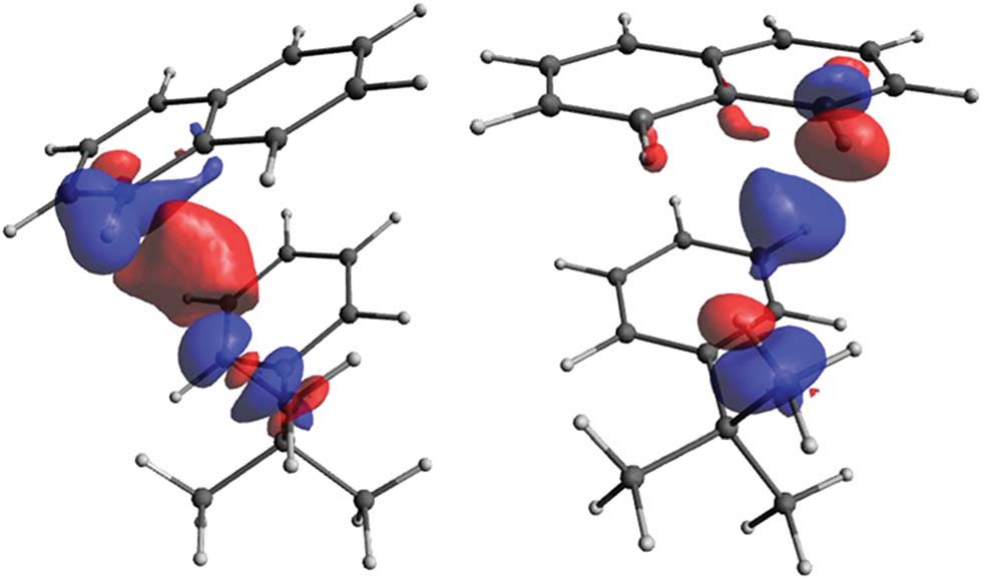
The secondary pathway for the H_*t*-Bu_–H_B9_ (left) and H_*t*-Bu_–H_B11_ (right) couplings. The isosurface values are 0.00004 and 0.00006, respectively.

However, although very important, the primary pathway constitutes only a part of the total interaction. The remainder, which we term the secondary pathway, provides a contribution comparable in magnitude (see Fig. 11).

In order to find the source of the electron density providing the secondary pathway, we have to look again at the pairs of occupied and vacant LMOs. In order for the pair to provide overlap density in the space between fragments A and B, one orbital should be associated with fragment A and another with B. We have calculated the overlap of densities for LMO pairs satisfying this condition for ten structures of model iii with different *d*, and selected the pairs with the largest overlap (see Fig. S24 in ESI†). The primary pathway is governed by the C–H_B9/11_–σ*(C–H_*t*-Bu_) and C–H_*t*-Bu_–σ*(C–H_B9/11_) overlaps. Among the orbitals contributing to the secondary pathway, the largest overlap is provided by σ*(C–H_*t*-Bu_) with the C_B9/11_–C single and double bonds. As seen from Fig. S24 in ESI,† at shorter distances the orbitals contributing to the secondary pathway have larger overlap, making this pathway dominant. Since the *J* values for shorter distances are negative, it is reasonable to assume that the secondary pathway gives a negative contribution. As the distance increases, the overlaps contributing to the secondary pathway diminish (on average) faster than those contributing to the primary pathway, and both pathways become near-equal in the magnitude of their effects. As a result, the *J* values for medium distances are close to zero. For long distances the primary pathway becomes dominant, which explains the positive sign of the couplings at those distances (a more detailed analysis of this is given in the ESI†).

An alternative explanation can be provided by using the Dirac vector model.^72^ The primary pathway directly connecting the H_*t*-Bu_ and H_B9/11_ may be considered an analogy to a one-bond coupling. The secondary pathway mainly involves the antibonding (C-H_*t*-Bu_) orbital and the C_B9/11_-C bonds and may be viewed as consisting of two parts: the through-space part between H_*t*-Bu_ and C_B9/11_, and a part through the C_B9/11_–H_B9/11_ bond that is analogous to a two-bond coupling. According to the Dirac vector model, if the nuclear spin of H_*t*-Bu_ is up, the probability of finding electrons with spin down will be higher near H_*t*-Bu_ due to the FC mechanism. Then, at the other end of a bond (or other electron cloud) connecting the perturbed nucleus with the next atom, the probability of alpha electrons (spin up) will be higher. For the primary pathway this means that spin-density near H_B9/11_ will be positive and, therefore, the spin of that nucleus will energetically prefer a downward orientation. This means that the contribution to *J*(H_*t*-Bu_–H_B9/11_) should be positive, as is typical for one-bond couplings. For the secondary pathway the “next” atom is C_B9/11_ and the spin-density here should be positive, too. It will also be positive at the “beginning” of the C_B9/11_–H_B9/11_ bond due to Hund's rule at C_B9/11_, and therefore negative at H_B9/11_. Consequently, from the point of view of the secondary pathway the energy will be lower with the H_B9/11_ nuclear spin up. The contribution from the secondary pathway to *J* should thus be negative, as is typical for two-bond couplings.

## Conclusions

A new type of through-space indirect spin–spin coupling between hydrogen nuclei has been detected in the helicene derivatives (–)-(*M*,*R*,*R*)-**2** and (–)-(*M*,*R*,*R*)-**3**. The existence of the coupling was unequivocally confirmed by long-range COSY experiment and its magnitude in the compound (–)-(*M*,*R*,*R*)-**3** was experimentally determined by a two-dimensional *J*-resolved experiment. DFT and CCSD calculations confirmed the existence of the through-space coupling phenomenon.

Coupling between the *tert*-butyl hydrogens and two hydrogen atoms (H_B9_ and H_B11_) from the opposite end of the molecule (–)-(*M*,*R*,*R*)-**2**, formally 18 covalent bonds away, was also observed by a long-range COSY experiment. The magnitude of this coupling is very small and could not be determined accurately; however, it could be estimated that the couplings between the *tert*-butyl hydrogens and both B9 and B11 hydrogens have the same magnitude.

Quantum-chemical calculations indicated that the sign and magnitude of the coupling in compound (–)-(*M*,*R*,*R*)-**2** is substantially distance-dependent. It was demonstrated that a combination of experimental and calculated coupling values may be used to determine the distance between fragments A and B, and hence, the conformation of the molecule. DFT calculations with an empirical correction for dispersion interactions yielded the same molecular conformation.

Analysis of the novel H–H TSC in LMO terms led us to separate the Fermi contact interaction into primary and secondary coupling pathways. The contributions from the primary and secondary pathways have opposite signs and different dependences on distance. The interplay between these two pathways may explain the dependence of the TSC value on molecular conformation. It also suggests that TSC between hydrogen atoms may be of similar use to conventional “through-bond” coupling when elucidating structures.

Furthermore, analysis of the TSC pathway indicates that the existence of the coupling cannot be interpreted as evidence of chemical bonding between the coupled hydrogen atoms.

## Conflicts of interest

There are no conflicts to declare.

## Supplementary Material

Supplementary information
