# Supplementary material for: Dihydrogen contacts observed by through-space indirect NMR coupling
Source: Chem Sci. 2018 Aug 13;9(38):7437–46. doi: 10.1039/c8sc02859a (PMC6180313; doi:10.1039/c8sc02859a)
Supplement: Supplementary file 1 [file SC-009-C8SC02859A-s001.pdf]

Electronic Supplementary Material (ESI) for Chemical Science.
This journal is © The Royal Society of Chemistry 2018

# Dihydrogen contacts observed by through-space indirect NMR coupling

Martin Dračínský,*[a] Michal Buchta,[a] Miloš Buděšínský,[a] Jana Vacek-Chocholoušová,[a] Irena G. Stará,[a] Ivo Starý[a] and Olga L. Malkina*[b]

[a]*Institute of Organic Chemistry and Biochemistry, Czech Academy of Sciences, Flemingovo nám. 2, 166 10 Prague 6, Czech Republic*

[b]*Institute of Inorganic Chemistry, Slovak Academy of Sciences, Dúbravská cesta 9, SK-84536 Bratislava, Slovakia*

Supporting Information

Table of contents

Synthesis…………………………………………………………… 2
NMR spectroscopy………………………………………….. 14
Computations………………………………………………… 20
Copies of NMR spectra…………………………………… 32
References………………………………………………………41

## Synthesis

**General:** Melting point was determined on Mikro-Heiztisch Polytherm A (Hund, Wetzlar) apparatus and is uncorrected. The $^{1}$H NMR spectra were measured at 400.13 MHz, 499.88 MHz, 600.13 MHz and 850.30 MHz, the $^{13}$C NMR spectra at 100.61 MHz, 125.71 MHz and 150.90 MHz in $CDCl_3$ or DMSO-$d_6$ as indicated in 5 mm PFG probe. For standardisation of $^{1}$H NMR spectra the internal signal of TMS (δ 0.0, $CDCl_3$) was used. In the case of $^{13}$C spectra, the residual signal of solvent (δ 77.00) was used. The chemical shifts are given in δ-scale, the coupling constants *J* are given in Hz. The IR spectra were measured in $CHCl_3$ or $CCl_4$ on FT-IR spectrometer Bruker Equinox 55. The EI mass spectra were determined at an ionising voltage of 70 eV, the *m/z* values are given along with their relative intensities (%). The standard 70 eV spectra were recorded in the positive ion mode. The sample was dissolved in chloroform, loaded into a quartz cup of the direct probe and inserted into the ion source. The source temperature was 220 °C. For exact mass measurement, the spectrum was internally calibrated using perfluorotri-*n*-butylamine (Heptacosa). The ESI mass spectra were recorded using ZQ micromass mass spectrometer (Waters) equipped with an ESCi multi-mode ion source and controlled by MassLynx software. Alternatively, the low resolution ESI mass spectra were recorded using a quadrupole orthogonal acceleration time-of-flight tandem mass spectrometer (Q-Tof micro, Waters) and high resolution ESI mass spectra using a hybrid FT mass spectrometer combining a linear ion trap MS and the Orbitrap mass analyzer (LTQ Orbitrap XL, Thermo Fisher Scientific). The conditions were optimised for suitable ionisation in the ESI Orbitrap source (sheat gas flow rate 35 a.u., aux gas flow rate 10 a.u. of nitrogen, source voltage 4.3 kV, capillary voltage 40 V, capillary temperature 275 °C, tube lens voltage 155 V). The samples were dissolved in methanol and applied by direct injection. 80% Methanol (flow rate 100 µl/min) was used as a mobile phase. The APCI mass spectra were recorded using an LTQ Orbitrap XL (Thermo Fisher Scientific) hybrid mass spectrometer equipped with an APCI ion source. The APCI vaporizer and heated capillary temperatures were set to 400 °C and 200 °C, respectively; the corona discharge current was 3.5 µA. Nitrogen served both as the sheath and auxiliary gas at flow rate 55 and 5 arbitary units, respectively. The ionisation conditions were the same for low-resolution as well as high-resolution experiment. The HR spectra were aquired at a resolution of 100 000. UV-Vis spectra were recorded on SPECORD 250 PLUS (Analytik Jena AG) with pure solvent (distilled THF) as a baseline. Optical rotations were measured in $CHCl_3$ using an Autopol IV instrument (Rudolph Research Analytical).

The commercially available catalysts and reagent grade materials were used as received. Diisopropylamine was distilled from calcium hydride under argon and degassed by three freeze-pump-thaw cycles before use; the THF, toluene and benzene were freshly distilled from sodium/benzophenone under nitrogen. TLC was performed on Silica gel 60 $F_{254}$-coated aluminium sheets (Merck) and spots were detected by the solution of $Ce(SO_4)_2$ . 4 $H_2O$ (1%) and $H_3P(Mo_3O_{10})_4$ (2%) in sulfuric acid (10%). The flash chromatography was performed on Silica gel 60 (0.040-0.063 mm, Fluka) or on Biotage® KP-C18-HS using the Isolera One HPFC system (Biotage, Inc.). Biotage Initiator EXP EU (300 W power) was used for reactions carried out in microwave oven. $Pd(PPh_3)_2Cl_2$ was purchased, CpCo(CO)(fum) was synthesised according to the literature procedure.[1] The starting materials (-)-(*S*)-**6**[2], **8**[3] and **15**[4] were synthesised according to the literature procedures.

Compound (-)-(*M*,*R*,R)-**3** was synthesised according to previously published procedure.[5]

**Synthesis of the model pyrene-based [7]helicene analogue (-)-(*M*,*R*,*R*)-2**

First, we carried out the preparation of the unsymmetrical pyrene-based dioxa[7]helicene analogue (-)-(*M*,*R*,*R*)-**2**, using the synthetic route developed earlier for its symmetrical congener (-)-(*M*,*R*,*R*)-**1**.[4] Starting from enantiopure pyrenyl alkyne (+)-(*R*)-**3** and pyrenyl iodide **4**, the $Pd^{II}/Cu^{I}$-catalysed Sonogashira coupling provided the bispyrenyl diyne (-)-(*R*)-**5** (Scheme S1). This was then alkylated, at its free phenolic group, by the enantiopure propargyl-type alcohol (-)-(*S*)-**6**[2] under the Mitsunobu reaction conditions, this latter reactant undergoing inversion of configuration at its stereogenic centre in the process. This yielded the enantiopure triyne (-)-(*R*,*R*)-**7**. The final $Co^{I}$-catalysed [2+2+2] cycloisomerisation in a commercial microwave reactor led to the desired dioxa[7]helicene analogue (-)-(*M*,*R*,*R*)-**2** in a diastereo- and enantiomerically pure form. Although chirality was not an issue in the intended study on the through-space indirect NMR coupling, we sought to maintain the closest possible structural similarity of (-)-(*M*,*R*,*R*)-**2** to (-)-(*M*,*R*,*R*)-**1**, whose X-ray structure is known[4] (we were unfortunately unable to produce single crystals of (-)-(*M*,*R*,*R*)-**2** with high enough quality for X-ray analysis). To this end, the presence of the two stereogenic centres in (-)-(*M*,*R*,*R*)-**2** required the use of the enantiopure propargyl-type alcohol (-)-(*S*)-**6** to avoid the formation of a mixture of diastereomers during the synthesis. It is worth noting that the [2+2+2] cycloisomerisation reaction yielded a single diastereomer due to efficient stereocontrol by the 1,3-allylic-type strain.[5-7]

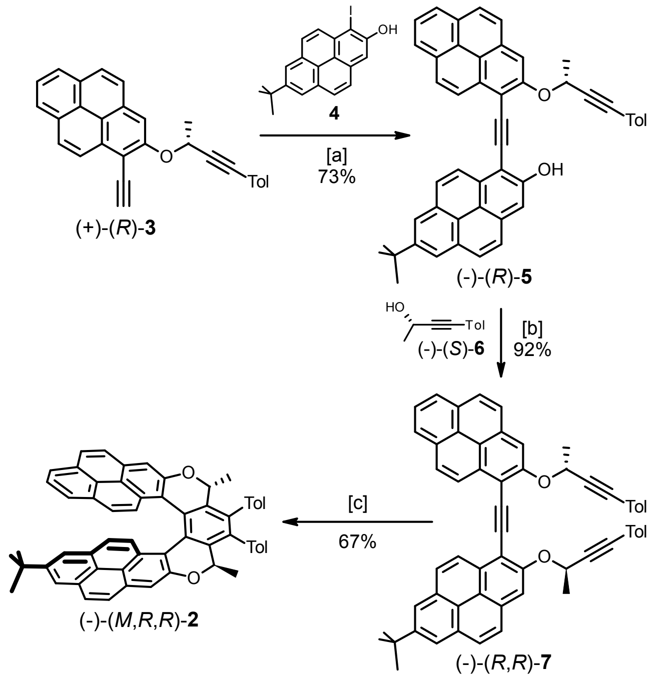


**Scheme S1** Synthesis of the unsymmetrical pyrene-based dioxa[7]helicene (-)-(*M*,*R*,*R*)-**2**. (a) Aryl iodide **4** (1.1 equiv.), $Pd(PPh_3)_2Cl_2$ (3 mol%), CuI (6 mol%), toluene-*i*-$Pr_2NH$ (4:1), RT, 1h; (b) alcohol (*S*)-**6** (1.1 equiv.), diisopropyl azodicarboxylate (DIAD, 1.2 equiv.), $PPh_3$ (2.2 equiv.), benzene, RT, 2.5 h; (c) $CpCo(CO)_2$(fum) (fum = dimethylfumarate, 20 mol%), 1-butyl-2,3-dimethylimidazolium tetrafluoroborate ([bdmim]$BF_4$, 20 mg/mL), microwave reactor, THF, 140 $^{o}$C, 10 min. Tol = *p*-tolyl.

**Diyne (+)-(*R*)-3**

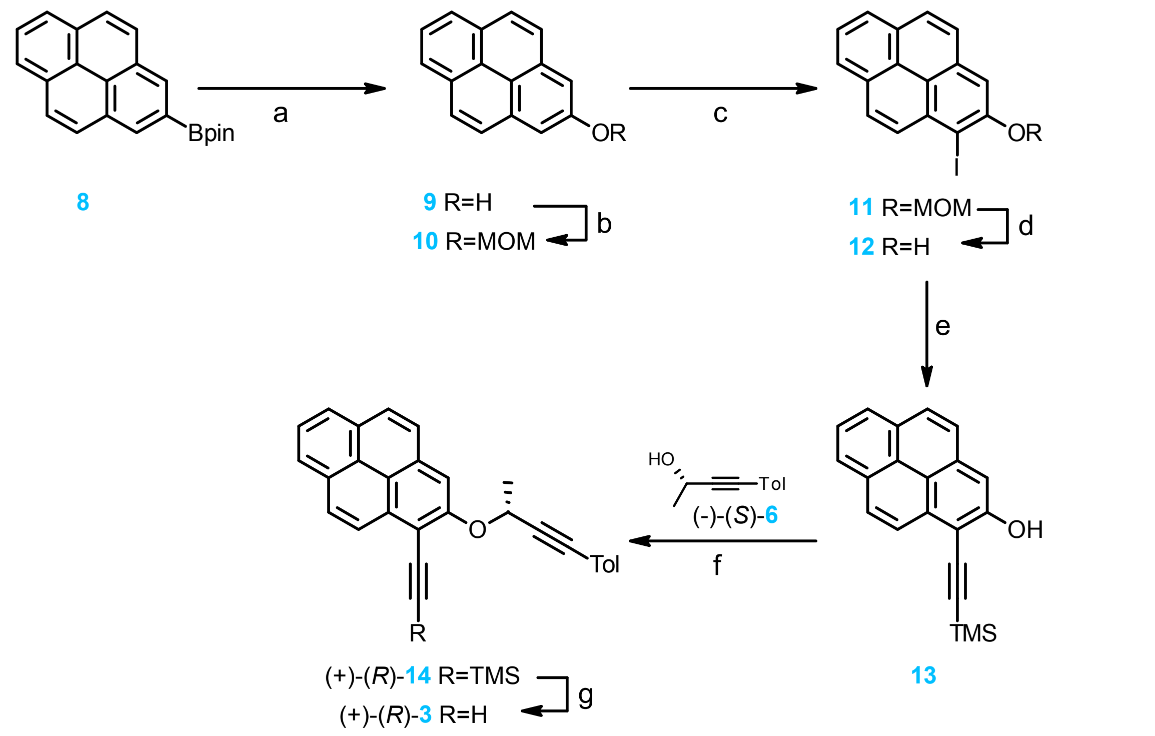


(a) $H_2O_2$ (3.0 equiv.), KOH (3.0 equiv.), THF-water (5:2), rt, 1 h, 86%; (b) NaH (1.2 equiv.), $CH_3OCH_2Cl$ (1.2 equiv.), THF, 0 °C to rt, 1.5 h, 75%; (c) *n*-BuLi (1.1 equiv.), THF, -78 °C to -20 °C, 1 h, then $I_2$ (1.2 equiv.), -40 °C to rt, 50 min, 69%; (d) HCl (aq.), methanol, 60 °C, 4 h, used further without purification; (e) TMS-C≡CH (1.2 equiv.), $Pd(PPh_3)_2Cl_2$ (3 mol%), CuI (6 mol%), *i*-$Pr_2NH$-toluene (15:2), rt, 1 h, 75% (over two steps); (f) (-)-(*S*)- **6**[2] (1.1 equiv.), DIAD (1.2 equiv.), $PPh_3$ (1.1 equiv.), benzene, rt, 10 min, 98%; (g) $K_2CO_3$ (2.0 equiv.), methanol, rt, 4 h, 98%.

**Pyren-2-ol 9**[8]

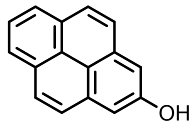


The pyrene derivative **8**[3] (1.00 g, 3.05 mmol) and potassium hydroxide (513 mg, 9.14 mmol, 3.0 equiv.) were dissolved in a mixture of tetrahydrofuran-water (70 ml, 5:2). To this mixture, a solution of hydrogen peroxide (30 wt %, 934 µl, 9.14 mmol, 3.0 equiv.) was slowly added and stirred at room temperature for 1 h. It was purified by flash chromatography on silica gel (hexane-dichloromethane 1:9) to give product **9** (572 mg, 86%). The spectral and physical data were in agreement with literature.[8]

**2-(Methoxymethoxy)pyrene 10**

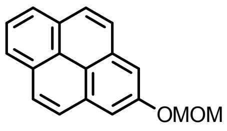


To a solution of pyren-2-ol **9** (552 mg, 2.53 mmol) in tetrahydrofuran (60 ml) at 0 °C, sodium hydride (95%, 76.6 mg, 3.03 mmol, 1.2 equiv.) was carefully added and the reaction mixture was stirred at 0 °C for 30 min. Then methoxymethyl chloride (256 µl, 3.03 mmol, 1.2 equiv.) was added and the reaction mixture was stirred at 0 °C for 1.5 h. The reaction mixture was quenched with a saturated solution of $NaHCO_3$ (60 ml), extracted with dichloromethane (3 × 30 ml), the combined organic phases were washed with

water (3 × 30 ml) and dried over anhydrous $MgSO_4$. The solvent was removed *in vacuo*. Column chromatography on silica gel (hexane-dichloromethane 2:3) gave **10** (500 mg, 75%) as white crystals.
**Mp**: 80-82 °C (dichloromethane-hexane).
**$^1$H NMR** (400 MHz, $CDCl_3$) and **$^{13}$C NMR** (101 MHz, $CDCl_3$) data:

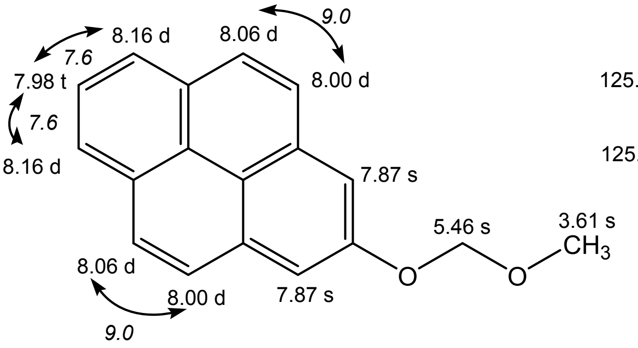


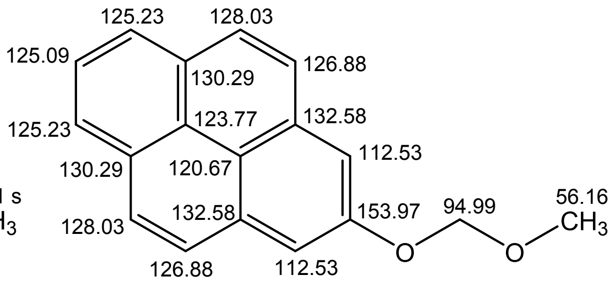


**IR** ($CHCl_3$): 3045 w, 1642 w, 1601 m, 1559 m, 1477 w, 1448 m, 1441 m, 1402 q, 1153 s, 1136 m, 1079 m, 1071 m, 1026 s, 998 m, 921 m, 870 m, 709 vs cm$^{-1}$.
**APCI MS**: 263 ([M+H]$^+$).
**HR APCI MS**: calcd for $C_{18}H_{15}O_2$ 263.1067, found 263.1067.

### 1-Iodo-2-(methoxymethoxy)pyrene 11

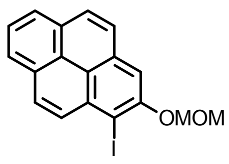


To a solution of derivative **10** (500 mg, 1.91 mmol) in tetrahydrofuran (20 ml) at -78 °C under argon a solution of *n*-butyllithium (1.6 M in hexanes, 1.31 ml, 2.10 mmol, 1.1 equiv.) was carefully added and the reaction was stirred at -20 °C for 1 h. The reaction mixture was then cooled to -40 °C and a solution of iodine (581 mg, 2.29 mmol, 1.2 equiv.) in tetrahydrofuran (15 ml) was added over a period of 10 min. After stirring at room temperature for another 50 min, a saturated solution of $NH_4Cl$ (50 ml) was added. The reaction mixture was extracted with ethyl acetate (3 × 50 ml), the combined organic phases were washed with a solution of $Na_2S_2O_3$ (10 wt %, 50 ml), water (2 × 50 ml), brine (50 ml) and dried over anhydrous $Na_2SO_4$, filtered and evaporated to dryness. Column chromatography on C-18 coated silica gel (acetonitrile) gave product **11** (510 mg, 69%) as white crystals.
**Mp**: 120-122 °C (acetonitrile).
**$^1$H NMR** (500 MHz, $CDCl_3$) and **$^{13}$C NMR** (126 MHz, $CDCl_3$) data:

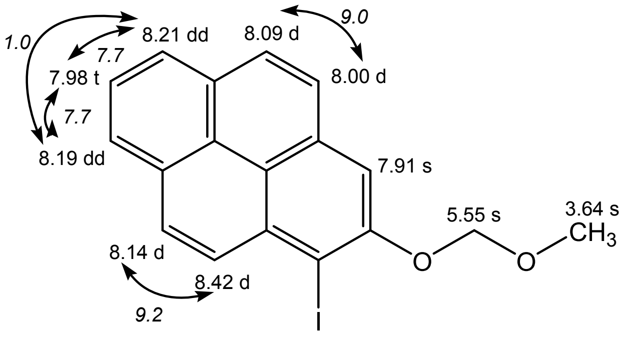


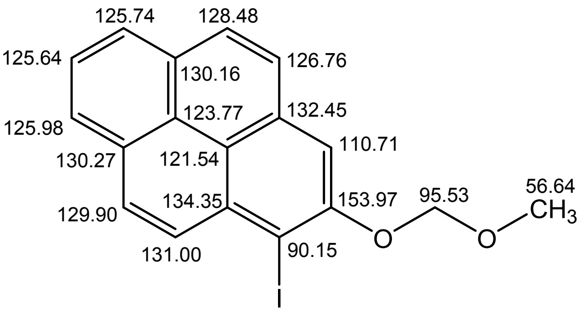

**IR** ($CHCl_3$): 3045 w, 2850 m, 2830 w, sh, 1626 w, 1587 m, 1545 m, 1482 w, sh, 1464 w, 1429 m, 1282 s, 1153 s, 1094 m, 1028 vs, 932 m, 882 w, 839 s, 709 m cm$^{-1}$.
**TOF EI MS**: 388 ($M^{+\bullet}$, 100).
**HR TOF EI MS**: calcd for $C_{18}H_{13}O_2I$ 387.9960, found 387.9955.

**1-Iodopyren-2-ol 12**

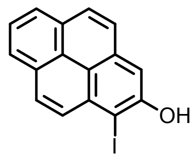


MOM-Derivative **11**(was dissolved in tetrahydrofuran (15 ml) and conc. HCl (0.7 ml) was added. The reaction mixture was stirred at 60 °C for 4 h. After cooling to the room temperature, the reaction mixture was diluted by dichloromethane (20 ml) and washed with water (3 x 20 ml), brine (20 ml) and dried over anhydrous $Na_2SO_4$. The solvent was removed at reduced pressure and the crude product **12** was used without further purification in the following step.

**1-[(Trimethylsilyl)ethynyl]pyren-2-ol 13**

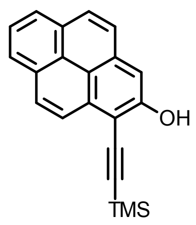


A mixture of $Pd(PPh_3)_2Cl_2$ (17 mg, 0.024 mmol, 3 mol%), CuI (9.3 mg, 0.049 mmol, 6 mol%) and iodo derivative **12** (280 mg, 0.814 mmol) in diisopropylamine (15 ml) and toluene (2 ml) was stirred at room temperature for 20 min. Then (trimethylsilyl)acetylene (137 µl, 0.976 mmol, 1.2 equiv.) was added over a period of 10 min and the resulting reaction mixture was stirred under argon at room temperature for 1 h. The reaction mixture was diluted by ethyl acetate (30 ml) and washed by a saturated solution of $NH_4Cl$ (3 x 20 ml), brine (20 ml) and dried over anhydrous $Na_2SO_4$, filtered and evaporated to dryness. Column chromatography on silica gel (hexane-dichloromethane 3:2) afforded the protected alkyne **13** (191 mg, 75 %, over 2 steps) as a yellowish amorphous solid.
**Mp**: 87-89 °C (dichloromethane-hexane).
**$^1$H NMR** (400 MHz, $CDCl_3$) and **$^{13}$C NMR** (101 MHz, $CDCl_3$) data:

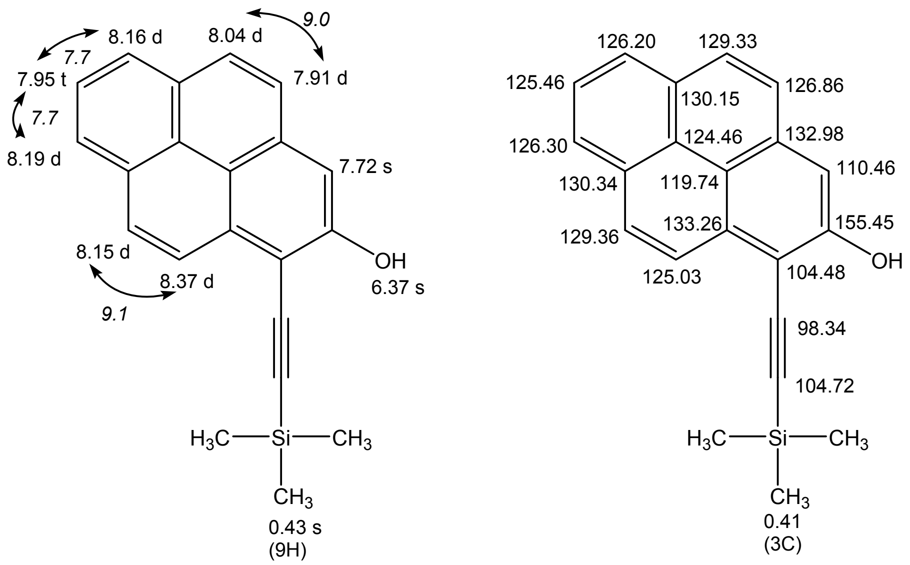


**IR** ($CHCl_3$): 3508 m, 3050 w, 2961 m, 2900 w, 2141 m, 1638 m, 1599 m, 1542 m, 1438 s, 1404 m, 1253 s, 1140 s, 985 w, 882 s, 843 s cm$^{-1}$.
**TOF EI MS**: 314 ($M^{+\bullet}$, 100), 299 (28).
**HR TOF EI MS**: calcd for $C_{21}H_{18}OSi$ 314.1127, found 314.1124.

**(+)-Trimethyl[(2-{[(1*R*)-1-methyl-3-(4-methylphenyl)prop-2-yn-1-yl]oxy}pyren-1-yl)ethy-nyl]silane 14**

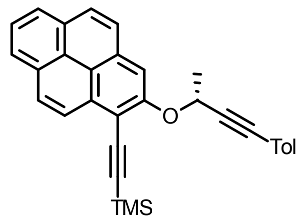


A mixture of TMS-derivative **13** (95 mg, 0.30 mmol), triphenylphosphine (87 mg, 0.33 mmol, 1.1 equiv.) and (-)-(*S*)-**6**[2] (53 mg, 0.33 mmol, 1.1 equiv.) in benzene (3 ml) in a Schlenk flask was purged with argon, then diisopropyl azodicarboxylate (71 µl, 0.36 mmol, 1.2 equiv.) was added. After stirring at room temperature under argon atmosphere for 10 min, the reaction mixture was evaporated to dryness and the residue was purified by column chromatography on silica gel (hexane-ethyl acetate 9:1) to obtain product (+)-(*R*)-**14** (135 mg, 98%) as a yellow-orange oil.

**Optical rotation**: $[\alpha]^{20}_{D}$ = + 138° (c 0.291, $CHCl_3$).

**$^1$H NMR** (600 MHz, $CDCl_3$) and **$^{13}$C NMR** (151 MHz, $CDCl_3$) data:

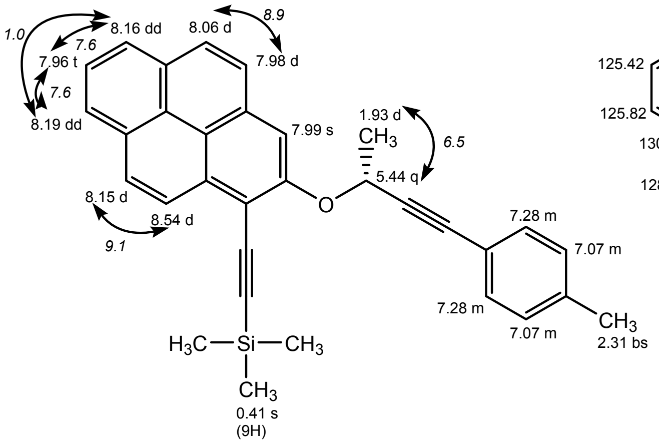


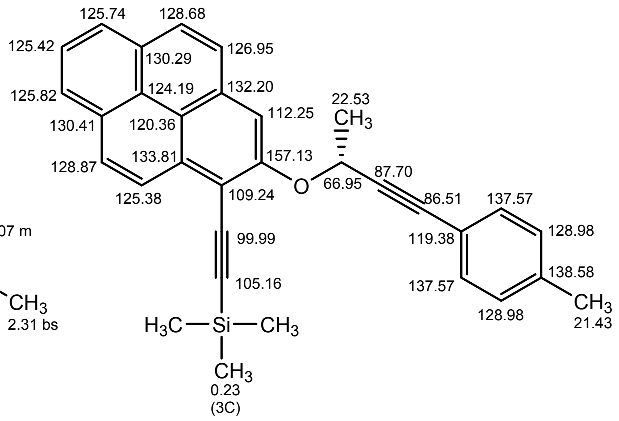


**IR** ($CHCl_3$): 3047 w, 2900 w, 2147 m, 1625 w, 1596 m, 1584 w, 1539 m, 1510 m, 1440 m, 1409 w, 1332 w, 1309 m, 1285 m, 1252 m, 1231 m, 1181 m, 1047 m, 999 w, 971 w, 888 m, 853 s, 844 s, 830 m, 688 m, 544 w $cm^{-1}$.

**APCI MS**: 457 ($[M+H]^+$).

**HR APCI MS**: calcd for $C_{32}H_{29}OSi$ 457.1982, found 457.1981.

**(+)-1-Ethynyl-2-{[(1*R*)-1-methyl-3-(4-methylphenyl)prop-2-yn-1-yl]oxy}pyrene 3**

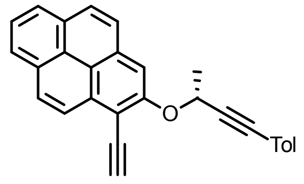


To a solution of diyne (+)-(*R*)-**14** (130 mg, 0.285 mmol) in methanol (20 ml) potassium carbonate was added (79 mg, 0.57 mmol, 2.0 equiv.) in one portion. The reaction mixture was stirred at room temperature for 4 h and then filtered through a plug of silica gel (6 × 2 cm, dichloromethane). The solvents were evaporated and the residue was purified by column chromatography on silica gel (hexane-dichloromethane 4:1) to furnish the product (+)-(*R*)-**3** (107 mg, 98%) as yellowish crystals.

**Mp**: 150-152 °C (dichloromethane-hexane).

**Optical rotation**: $[\alpha]^{20}_{D}$ = + 273° (c 0.234, $CHCl_3$).

**$^1$H NMR** (400 MHz, $CDCl_3$) and **$^{13}$C NMR** (101 MHz, $CDCl_3$) data:

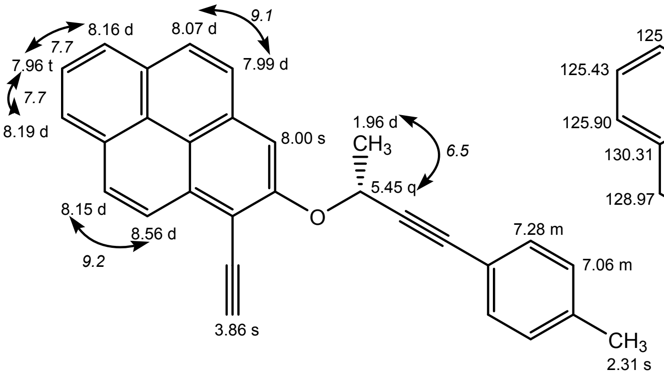


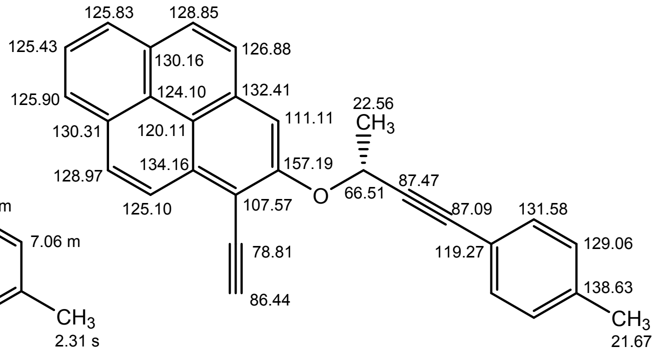


**IR** ($CHCl_3$): 3305 m, 3048 w, 2869 w, 2229 w, 2100 w, 1623 w, 1596 m, 1585 m, 1539 m, 1510 m, 1441 m, 1417 m, 1387 w, 1332 w, 1305 w, 1282 vs, 1260 w, 1088 s, 1046 m, 854 s, 688 w $cm^{-1}$.

**APCI MS**: 385 ($[M+H]^+$).

**HR APCI MS**: calcd for $C_{29}H_{21}O$ 385.1587, found 385.1588.

**Dioxa[7]helicene analogue (-)-(*M*,*R*,*R*)-2**

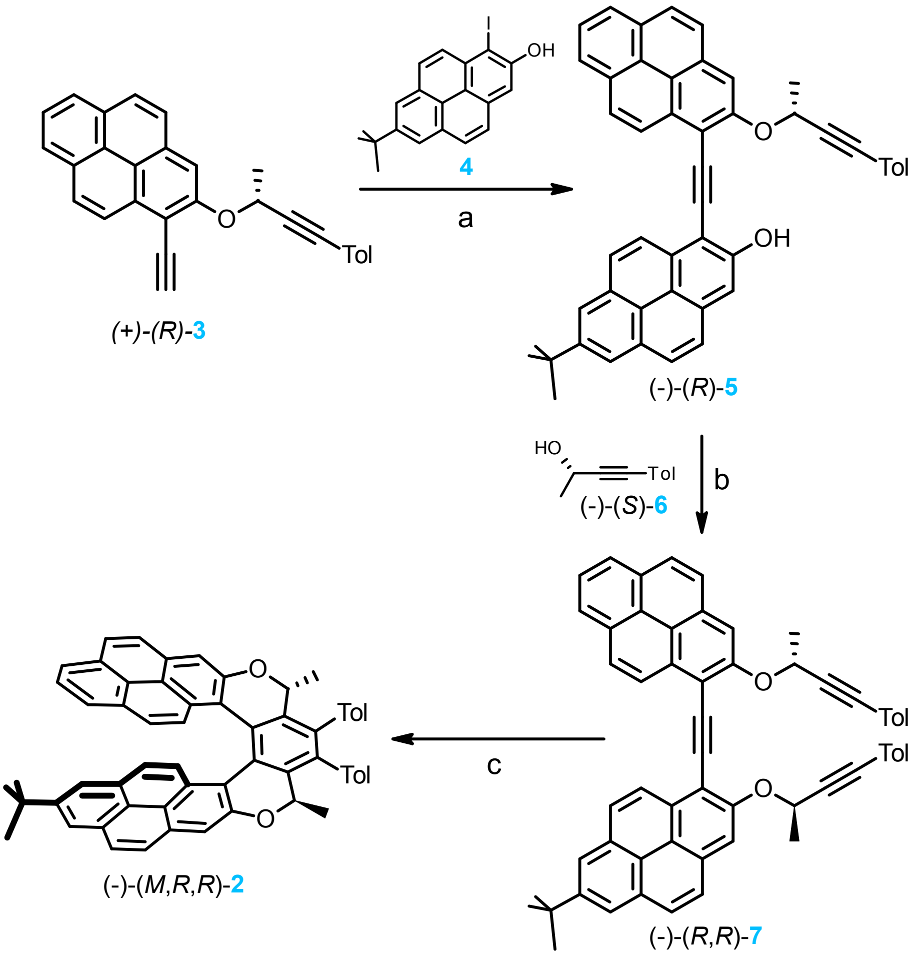


(a) **4** (1.1 equiv.), (Pd($PPh_3$)$_2$$Cl_2$ (3 mol%), CuI (6 mol%), toluene-*i*-$Pr_2$NH (4:1), rt, 1 h, 73%; (b) (-)-(*S*)-**6**[2] (1.1 equiv.), DIAD (1.2 equiv.), $PPh_3$ (2.2 equiv.), benzene, RT, 2.5 h, 92%; (c) CpCo(CO)(fum) (20 mol%), [bdmim]$BF_4$ (20 mg/ml), microwave reactor, THF, 140 °C, 10 min, 67%.

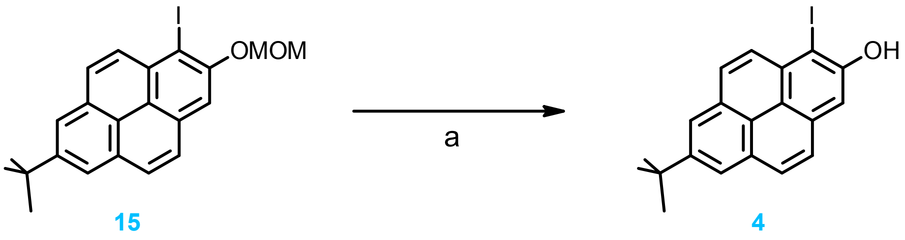


(a) HCl (aq.), methanol, 60 °C, 4 h, used further without purification.

**7-*tert*-Butyl-1-iodopyren-2-ol 4**

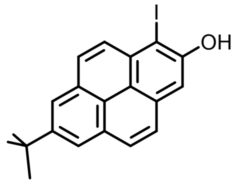


MOM-Derivative **15**[4] (470 mg, 1.06 mmol) was dissolved in tetrahydrofuran (15 ml) and conc. HCl (36 wt%, 0.7 ml) was added. The reaction mixture was stirred at 60 °C for 4 h. After cooling to the room temperature, the reaction mixture was diluted by dichloromethane (20 ml) and washed with water (3 × 20 ml),

brine (1 × 20 ml) and dried over anhydrous $Na_2SO_4$. The solvent was removed under stream of nitrogen and the crude product **4** (420 mg) was used without further purification in the following step. Only a small sample of **4** (40 mg) was purified by column chromatography (hexane-dichloromethane 7:3) for characterisation.
**Mp**: 168-170 °C (dichloromethane-hexane).
**[1]H NMR** (600 MHz, $CDCl_3$) and **[13]C NMR** (151 MHz, $CDCl_3$) data:

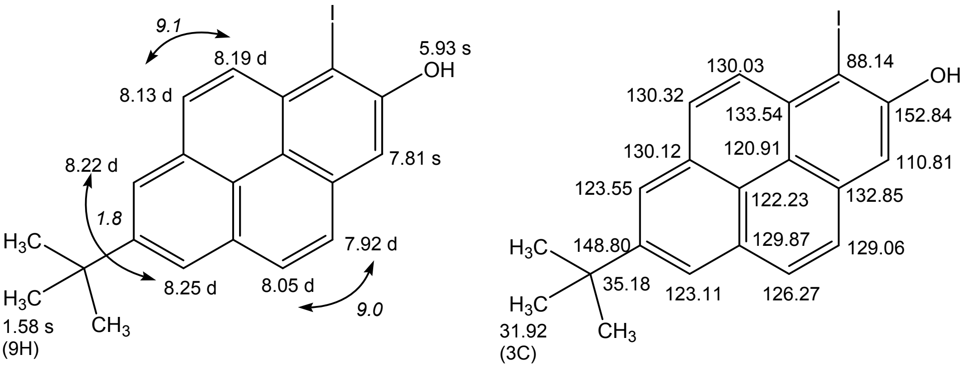


**IR** ($CHCl_3$): 3589 w, 3487 m, 3048 vw, 2967 s, 2868 w, 1633 vw, 1598 vs, 1554 m, 1479 m, 1437 s, 1413 s, 1395 w, 1363 w, 1288 w, 921 w, 882 vs, 694 w cm$^{-1}$.
**TOF EI MS**: 400 ($M^{+\bullet}$, 100), 385 (82), 273 (7), 258 (23), 243 (17), 189 (13), 178 (14), 127 (8).
**HR TOF EI MS**: calcd for $C_{20}H_{17}OI$ 400.0324, found 400.0328.

**(-)-7-*tert*-Butyl-1-[(2-{[(1*R*)-1-methyl-3-(4-methylphenyl)prop-2-yn-1-yl]oxy}pyren-1-yl)ethynyl]pyren-2-ol 5**

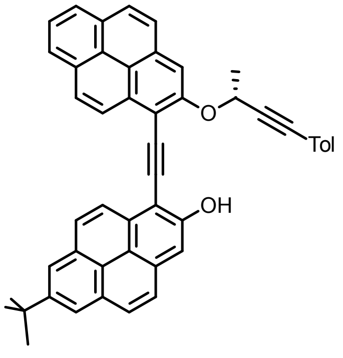


A mixture of $Pd(PPh_3)_2Cl_2$ (2.59 mg, 3.69 µmol, 3 mol%), CuI (1.41 mg, 7.40 µmol, 6 mol%) and iodopyrenol **4** (49.24 mg, 0.1230 mmol, 1.1 equiv.) in diisopropylamine (0.5 ml) and toluene (1 ml) was stirred at room temperature under an argon atmosphere for 10 min. A solution of alkyne (+)-(*R*)-**3** (43 mg, 0.11 mmol) in toluene (1 ml) was added dropwise over a period of 10 min and the reaction mixture was stirred at room temperature under an argon atmosphere for 1 h. The reaction mixture was poured into a saturated solution of $NH_4Cl$ (20 ml) and extracted with ethyl acetate (2 × 10 ml). The combined organic phases were washed with a saturated solution of $NH_4Cl$ (2 × 20 ml), dried over anhydrous $MgSO_4$, filtered and evaporated to dryness. Column chromatography on silica gel (hexane-ethyl acetate 4:1) afforded product (-)-(*R*)-**5** (59 mg, 73%) as a yellow powder.
**Mp**: 171-173 °C (dichloromethane-hexane).
**Optical rotation**: $[\alpha]^{20}_D$ = - 72.2° (c 0.313, $CHCl_3$).
**[1]H NMR** (600 MHz, $CDCl_3$) and **[13]C NMR** (151 MHz, $CDCl_3$) data:

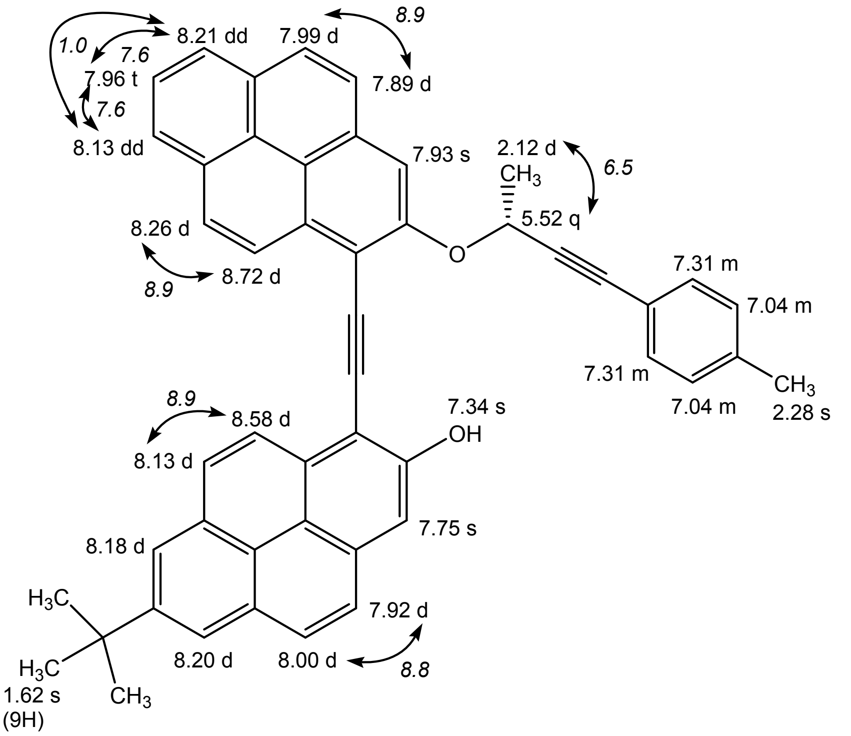


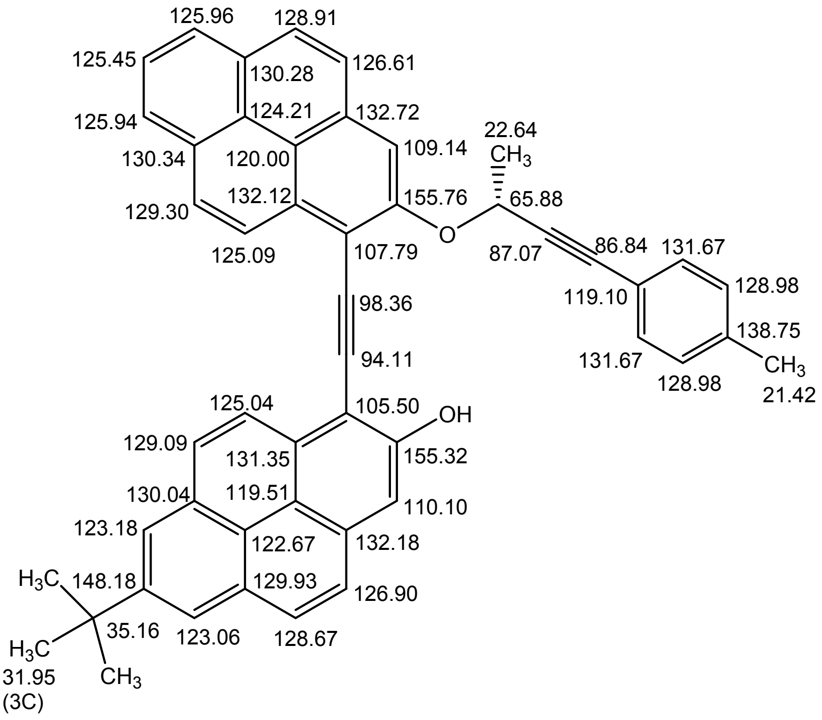


**IR** ($CCl_4$): 3447 m, 3047 m, 2965 s, 2956 s, 2231 w, 2177 vw, 1633 w, 1610 m, 1595 s, 1585 m, 1542 m, 1511 m, 1480 w, 1458 m, 1445 s, 1410 m, 1394 w, 1378 m, 1362 w, 1331 m, 1309 m, 1285 vs, 1226 s, 1180 w, 1147 m, 1121 m, 1085 s, 1021 m, 880 s, 615 w $cm^{-1}$.

**ESI MS**: 679 ($[M+Na]^+$).

**HR ESI MS**: calcd for $C_{49}H_{36}O_2Na$ 679.2608, found 679.2608.

**(-)-7-*tert*-Butyl-2-{[(1*R*)-1-methyl-3-(4-methylphenyl)prop-2-yn-1-yl]oxy}-1-[(2-{[(1*R*)-1-methyl-3-(4-methylphenyl)prop-2-yn-1-yl]oxy}pyren-1-yl)ethynyl]pyrene 7**

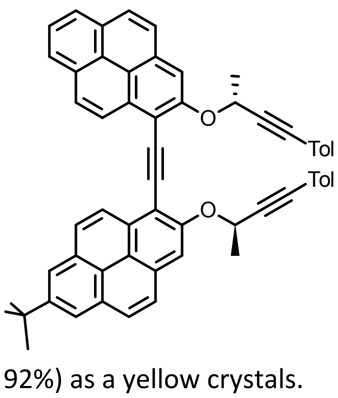


A suspension of diyne (-)-(*R*)-**5** (41 mg, 0.062 mmol), triphenylphosphine (18.01 mg, 0.181 mmol, 2.2 equiv.) and (-)-(*S*)-**6**[2] (31.7 mg, 0.0687 mmol, 1.1 equiv.) in benzene (1 ml) in a Schlenk flask was purged with argon, then diisopropyl azodicarboxylate (14.8 µl, 0.0749 mmol, 1.2 equiv.) was added. The orange suspension turned to a wine red solution. After stirring at room temperature under argon atmosphere for 2.5 h, the reaction mixture was evaporated to dryness and the residue was purified by column chromatography on silica gel (hexane-dichloromethane 7:3) to obtain the product (-)-(*R*,*R*)-**7** (46 mg, 92%) as a yellow crystals.

**Optical rotation**: $[\alpha]^{20}_{D}$ = - 272° (c 0.279, $CHCl_3$).

**Mp**: 100-102 °C (dichloromethane-hexane).

**$^{1}$H NMR** (400 MHz, $CDCl_3$): 1.62 (s, 9H), 2.19 (d, *J* = 6.5 Hz, 3H), 2.20 (d, *J* = 6.5 Hz, 3H), 2.30 (s, 6H), 5.60 (m, 2H), 7.06 (m, 4H), 7.33 (m, 4H), 7.93-8.07 (m, 7H), 8.14-8.18 (m, 4H), 8.22 (s, 2H), 9.10 (d, *J* = 9.1 Hz, 1H), 9.11 (d, *J* = 1.9 Hz, 1H).

**$^{13}$C NMR** (101 MHz, $CDCl_3$): 21.42, 21.43, 22.88, 22.91, 31.93 (3C), 35.16, 65.96, 65.99, 86.52, 86.54, 87.72, 87.80, 95.62, 95.95, 109.55, 109.83, 110.15, 110.20, 119.37, 120.21, 120.24, 122.55, 122.82, 122.95, 124.30, 125.27, 125.53, 125.60, 126.06, 126.18, 126.89, 127.00, 128.36, 128.61, 128.62, 128.90, 128.95 (4C), 130.28, 130.31, 130.41, 130.45, 131.65 (2C), 131.69 (2C), 131.83, 131.96, 133.28, 133.40, 138.55, 138.58, 148.38, 156.06, 156.20.

**IR** ($CHCl_3$): 3047 w, 2991 m, 2966 s, 2230 w, 2200 vw, 1601 m, 1585 m, sh, 1510 s, 1479 w, 1409 w, 1395 m, 1375 w, 1363 w, 1329 m, 1261 m, 1185 s, 1118 m, 1090 vs, 1021 m, 818 s, 645 w $cm^{-1}$.

**UV-Vis** (THF): $\lambda_{max}$ (log $\varepsilon$) = 252 (5.16), 304 (4.58), 400 (4.57), 426 (4.71), 452 nm (4.87).

**Fluorescence** (THF, $\lambda_{exc}$ 452 nm): $\lambda_{max}$ ($I_{rel}$) 467 (1.00), 496 nm (0.62).

**APCI MS**: 799 ($[M+H]^+$).

**HR ESI MS**: calcd for $C_{60}H_{47}O_2$ 799.3571, found 799.3572.

**(-)-(*M*,17*R*,20*R*)-4-*tert*-Butyl-17,20-dimethyl-18,19-bis(4-methylphenyl)-17,20-dihydrodi-phenaleno[1,9-*fg*:1,9-*f'g'*]benzo[1,2-*c*:4,3-*c'*]dichromane 2**

A suspension of triyne (-)-(*R*,*R*)-**7** (42 mg, 0.053 mmol), CpCo(CO)(fum) (3.13 mg, 0.0105 mmol, 20 mol%), [bdmim]$BF_4$ (50 mg) in tetrahydrofuran (2.5 ml) was heated in a microwave reactor at 140 °C for 10 min. After cooling to room temperature, the solvent was evaporated and the residue was purified by column chromatography on silica gel (hexane-ethyl acetate 9:1) to afford product (-)-(*M*,*R*,*R*)-**2** (20 mg, 67%) as a yellow powder.

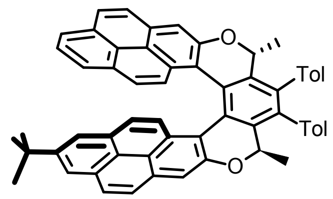


**Optical rotation**: $[\alpha]^{20}_{D}$ = - 2487° (c 0.747, $CHCl_3$).

**Mp**: >350 °C (chloroform-hexane).

**$^{1}$H NMR** (600 MHz, $CDCl_3$) and **$^{13}$C NMR** (151 MHz, $CDCl_3$) data:

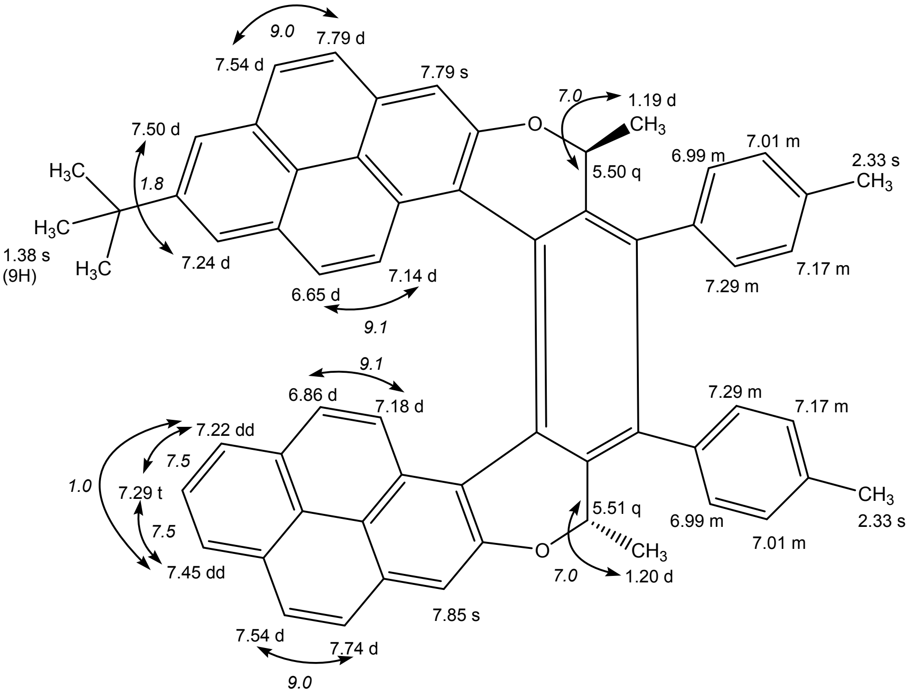


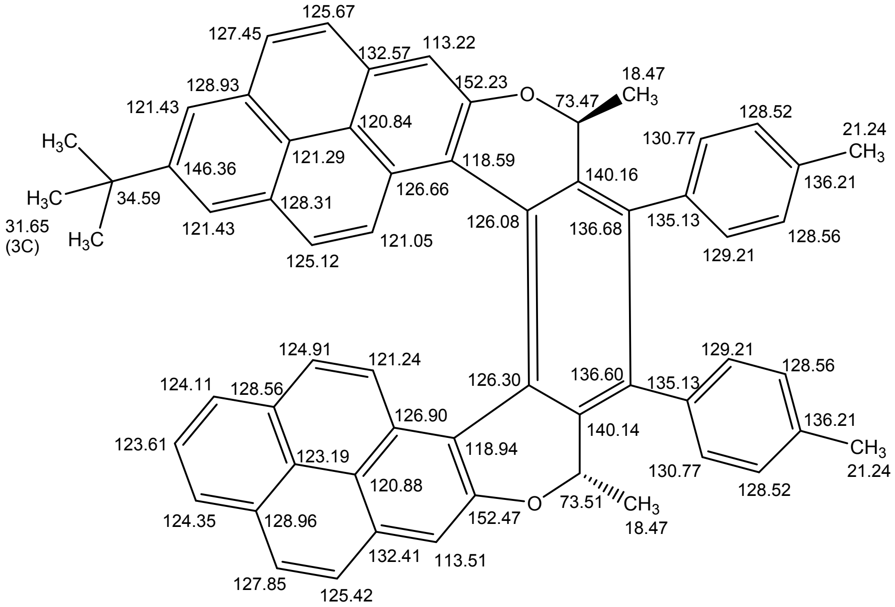


**IR** ($CHCl_3$): 2965 m, 1633 w, sh, 1601 m, 1544 w, 1517 w, 1493 w, 1479 w, 1404 m, 1394 m, 1375 m, 1368 w, 1277 s, 1148 m, 1111 w, 1057 m, 1020 m, 880 m, 828 m, 813 m, 699 m, 532 w $cm^{-1}$.

**UV-Vis** (THF): $\lambda_{max}$ (log $\varepsilon$) = 248 (4.82), 299 (4.42), 372 (4.71), 404 nm (4.21).

**Fluorescence** (THF, $\lambda_{exc}$ 396 nm): $\lambda_{max}$ ($I_{rel}$) 573 (1.00), 458 nm (0.29).

**APCI MS**: 799 ($[M+H]^+$).

**HR APCI MS**: calcd for $C_{60}H_{47}O_2$ 799.3571, found 799.3572.

## NMR Spectroscopy

NMR spectra were measured on a Bruker Avance III 500, Avance III 600 and/or Avance III 850 spectrometer (500.0, 600.1 or 850.3 MHz for $^1$H and 125.7, 150.9 or 213.8 MHz for $^{13}$C) equipped with a 5 mm PFG cryoprobe. All spectra were acquired for samples in $CDCl_3$ and referenced to TMS. Signals of all hydrogen and carbon atoms were assigned by using a combination of 1D and 2D (H,HCOSY, H,C-HSQC, H,C-HMBC, and H,H-ROESY) techniques. The long-range COSY (LR-COSY) experiment was recorded by using the standard magnitude-mode COSY sequence and setting the delay for evolution of long-range couplings to 800 ms. Eight kilo data points were acquired in the $F_2$ dimension and 350 increments in the $F_1$ dimension. Spectral window of 10 ppm x 10 ppm, pre-acquisition delay of 1s and 48 scans were used. The small through-space couplings were obtained from selective 1D homonuclear decoupling experiments, in which the data were reproduced iteratively by simulations with different coupling-constant values. Homonuclear *J*-resolved experiment was performed with the standard pulse sequence (jresqf). The spectral width in the indirect dimension (the dimension of H–H coupling) was 24 Hz. 128 increments were used leading to spectral resolution in the indirect dimension of 0.38 Hz).

### NMR spectroscopy experiments with compound (-)-(*M*,*R*,R)-3

The assignment of $^1$H and $^{13}$C NMR signals is based on a combination of 1D ($^1$H , $^{13}$C-APT) and 2D homo- and hetero-nuclear NMR experiments (H,H-COSY, H,C-HSQC and H,C-HMBC). Selected indicative HMBC contacts are shown in Figure S1.

$^1$H NMR (500 MHz, $CDCl_3$): 0.93 (d, 3H, $J_{CH3,7'}$ = 6.7, $CH_3$'), 0.96 (d, 3H, $J_{CH3,7}$ = 6.7, $CH_3$), 2.26 (s, 6H, $CH_3$-Tol), 3.82 (s, 3H, $OCH_3$), 5.25 (q, 1H, $J_{7,CH3}$ = 6.7, H-7), 5.26 (q, 1H, $J_{7',CH3'}$ = 6.7, H-7'), 6.39 (dd, 1H, $J_{2,1}$ = 8.7, $J_{2,4}$ = 2.6, H-2), 6.57 (d, 1H, $J_{4,2}$ = 2.6, H-4), 6.65–6.68 (m, 2H, *o*-Tol), 6.80 (m, 1H, H-2'), 6.85–6.89 (m, 2H, *m*-Tol), 7.01 (dd, 1H, $J_{4',3'}$ = 8.1, $J_{4',2'}$ = 1.3, H-4'), 7.09 (m, 2H, *m*-Tol), 7.13–7.18 (m, 3H, H-3' and *o*-Tol), 7.37 (d, 1H, $J_{1,2}$ = 8.7, H-1), 7.52 (dd, 1H, $J_{1',2'}$ = 7.9, $J_{1',3'}$ = 1.6, H-1').

$^{13}$C NMR (126 MHz, $CDCl_3$): 18.29 and 18.33 ($CH_3$-7 and $CH_3$-7'), 21.17 ($CH_3$-Tol), 55.25 (OMe), 72.95 (C-7'), 73.13 (C-7), 103.81 (C-4), 107.60 (C-2), 116.24 (C-10), 119.11 (C-4'), 120.88 (C-2'), 123.72 (C-10'), 124.47 (C-9'), 125.36 (C-9), 128.34 and 128.54 (*m*-Tol), 128.77, 128.99, 129.02 and 129.04 (*o*-Tol, C-1' and C-3'), 130.03 (C-1), 130.67 and 130.75 (*o*-Tol), 134.87 and 134.90 (*i*-Tol), 135.93 and 135.95 (*p*-Tol), 136.30 (8'-C-*i*'), 137.06 (8-C-*i*), 137.79 (C-8), 139.01 (C-8'), 153.32 (C-5'), 154.73 (C-5), 160.44 (C-3).

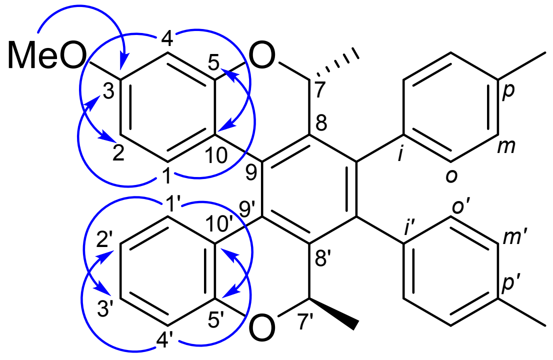


Figure S1. The structure of compound (-)-(*M*,*R*,R)-**3** with atom numbering and with selected indicative HMBC contacts used for unequivocal signal assignment. The HMBC contacts are indicated by blue arrows starting at a hydrogen atom and heading to a coupled carbon atom.

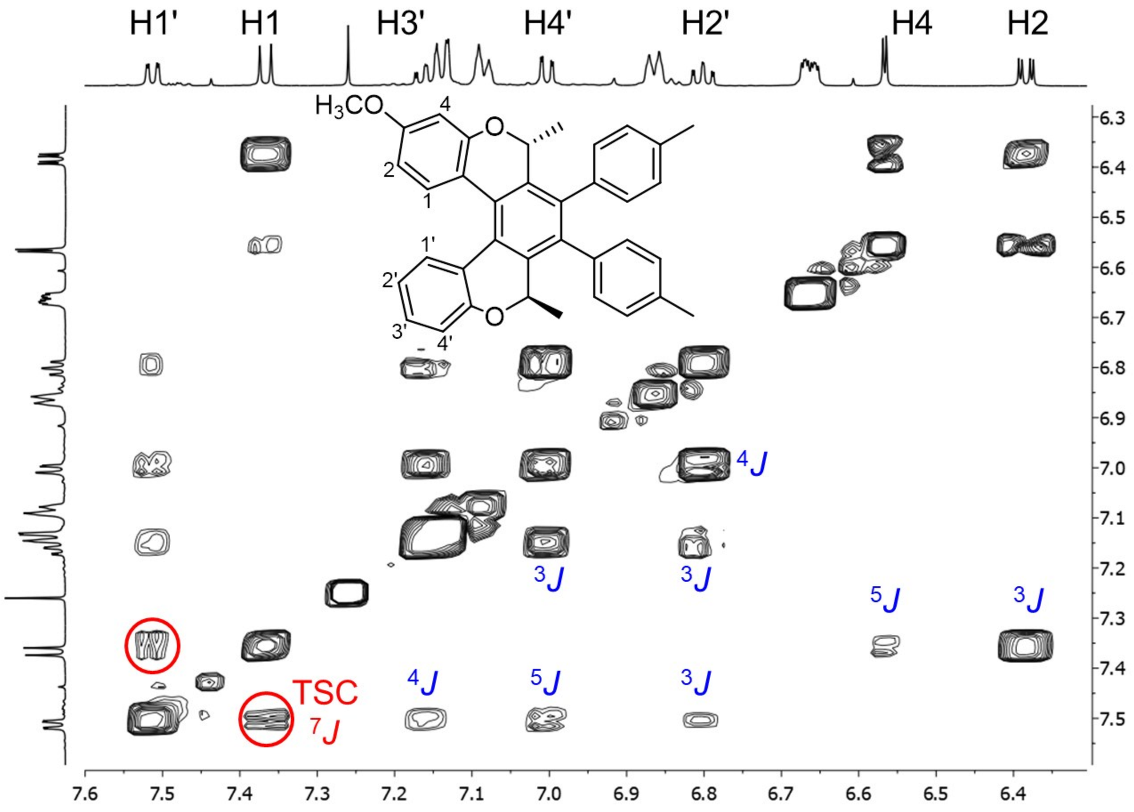


Figure S2. A part of the long-range COSY spectrum of compound (-)-(*M*,*R*,R)-**3** measured on a 600 MHz spectrometer in $CDCl_3$ with cross-peaks corresponding to through-space coupling highlighted by red circles.

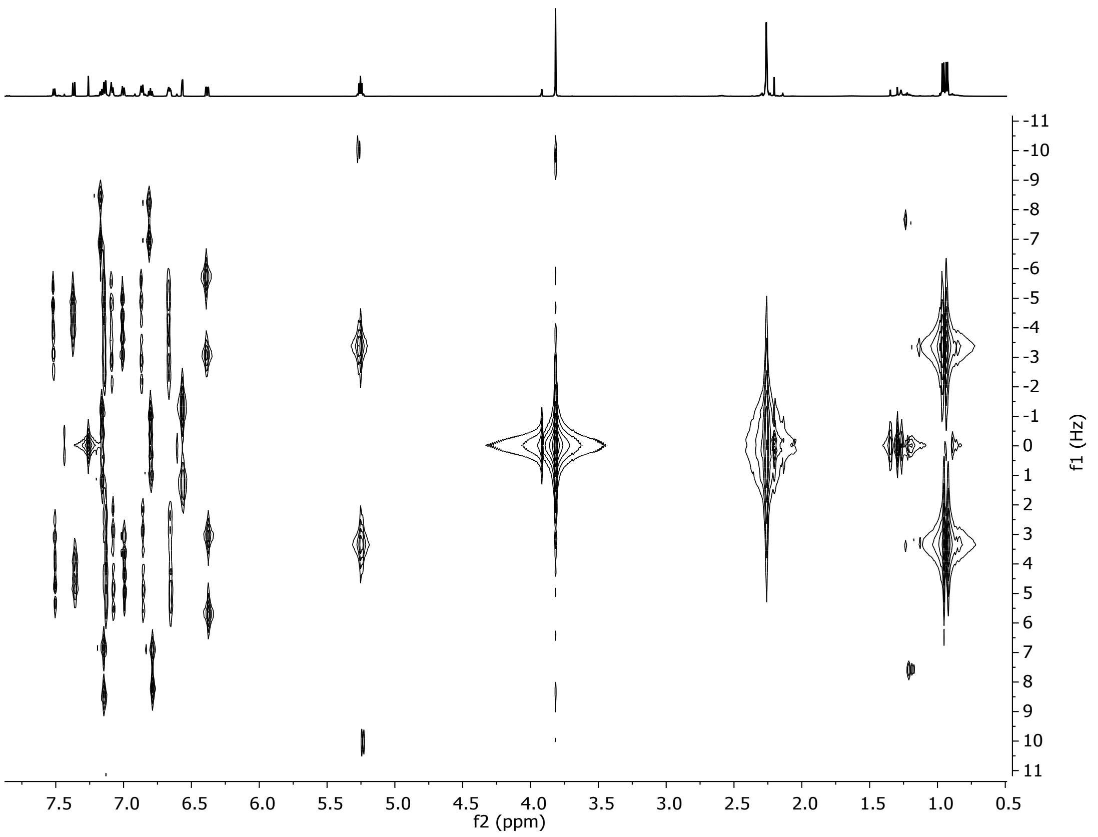


Figure S3. 2D homonuclear *J*-resolved NMR spectrum of compound (-)-(*M*,*R*,R)-**3**.

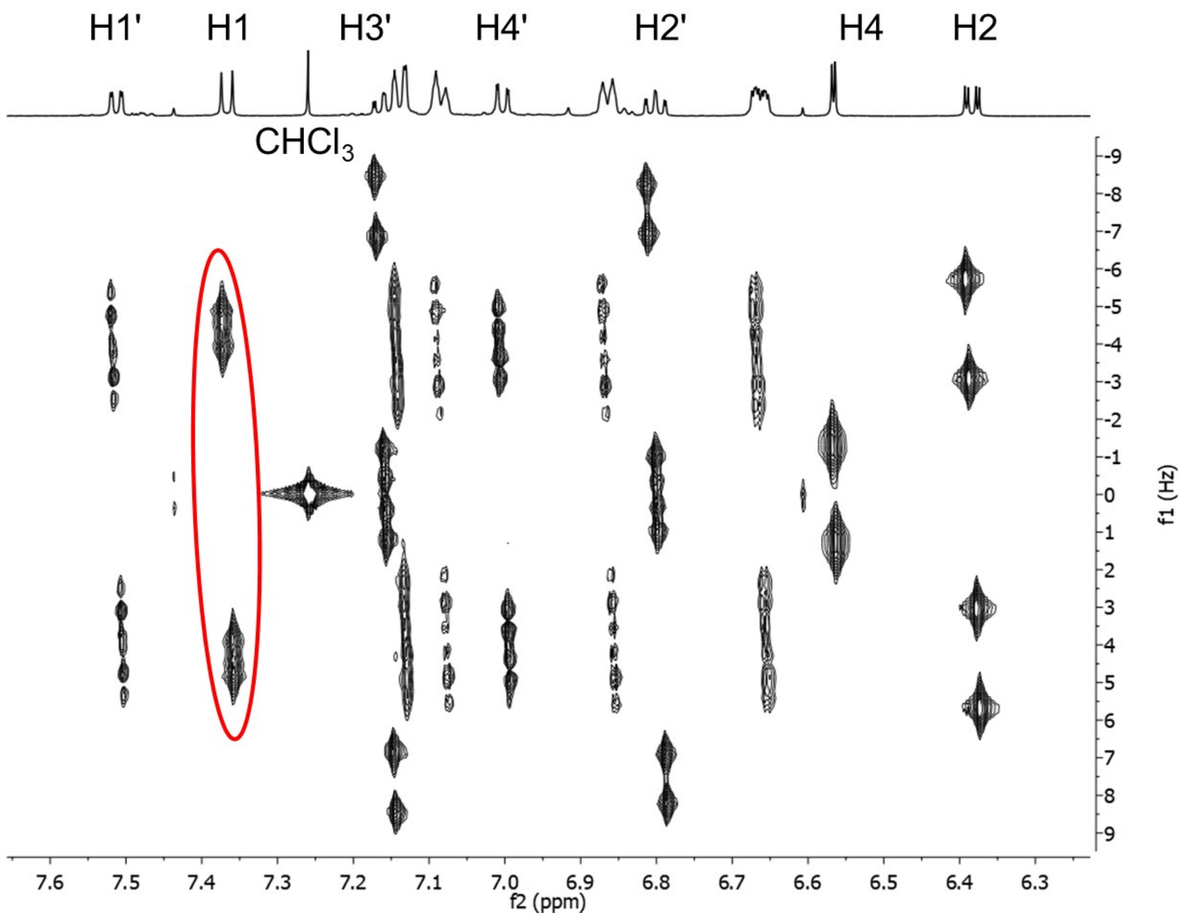


Figure S4. Expansion of the aromatic part of 2D homonuclear *J*-resolved NMR spectrum of compound (-)-(*M*,*R*,R)-**3**. The signals of H1, which were used for the plot in Figure 2 in the main text are highlighted.

**NMR spectroscopy experiments with compound (-)-(*M*,*R*,R)-2**

The assignment of $^{1}$H and $^{13}$C NMR signals is based on a combination of 1D ($^{1}$H , $^{13}$C-APT) and 2D homo- and hetero-nuclear NMR experiments (H,H-COSY, H,C-HSQC and H,C-HMBC). Signals of fragments A and B were unequivocally assigned with the help of the splitting patterns in $^{1}$H spectrum: hydrogen atoms A7 and A9 appear as doublets with small (four-bond) coupling of 1.8 Hz, whereas three-spin system was observed in fragment B (B7-B8-B9) with three-bond couplings of 7.56 Hz and four-bond coupling of 1.5 Hz. The assignments were also confirmed by heteronuclear C–H correlations and NOE experiments.

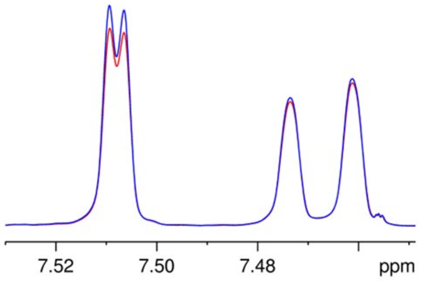


**Fig. S5.** $^{1}$H NMR spectrum of (-)-(*M*,*R*,*R*)-**2** (signals of A9 – left and B9 – right) with selective homonuclear decoupling of the signal of *tert*-butyl hydrogens (blue) and with the decoupler set to 0 ppm (red). 128 k data points were acquired with spectral window of 10 ppm. It has been reported previously[9] that fitting of experimental spectra with simulated signal line-shapes leads to accuracy of the spectral parameters (0.002–0.010 Hz), which is far better than the digital resolution.

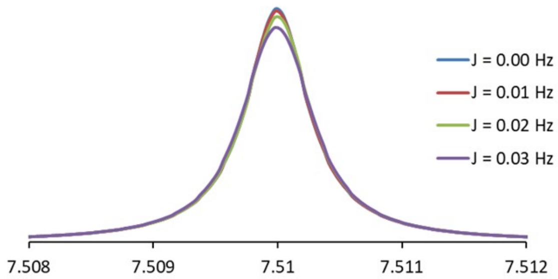


**Fig. S6.** Spectral simulation of a signal of an aromatic hydrogen coupled to nine equivalent aliphatic hydrogen atoms with different coupling constant. Line broadening of 0.3 Hz was used for the simulations. Comparison with the experimental spectra presented in Fig. S5 suggests that the TSC between the *tert*-butyl group and B9 is ca 0.02 Hz.

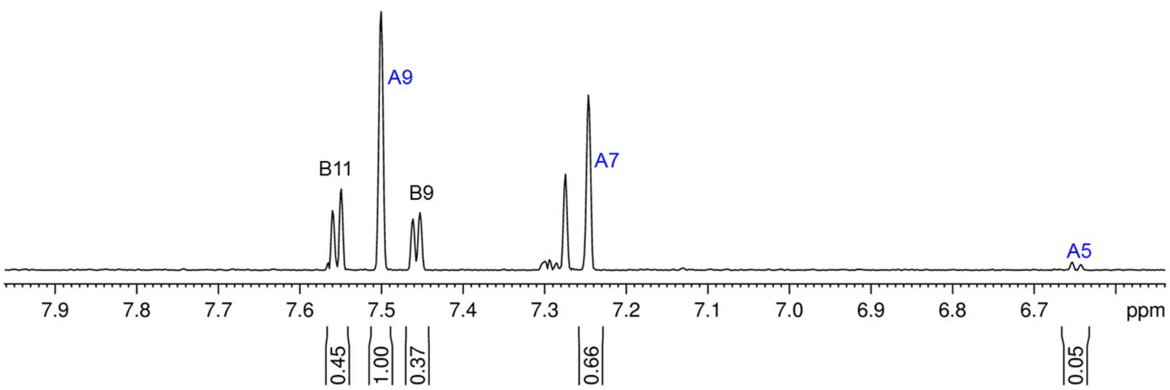


**Fig. S7.** The spectral row with interactions of the *tert*-butyl hydrogens extracted from long-range COSY spectrum of (-)-(*M*,*R*,*R*)-**2**.

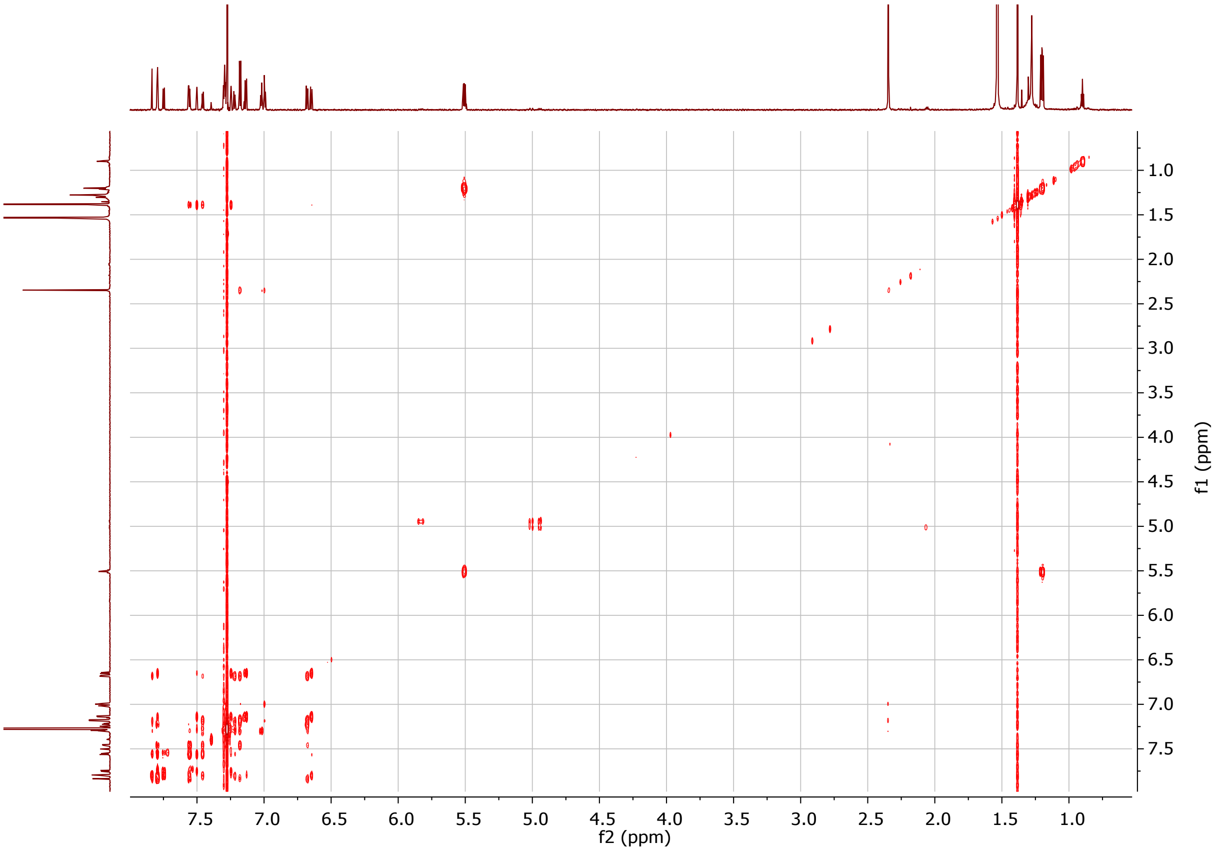


**Fig. S8.** Long-range COSY spectrum of (-)-(*M*,*R*,*R*)-**2** Measured on an 850 MHz spectrometer in $CDCl_3$.

## Computations

### Methods

Geometry optimization of the studied compound and *J*-coupling calculations were performed in Gaussian09 program[10] using B3LYP functional[11-12] and 6-31g(d), 6-311+g(d,p), 6-311++g(2df,2pd) and IGLO-III basis sets.

The indirect spin-spin couplings at the coupled clusters singles and doubles level were calculated with CFOUR program package suitable for performing high-level quantum chemical calculations on atoms and molecules.[13-14]

The calculations of the coupling deformation density (CDD) used for the visualisation of NMR spin-spin coupling pathways and their analysis were done with a modified version of the deMon-KS program.[15] In these calculations we employed the Perdew86 exchange-correlation functional (PP86)[16-19] and IGLO-II basis set.[20] Visualization has been done with AVOGADRO 1.1.1.[21]

The initial plots of the coupling deformation density were done for the medium size model *ii*. For the analysis of separate contributions we used a model structure (*iii*) obtained by removing some parts from the initial one and closing the broken bonds by hydrogens. The geometry of the remaining fragment was kept the same as in the initial structure.

For the analysis of CDD we used the localized molecular orbitals obtained with the Pipek-Mezey localization.[22] We localized all occupied orbitals and the lowest 100 (twice the number of the valence orbitals) unoccupied. Expansion of CDD in terms of molecular orbitals is described in ref.[23]

### Results

Table S1. DFT calculated and experimental *J*-couplings (Hz) in models of compound (-)-(*M*,*R*,R)-**3**. Geometry optimisation of model A at B3LYP/6-31g** level, NMR calculation at B3LYP/IGLO-III level. Models B–G were cut from the geometry optimised model-A and hydrogen atoms were added to the dangling bonds.

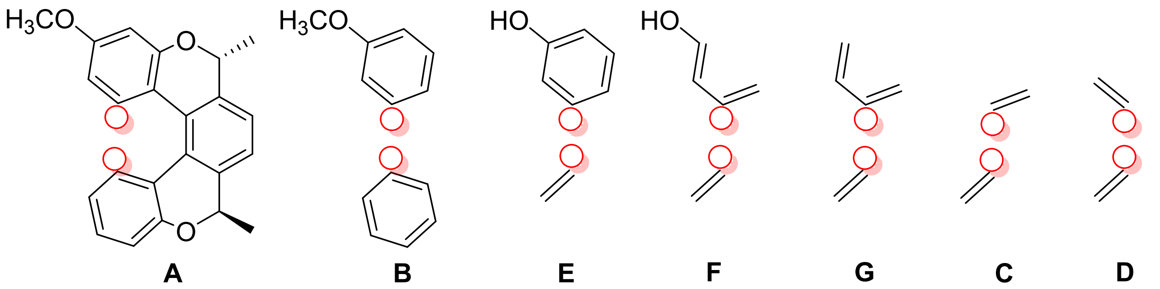


| Coupling | model-A | model-B | model-E | model-F | model-G | model-C | model-D | Experiment |
|---|---|---|---|---|---|---|---|---|
| TSC 1–1’ | 0.62 | 0.62 | 0.59 | 0.69 | 0.70 | 0.66 | 0.68 | 0.61 |
| FC 1–1’ | 0.56 | 0.56 | 0.56 | 0.62 | 0.62 | 0.61 | 0.68 | |
| 1–2 | 8.48 | 8.59 | | | | | | 8.73 |
| 1–4 | 0.36 | 0.43 | | | | | | 0.33 |
| 1’–2’ | 7.64 | 7.79 | | | | | | 7.88 |
| 1’–3’ | 1.42 | 1.20 | | | | | | 1.66 |
| 1’–4’ | 0.45 | 0.62 | | | | | | 0.51 |

Table S2. Fermi contact contribution to TSC calculated at B3LYP and CCSD levels with various basis sets for model D of compound (-)-(*M*,*R*,R)-**3**.

| Basis set | # of basis functions | B3LYP | CCSD |
|---|---|---|---|
| cc-pVDZ | 96 | 0.347 | 0.202 |
| aug-cc-pVDZ | 164 | 0.622 | |
| cc-pVTZ | 232 | 0.739 | |
| aug-cc-pVTZ | 368 | 0.632 | |
| cc-pVQZ | 460 | 0.684 | |
| aug-cc-pVQZ | 688 | 0.701 | |
| ccJ-pVDZ | 180 | 0.796 | 0.542 |
| ccJ-pVTZ | 356 | 0.794 | |
| ccJ-pVQZ | 588 | 0.835 | |
| pcJ-2 | 396 | 0.818 | |
| pcJ-3 | 684 | 0.820 | |
| IGLO-III | 220 | 0.676 | 0.496 |

Attempting to verify the TSC nature of the spin-spin couplings under study, we performed the QTAIM analysis[24] for model D of compound (-)-(*M*,*R*,*R*)-**3** and of model *iv* (see below) of compound (-)-(*M*,*R*,*R*)-**2**. We have found a BCP in the space between the molecular fragments, though not exactly on the line connecting the interacting protons. However, we would like to note that the QTAIM analysis provides information about the ground state electron density in the absence of magnetic perturbations. The analysis of CDD indicates that for the spin-spin couplings under study a significant part of the coupling pathway is provided by the response of the electron density on the nuclear magnetic moments that is not taken into account in the QTAIM approach. Therefore the found BPC may not provide the complete explanation.

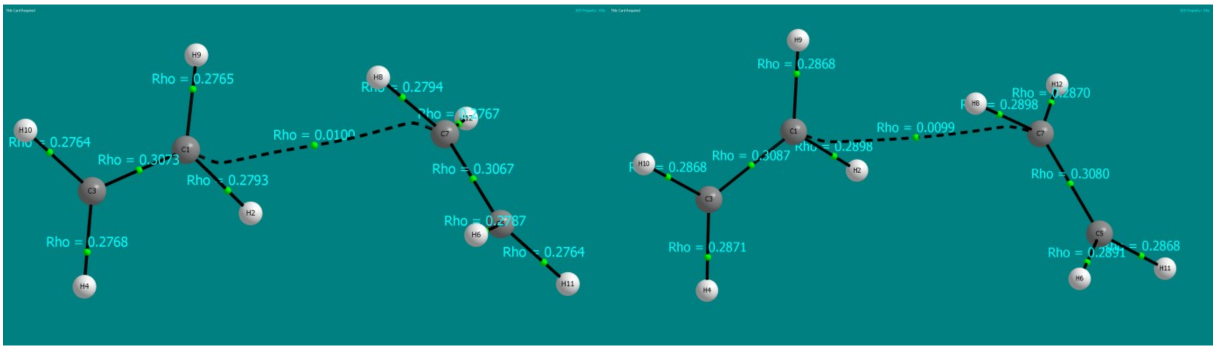


**Fig. S9.** The QTAIM analysis of model D of compound (-)-(*M*,*R*,R)-**3** at B3LYP/6-31g(d) (left) and B3LYP/IGLO-III (right) level. Bond critical points are shown as green spheres together with electron densities. The analysis was performed with the AIMAll program.[25]

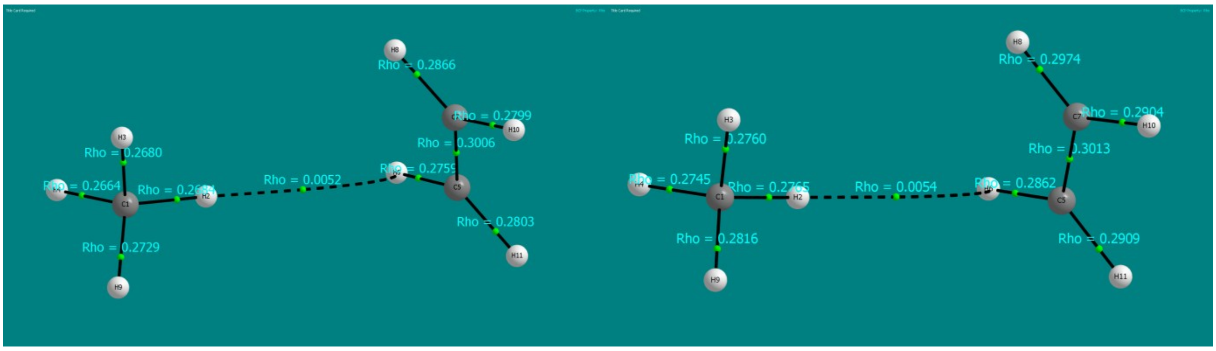


**Fig. S10.** The QTAIM analysis of model *iv* of compound (-)-(*M*,*R*,*R*)-**2** at B3LYP/6-31g(d) (left) and B3LYP/IGLO-III (right) level. Bond critical points are shown as green spheres together with electron densities. The analysis was performed with the AIMAll program.[25]

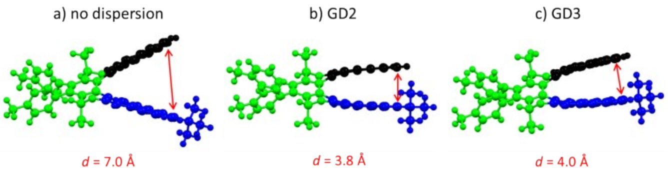


**Fig. S11.** Geometry-optimised (B3LYP/6-31g(d)) structures of compound (-)-(*M*,*R*,*R*)-**2** with (a) no correction for dispersion interactions, (b) Grimme's empirical correction D2,[26] and (c) Grimme's empirical correction D3.[27] The parameter *d* is the distance between the carbon atoms A9 and B8.

Table S3. Dependence of distance *d* in geometry optimised structures on the computational method.

| Functional | Basis set | Dispersion | Solvation | d / Å |
|---|---|---|---|---|
| B3LYP | 6-31g(d) | – | – | 7.028 |
| B3LYP | 6-31g(d) | GD2 | – | 3.782 |
| B3LYP | 6-31g(d) | GD3 | – | 4.024 |
| B3LYP | cc-pVDZ | – | – | 7.096 |
| B3LYP | cc-pVDZ | – | CPCM, chloroform | 7.087 |
| B97D | cc-pVDZ | included | – | 3.767 |
| B97D | cc-pVDZ | included | CPCM, chloroform | 4.186 |

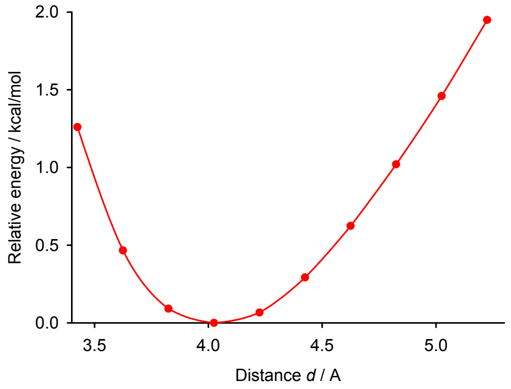


**Fig. S12.** The relative energy of (-)-(*M*,*R*,*R*)-**2** for a given distance *d* between the carbon atoms A9 and B8. The geometry was optimised by DFT (B3LYP/6-31g(d)) employing an empirical dispersion correction term D3.

Table S4. The calculated *J*-coupling values (B3LYP/6-31g(d)) of hydrogen atoms B9 and B11 with the hydrogen atoms of the *tert*-butyl group for the three geometry models: full molecule (-)-(*M*,*R*,*R*)-**2** (*i*), two pyrene molecules without (*ii*) and with (*ii*-opt) optimisation of the positions of the hydrogen atoms added to carbons with dangling bonds, and *tert*-butylbenzene with naphthalene (*iii*). The coupling values of all nine *tert*-butyl hydrogens were averaged.

| | *i* | *ii* | *ii*-opt | *iii* |
|---|---|---|---|---|
| B9 | 0.226 | 0.220 | 0.220 | 0.197 |
| B11 | 0.255 | 0.248 | 0.248 | 0.223 |

Table S5. The basis set dependence of the calculated *J*-coupling values and their components (B3LYP) of hydrogen atoms B9 and B11 with the closest hydrogen atom of the *tert*-butyl group for the smallest geometry model (*iii*).

| | | 6-31g(d) | 6-311+g(d,p) | 6-311++g(2df,2pd) | IGLO-III |
|---|---|---|---|---|---|
| $J^{FC}$ | B9 | -0.312 | -0.255 | -0.254 | -0.257 |
| | B11 | -0.036 | -0.007 | -0.006 | -0.002 |
| $J^{SD}$ | B9 | -0.018 | -0.021 | -0.021 | -0.020 |
| | B11 | -0.035 | -0.035 | -0.031 | -0.036 |
| $J^{PSO}$ | B9 | -2.029 | -2.947 | -3.152 | -3.439 |
| | B11 | -1.669 | -2.283 | -2.436 | -2.688 |
| $J^{DSO}$ | B9 | 3.487 | 3.516 | 3.544 | 3.540 |
| | B11 | 2.723 | 2.735 | 2.750 | 2.747 |
| $J^{PSO}+J^{DSO}$ | B9 | 1.458 | 0.569 | 0.391 | 0.101 |
| | B11 | 1.054 | 0.452 | 0.314 | 0.059 |
| $J^{Total}$ | B9 | 1.128 | 0.293 | 0.116 | -0.176 |
| | B11 | 0.984 | 0.411 | 0.277 | 0.021 |

Table S6. Fermi contact contribution to TSC calculated at B3LYP and CCSD levels with various basis sets for model *iv* of compound (-)-(*M*,*R*,R)-**2**.

| Basis set | # of basis functions | B3LYP | CCSD |
|---|---|---|---|
| cc-pVDZ | 82 | –0.151 | –0.251 |
| aug-cc-pVDZ | 141 | –0.207 | |
| cc-pVTZ | 202 | –0.223 | –0.155 |
| aug-cc-pVTZ | 322 | –0.214 | |
| cc-pVQZ | 405 | –0.191 | |
| aug-cc-pVQZ | 608 | –0.196 | |
| ccJ-pVDZ | 155 | –0.277 | –0.208 |
| ccJ-pVTZ | 315 | –0.216 | |
| ccJ-pVQZ | 521 | –0.236 | |
| pcJ-2 | 345 | –0.224 | |
| pcJ-3 | 599 | –0.226 | |
| IGLO-III | 185 | –0.154 | –0.151 |

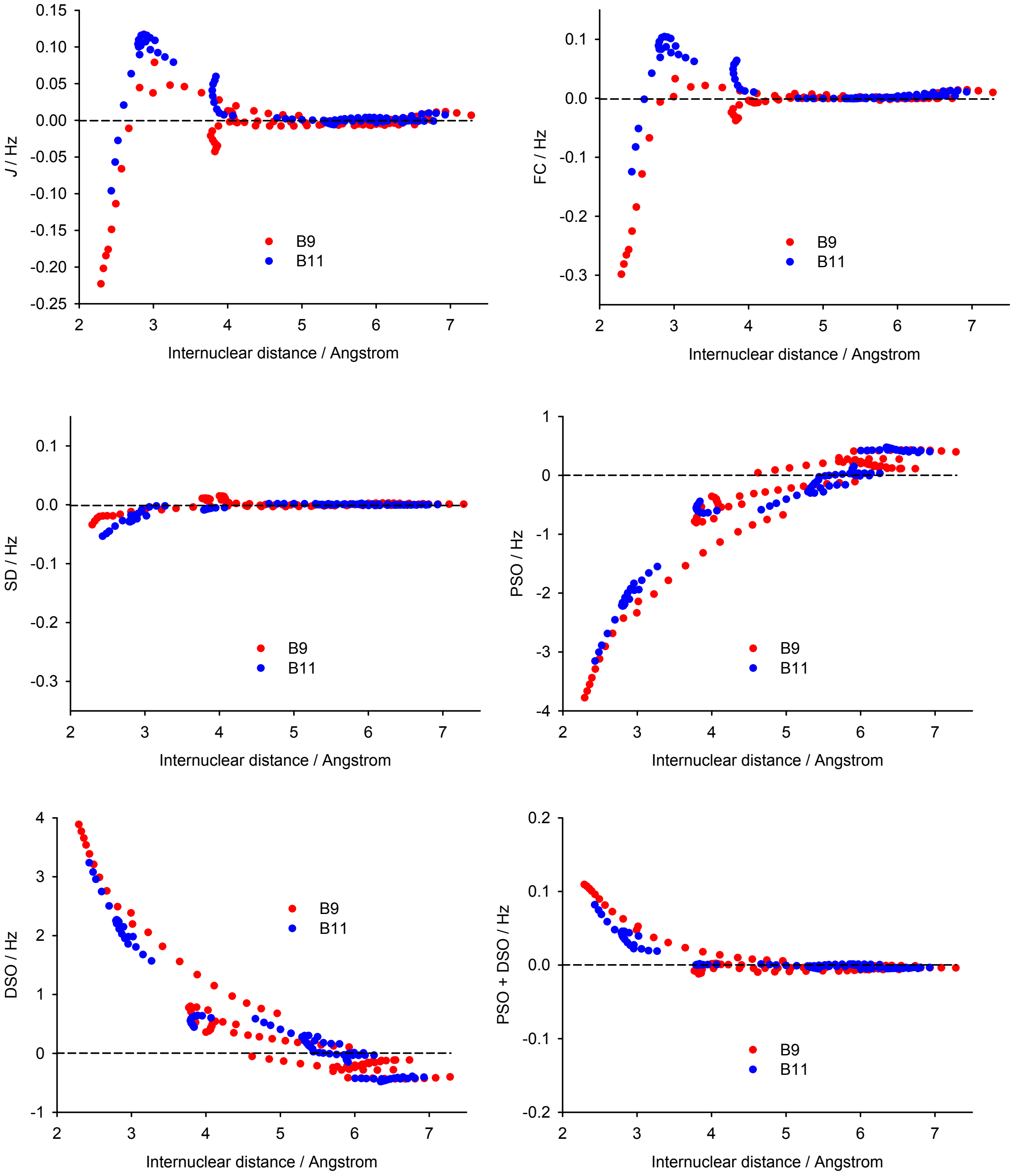


**Fig. S13.** The distance dependence of the calculated (the smallest geometry model *iii*, B3LYP/IGLO-III) through-space indirect coupling and its components (Fermi contact – FC, Spin dipolar – SD, Paramagnetic spin-orbit – PSO, Diamagnetic spin-orbit – DSO) for all individual pairs of the hydrogen atoms of the *tert*-butyl group with hydrogen atoms B9 and B11. The couplings were calculated for 10 geometries differing in the distance *d* (3.4–5.2 Å) between the carbon atoms A9 and B8 in (-)-(*M*,*R*,*R*)-**2**.

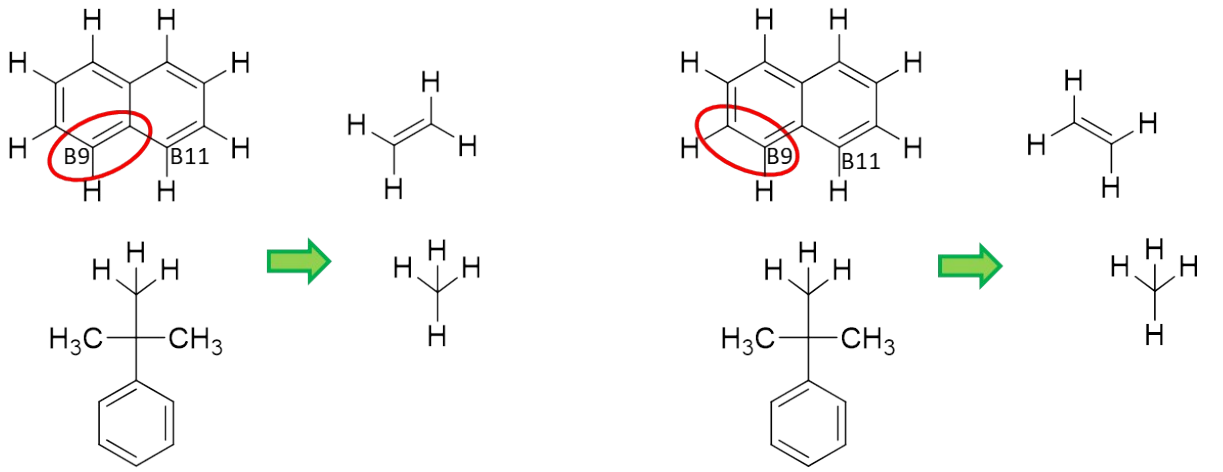


**Fig. S14.** The structure of very small models of compound (-)-(*M*,*R*,*R*)-**2** used for CCSD calculations of Fermi contact terms: model *iv* (left) and model *v* (right).

Table S7. The Fermi contact term calculated at DFT(B3LYP) level for models *iii*–*v* and at CCSD level for models *iv*–*v*.

| Model *iii* | Model *iv* | | Model *v* | |
|---|---|---|---|---|
| DFT | DFT | CCSD | DFT | CCSD |
| -0.197 | -0.154 | -0.151 | -0.149 | -0.140 |
| -0.025 | -0.026 | -0.028 | -0.030 | -0.035 |
| -0.005 | -0.0001 | -0.006 | -0.033 | 0.009 |

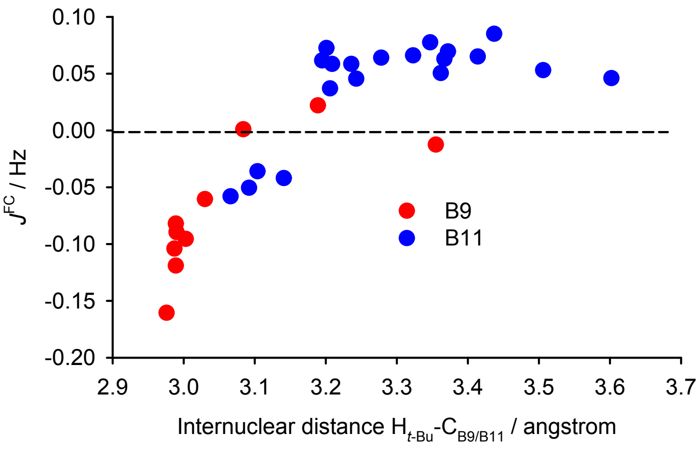


**Fig. S15.** Dependence of the FC contribution to $J(H_{t\text{-Bu}}\text{-}H_{B9/B11})$ on the distance between the *tert*-butyl hydrogen and the B9/B11 carbon. The plot shows the results for the *tert*-butyl hydrogens with $R(H_{t\text{-Bu}}–C_{B9/B11}) < 3.7$ Å in the small model *iii* (PP86).

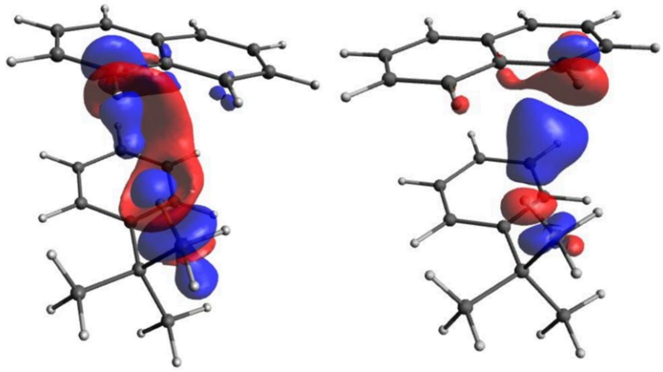

**Fig. S16.**The coupling pathway for $J$($H_{t\text{-Bu}}$–$H_{B9}$) (left) and for $J$($H_{t\text{-Bu}}$–$H_{B11}$) (right) for the small model *iii* of (-)-(*M*,*R*,*R*)-**2***.* The isosurface value is 0.00003 and 0.00006 a.u., respectively.

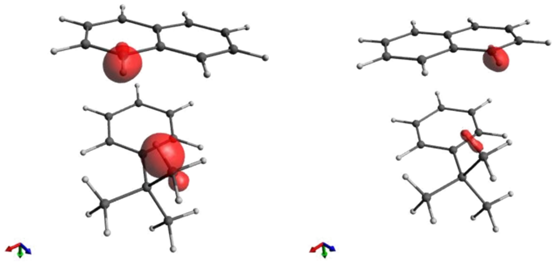

**Fig. S17.** The contribution from the occupied-occupied block of the CDD matrix to the coupling pathway for $J$($H_{t\text{-Bu}}$–$H_{B9}$) and for $J$($H_{t\text{-Bu}}$–$H_{B11}$) in the small model *iii* of (-)-(*M*,*R*,*R*)-**2**. The isosurface value is 0.00003 a.u.

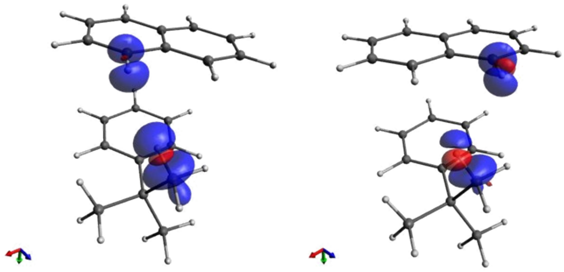

**Fig. S18.** The contribution from the vacant-vacant block of the CDD matrix to the coupling pathway for $J$($H_{t\text{-Bu}}$–$H_{B9}$) and for $J$($H_{t\text{-Bu}}$–$H_{B11}$) in the small model *iii* of (-)-(*M*,*R*,*R*)-**2**. The isosurface value is 0.00003 a.u.

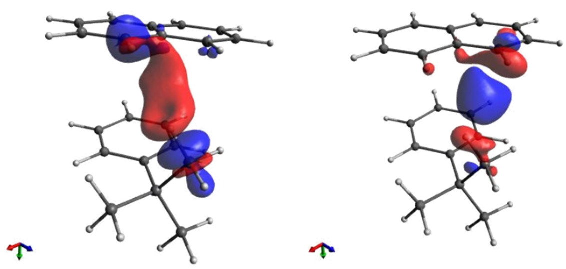

**Fig. S19.** The contribution from the occupied-vacant block of the CDD matrix to the coupling pathway for $J$($H_{t\text{-Bu}}$–$H_{B9}$) and for $J$($H_{t\text{-Bu}}$–$H_{B11}$) in the small model *iii* of (-)-(*M*,*R*,*R*)-**2**. The isosurface value is 0.00003 and 0.00006 a.u., respectively.

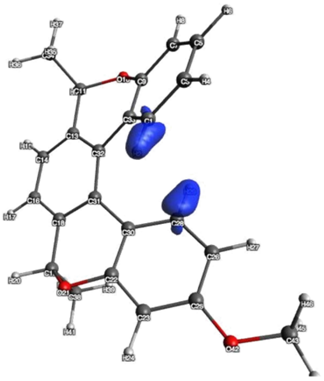

**Fig. S20.** The contribution from the occupied-occupied block of the CDD matrix to the coupling pathway for *J*(H1–H1') in compound (-)-(*M*,*R*,R)-**3**. The isosurface value is 0.00008 a.u.

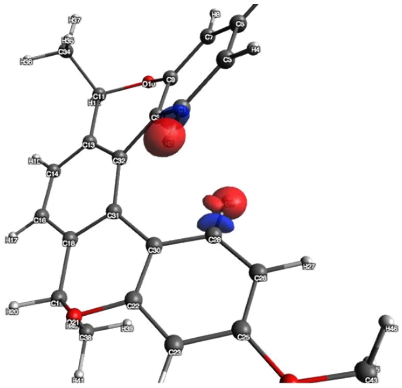

**Fig. S21.** The contribution from the vacant-vacant block of the CDD matrix to the coupling pathway for *J*(H1–H1') in compound (-)-(*M*,*R*,R)-**3**. The isosurface value is 0.00008 a.u.

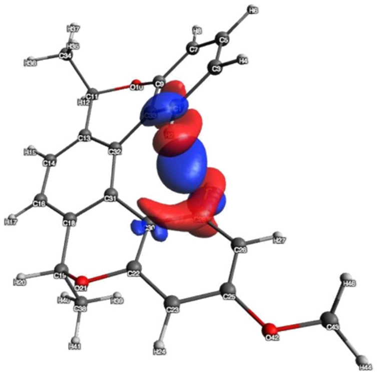

**Fig. S22.** The contribution from the occupied-vacant block of the CDD matrix to the coupling pathway for *J*(H1–H1') in compound (-)-(*M*,*R*,R)-**3**. The isosurface value is 0.00008.

The LMO pairs have been separated into three main groups: occupied-occupied, vacant-vacant and occupied-vacant. The contributions from the first two groups for both couplings in (-)-(*M*,*R*,*R*)-**2** are shown in Figs. S15 and S16. The contribution from the occupied-occupied block of the CDD matrix comes mainly from the two orbitals, C–$H_{t\text{-Bu}}$ and C–$H_{B9/B11}$ (that is their squares and the cross-term) as it is clearly seen on Fig. S15. This contribution is less pronounced for proton B11 as it should be expected since the distance between $H_{t\text{-Bu}}$ and $H_{B11}$ is longer (2.6 Å) than for $H_{B9}$ (2.49 Å). However, overall the occupied-occupied block gives similar contributions in both cases just as the vacant-

vacant block (see Fig. S16). The effect of occupied-occupied and vacant-vacant blocks on CDD in the "through-space" area between the A- and B-planes is minor.

In contrast, the occupied-vacant block (see Fig. S17) contributes to the through-space part for both couplings giving very different plots. Obviously, these plots reflect the asymmetry in the position of the $H_{t\text{-Bu}}$ proton with respect to $H_{B9}$ and $H_{B11}$ and therefore with respect to the response of the π-system B on the magnetic interaction of nucleus $H_{t\text{-Bu}}$ with $H_{B9}$ and $H_{B11}$, respectively. However, if one restricts the occupied-vacant block to the combination of only four orbitals directly related to $H_{t\text{-Bu}}$ and $H_{B9/B11}$, that is to the occupied LMOs representing the C–$H_{t\text{-Bu}}$ and C–$H_{B9/B11}$ bonds and their two unoccupied antibonding orbitals, then the resulting plots would be again very similar (see Fig. 8 in the manuscript).

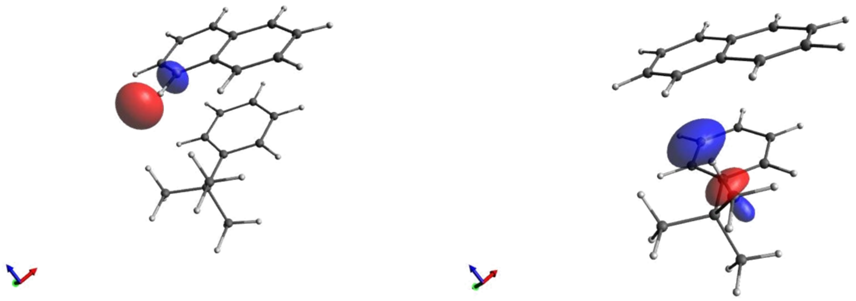

**Fig. S23.** σ*( C-$H_{B9/11}$) (left) and σ*(C-$H_{t\text{-Bu}}$) (right) in the small model *iii* of (-)-(*M*,*R*,*R*)-**2**. The isosurface value is 0.1 a.u.

**Overlap of LMO densities**

The overlap integral between two molecular orbitals is zero due to their orthogonality. Therefore, as a measure of the overlap between two LMOs we use the overlap of their densities defined as follows (ref.[23]):

$$\Omega = \iint \varphi_i^2(\vec{r}_1)\delta(\vec{r}_1 - \vec{r}_2)\varphi_j^2(\vec{r}_2)dV_1dV_2 = \int \varphi_i^2(\vec{r})\varphi_j^2(\vec{r})dV$$

Here $\varphi_i$ and $\varphi_j$ are localized molecular orbitals. If we consider two electrons occupying molecular orbitals $\varphi_i$ and $\varphi_j$ as independent particles, the integrand represents the probability of finding them in the same element of volume.

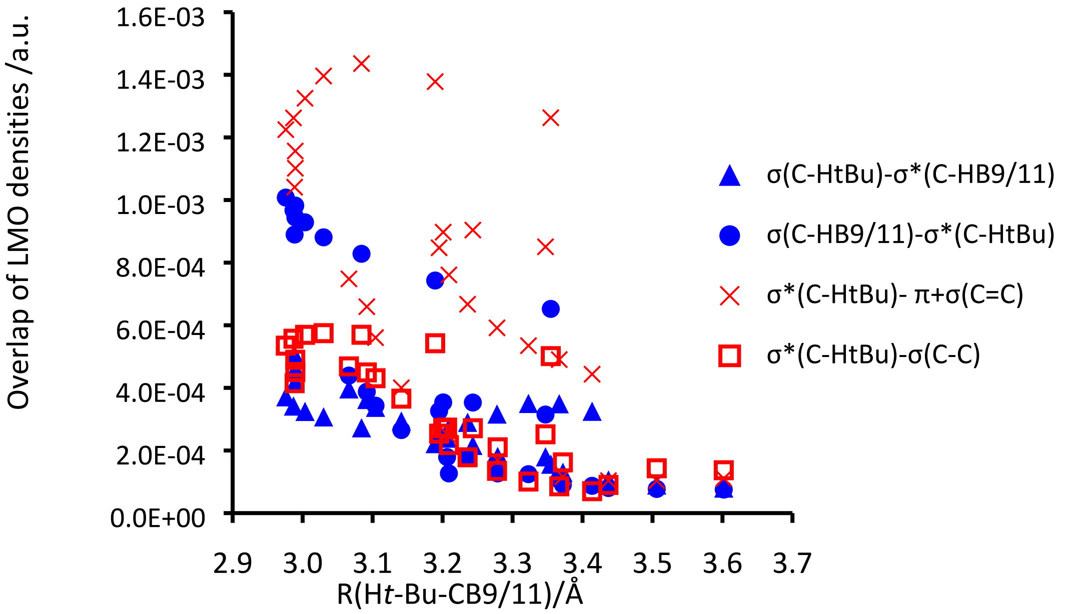


**Fig. S24**. Overlap of densities for selected LMO pairs in (-)-(*M*,*R*,*R*)-**2** as the function of the distance between $H_{t\text{-Bu}}$ and $C_{B9/11}$. The pairs contributing to the primary pathway are marked blue and to the secondary pathway – red.

Among the LMO pairs contributing to the primary pathway in (-)-(*M*,*R*,*R*)-**2**, the largest overlap is provided by C–$H_{B9/11}$ - σ*(C-$H_{t\text{-Bu}}$) (full blue circles in Fig. S22). The next one is by C-$H_{t\text{-Bu}}$ - σ*(C-$H_{B9/11}$) (full blue triangles). Both overlaps became smaller for larger distances. However, the dependences are not monotonous because the overlap depends not only on the distance but also on the mutual orientation of the involved orbitals and the latter was not taken into account.

Pairs contributing to the secondary pathway are marked red. The largest overlap comes from the antibonding σ*(C–$H_{t\text{-Bu}}$) with $C_{B9/11}$–C single and double bonds. All three are almost of similar magnitude; the overlap with σ(C=C) is slightly larger than with π(C=C). In order to make the plot less crowded, we combined the data for σ(C=C) and π(C=C) and show the overlap of their combined density with the σ*(C–$H_{t\text{-Bu}}$) density (full red squares).

For distances shorter than 3 Å, the overlap of σ*(C–$H_{t\text{-Bu}}$) with double $C_{B9/11}$=C bonds (full red squares) is larger than the C–$H_{B9/11}$ – σ*(C-$H_{t\text{-Bu}}$) overlap (full blue circles), and the empty red squares showing the σ*(C–$H_{t\text{-Bu}}$) – σ(C–C) overlap are slightly above the blue tringles for C–$H_{t\text{-Bu}}$ – σ*(C–$H_{B9/11}$). Therefore, one can expect that for shorter distances the secondary pathway should be dominating. Since the primary pathway is likely to give a positive contribution to *J*(H–H) (based on its similarity with the pathways for couplings via the overlap of lone pairs) and the *J* values for shorter distances are negative, it is reasonable to assume that the secondary pathway gives a negative contribution. Please note the two full red squares at the medium distances of 3.19 Å and 3.35 Å displaying a larger than average overlap of σ*(C–$H_{t\text{-Bu}}$) with the C=C bond. As a consequence, for the same distances two red circles representing the *J* values for $H_{B9}$ in Fig. 6 in the manuscript and Fig. S13 in ESI drop below the general trend due the negative contribution from the secondary pathway.

When the distance is increasing, the overlaps contributing to the secondary pathway diminish on the average faster than those contributing to the primary pathway, making both pathways

approximately equally important. As a result, the $J$ values for medium distances are close to zero. For distances longer than 3.4 Å all discussed overlaps become smaller and almost equal to each other. The importance of a particular pair of LMOs for the spin-spin coupling pathway is defined not only by their overlap but also by the value of the corresponding element in the CDD matrix, which reflects the sensitivity of this pair of orbitals to the FC interaction. The elements corresponding to C–$H_{B9/11}$ – σ*(C–$H_{t\text{-Bu}}$) and C–$H_{t\text{-Bu}}$ – σ*(C–$H_{B9/11}$) are always larger than the elements contributing to the secondary pathway because for the former both orbitals in each pair include a coupled proton. One of the largest elements of the CDD matrix corresponds to the cross-term between the occupied orbitals representing the C–$H_{t\text{-Bu}}$ and C–$H_{B9/11}$ bonds. However, the importance of this pair for the coupling pathway is relatively low because of their small overlap (see Fig. S15). Therefore, we have to take into account both factors. The elements of the CDD matrix corresponding to the most important LMO pairs are shown in Table S6. The data are obtained for the model structure *iii* employed for visualization of the coupling pathways. They correspond to three couplings with different $H_{t\text{-Bu}}$–$C_{B9/11}$ distances: short (2.976 Å), medium (3.142 Å) and long (3.278 Å). The first two rows show the most important elements for the primary pathway and the last three to the secondary one.

Table S8. Selected elements of the CDD matrix corresponding to the most important LMO pairs for the TSC in (-)-(*M*,*R*,*R*)-**2**.

| LMO pair | R = 2.976 Å | R=3.142 Å | R=3.278 Å |
|---|---|---|---|
| σ*(C–$H_{t\text{-Bu}}$) – C–$H_{B9/11}$ | -0.00560 | 0.00867 | 0.00528 |
| σ*(C–$H_{B9/11}$) – (C–$H_{t\text{-Bu}}$) | 0.00409 | 0.00680 | -0.00430 |
| σ*(C–$H_{t\text{-Bu}}$) – π(C=C) | 0.00097 | -0.00198 | -0.00109 |
| σ*(C–$H_{t\text{-Bu}}$) – σ(C=C) | -0.00010 | -0.00153 | -0.00040 |
| σ*(C–$H_{t\text{-Bu}}$) – ($C_{B9/11}$–C ) | -0.00131 | -0.00142 | -0.00127 |

In the considered examples, all CDD elements for the medium distance are moderately larger than their counterparts for the short distance. For the longest distance, all elements decrease in the absolute values, but the elements corresponding to σ*(C–$H_{t\text{-Bu}}$) – π(C=C) and σ*(C–$H_{t\text{-Bu}}$) – σ(C=C) diminish stronger. These elements correspond to the LMO two pairs mainly responsible for the secondary pathway. Therefore, it is reasonable to say that for long distances the primary pathway is dominating. This explains the positive sign of the couplings for long distances.

**$^1H$ and $^{13}C$ NMR spectra of (-)-(*M*,*R*,*R*)-2, (+)-(*R*)-3, 4, (-)-(*R*)-5, (-)-(*R*,*R*)-7, 10, 11, 13 and (+)-(*R*)-14.**

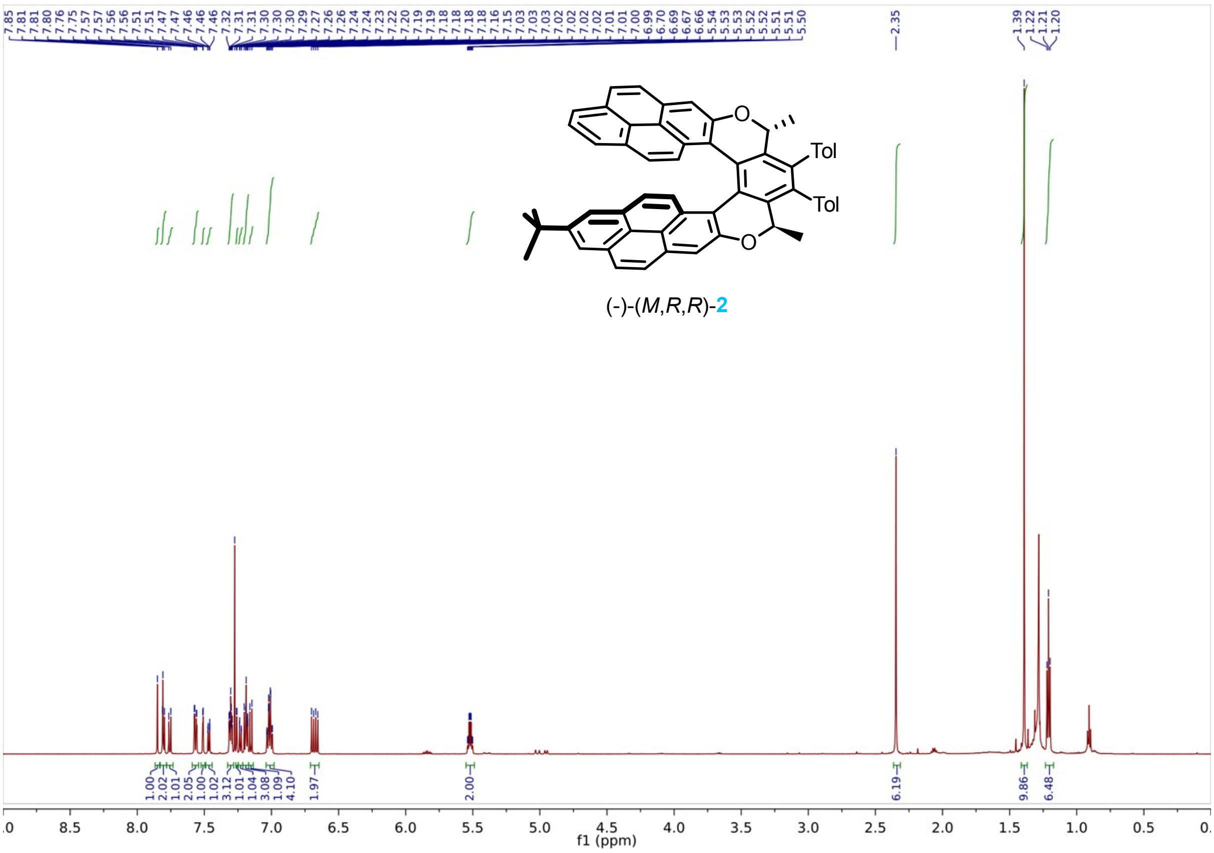


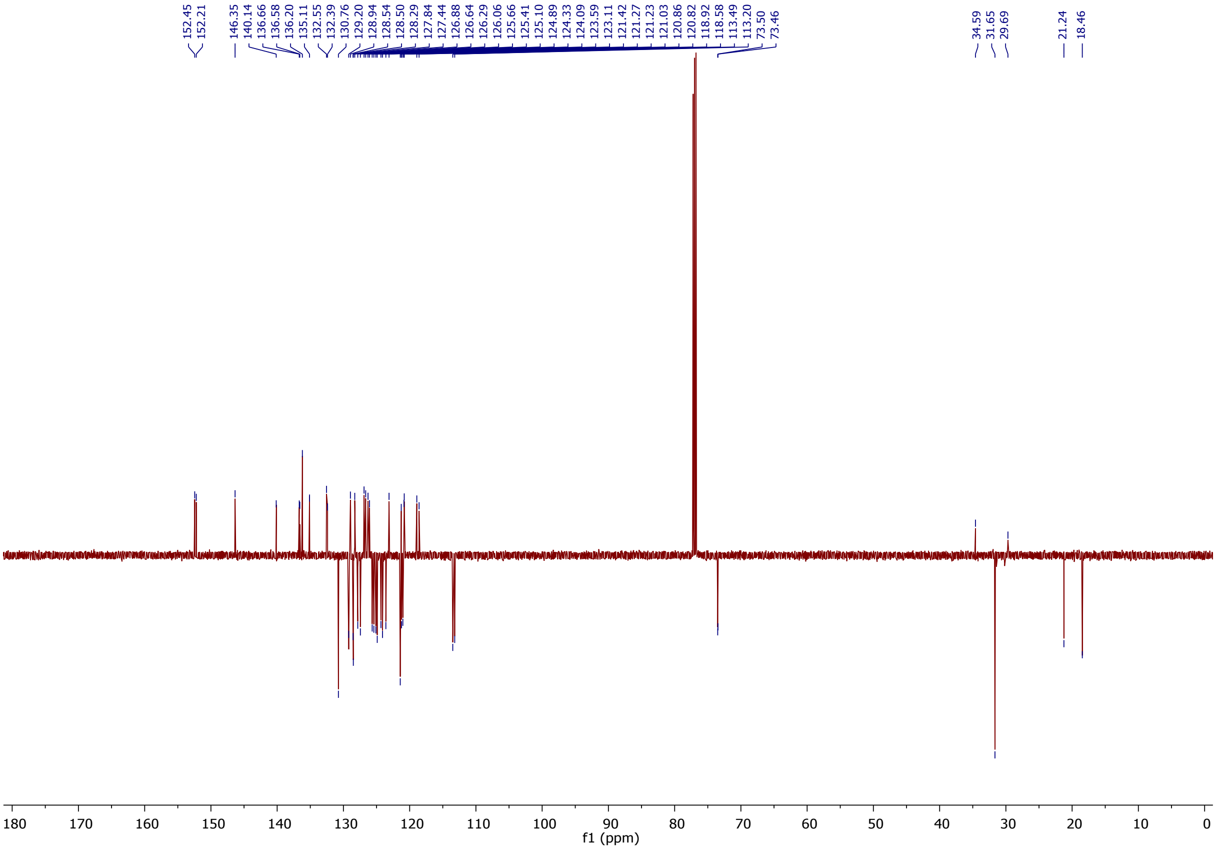

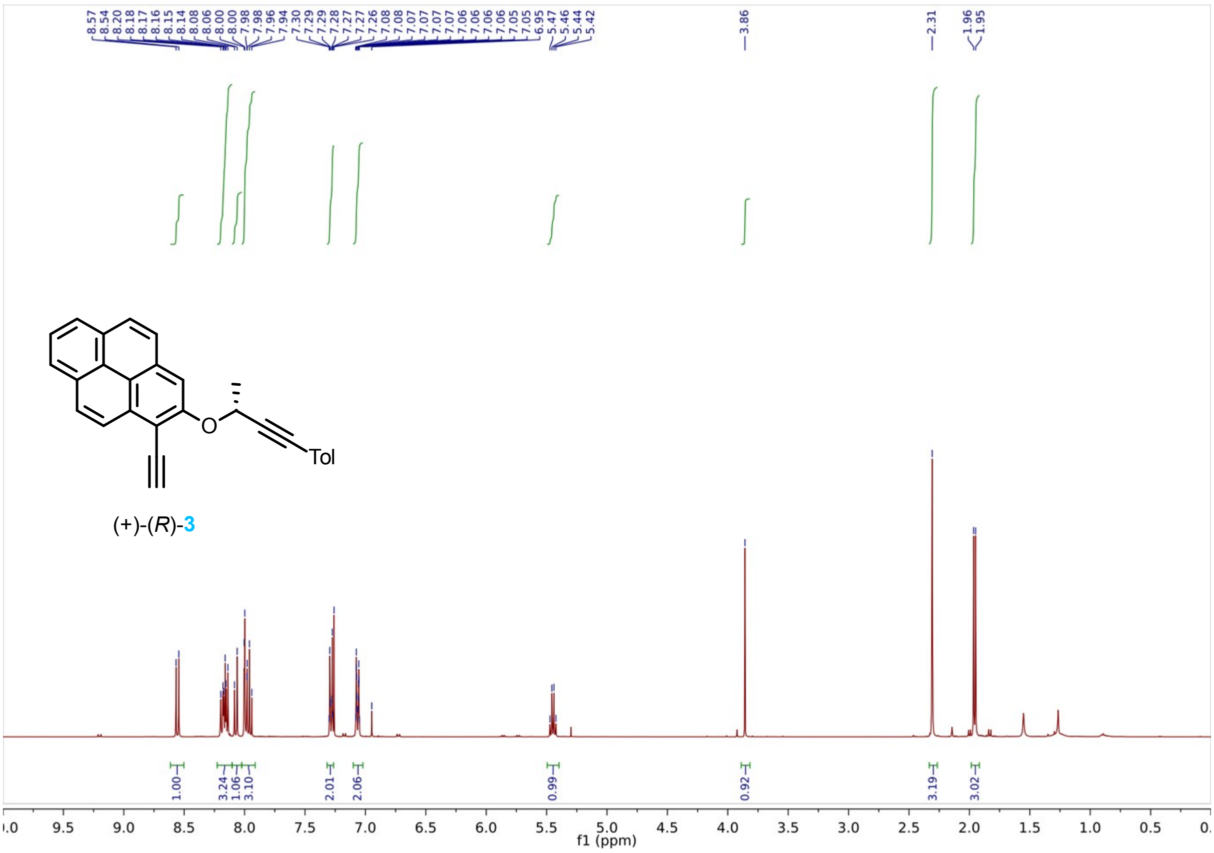
8.57 8.54 8.20 8.18 8.17 8.16 8.15 8.14 8.08 8.06 8.00 7.98 7.98 7.96 7.94 7.30 7.29 7.29 7.28 7.27 7.27 7.26 7.08 7.08 7.07 7.07 7.07 7.07 7.06 7.06 7.06 7.06 7.05 7.05 6.95 5.47 5.46 5.44 5.42
3.86
2.31
1.96 1.95
O
Tol
(+)-(R)-3
1.00 3.24 1.06 3.10 2.01 2.06 0.99 0.92 3.19 3.02
f1 (ppm)

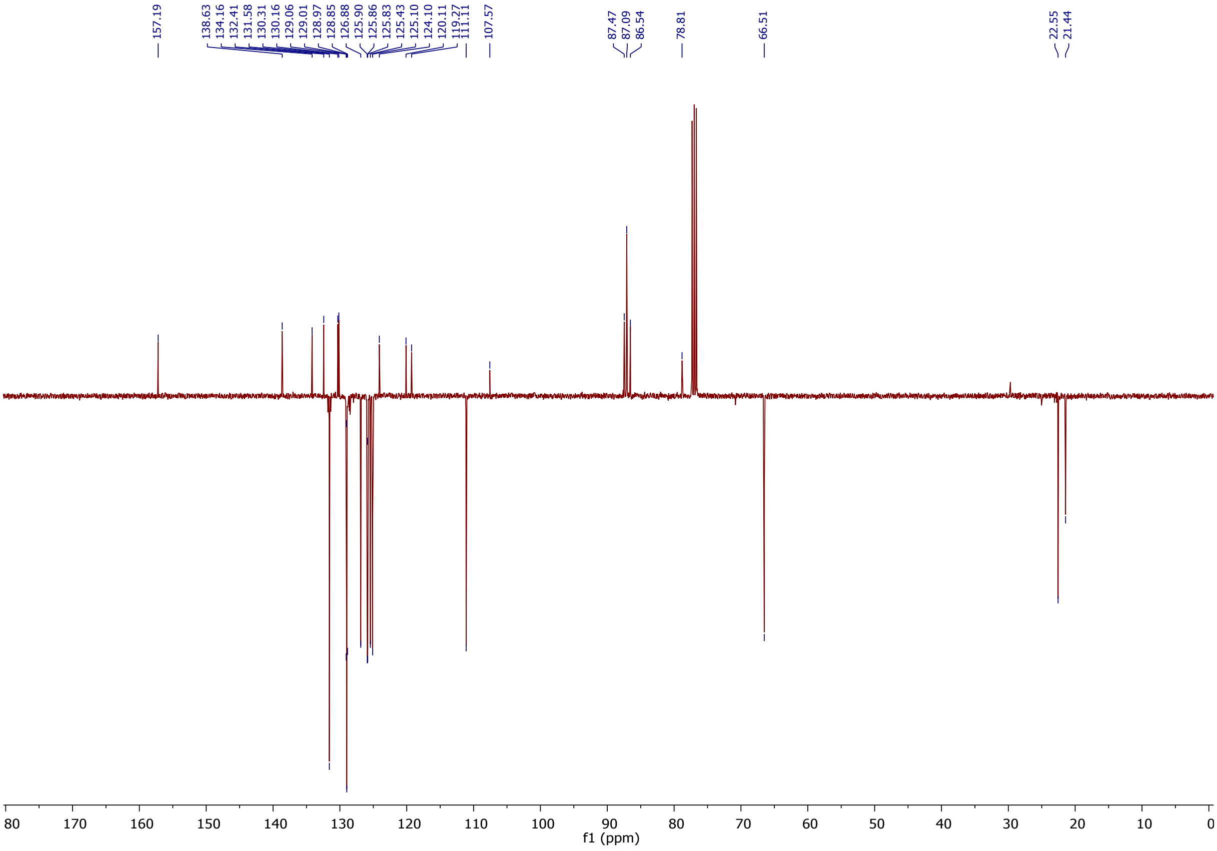
157.19
138.63 134.16 132.41 131.58 130.31 130.16 129.06 129.01 128.97 128.85 126.88 125.90 125.86 125.83 125.43 125.10 124.10 120.11 119.27 111.11
107.57
87.47 87.09 86.54
78.81
66.51
22.55 21.44
f1 (ppm)

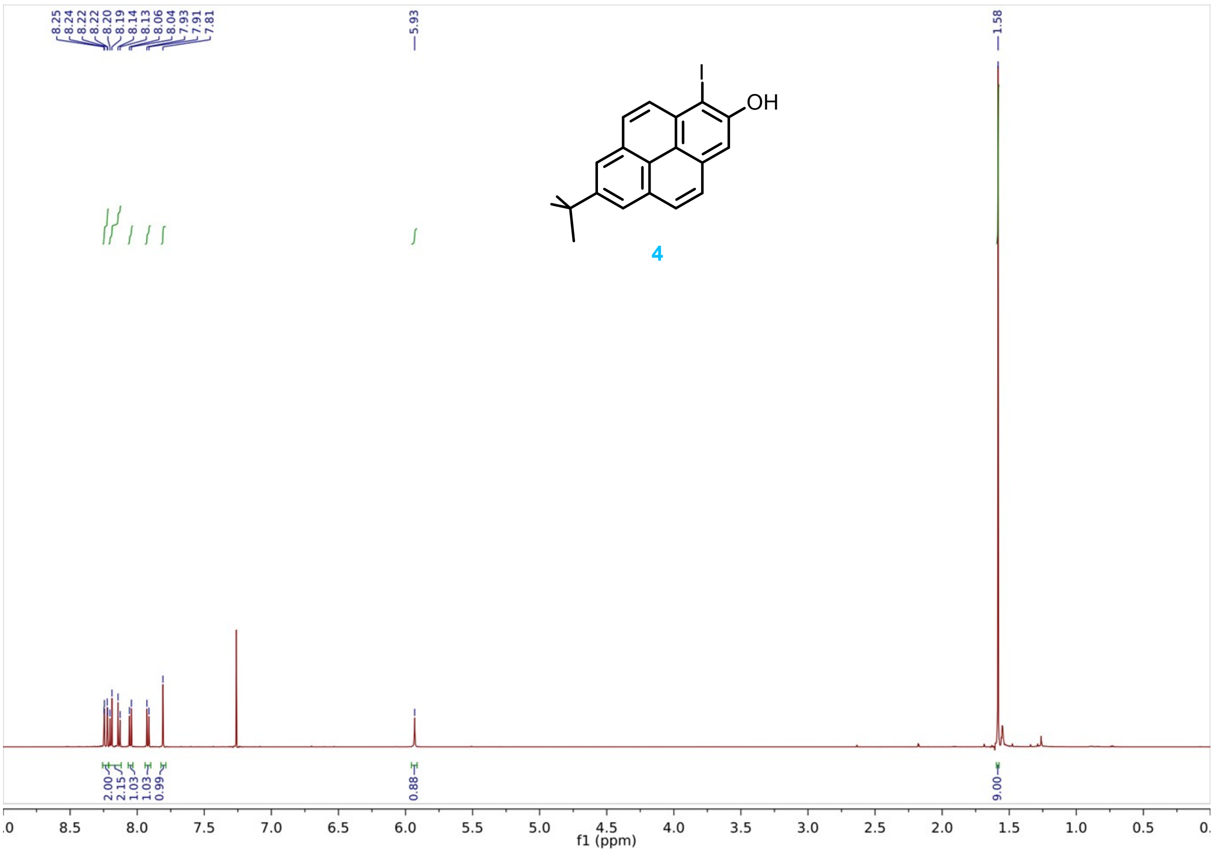
8.25
8.24
8.22
8.22
8.20
8.19
8.14
8.13
8.06
8.04
7.93
7.91
7.81
5.93
1.58
OH
4
2.00
2.15
1.03
1.03
0.99
0.88
9.00
f1 (ppm)

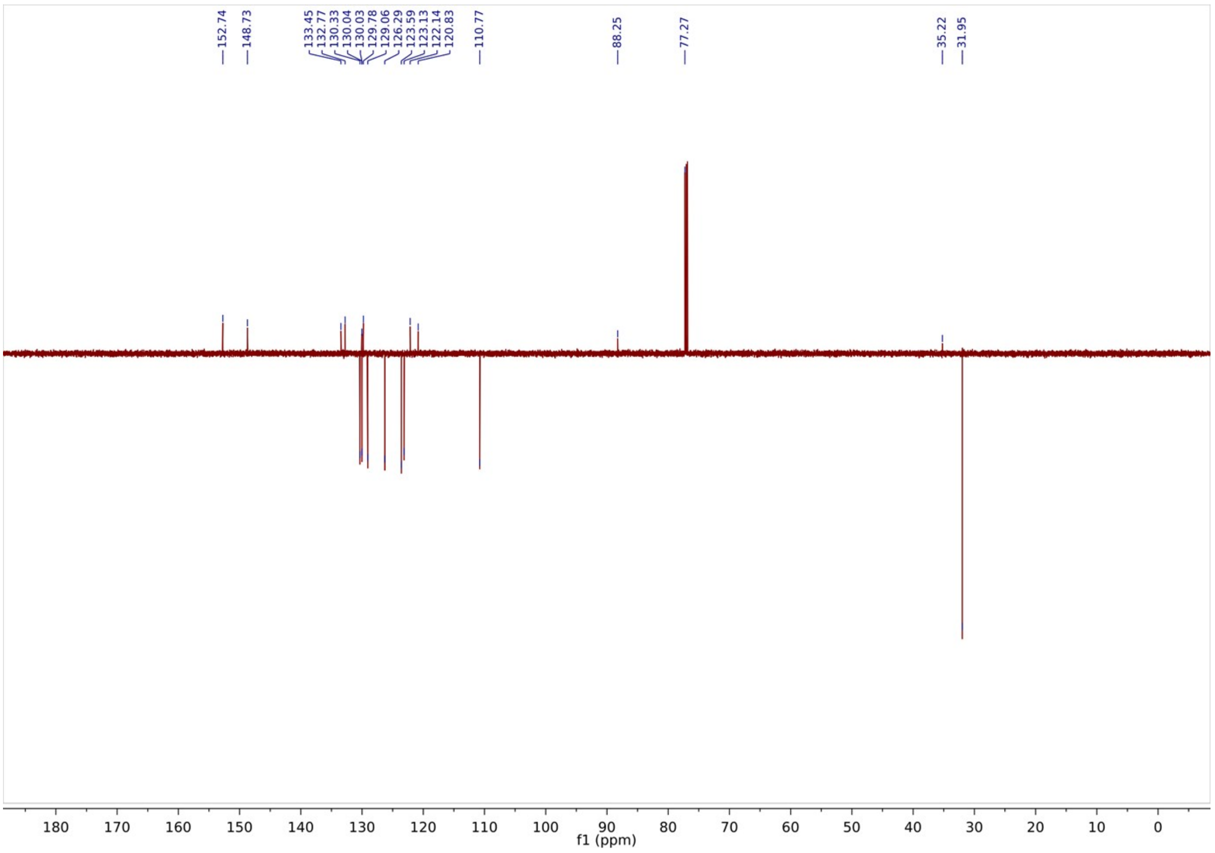
152.74
148.73
133.45
132.77
130.33
130.04
130.03
129.78
129.06
126.29
123.59
123.13
122.14
120.83
110.77
88.25
77.27
35.22
31.95
f1 (ppm)

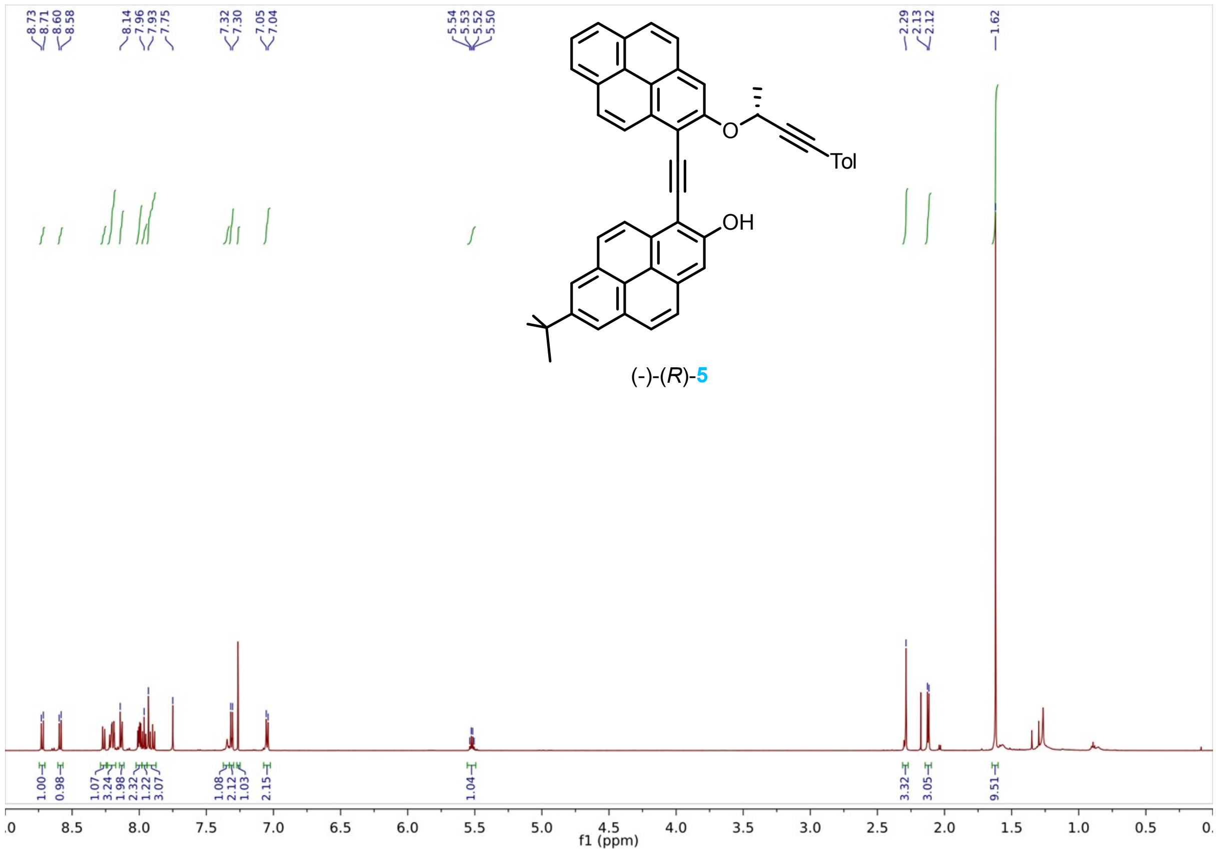

(-)-(*R*)-**5**

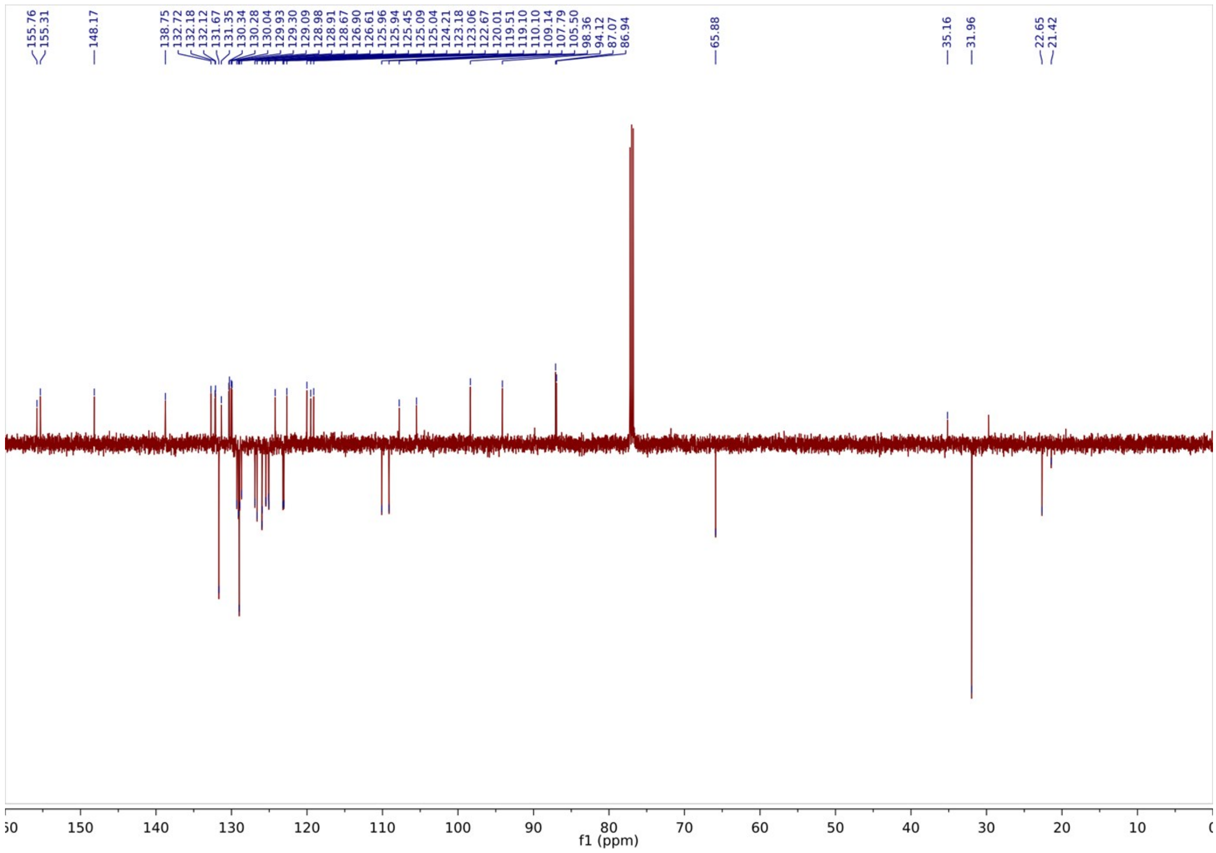

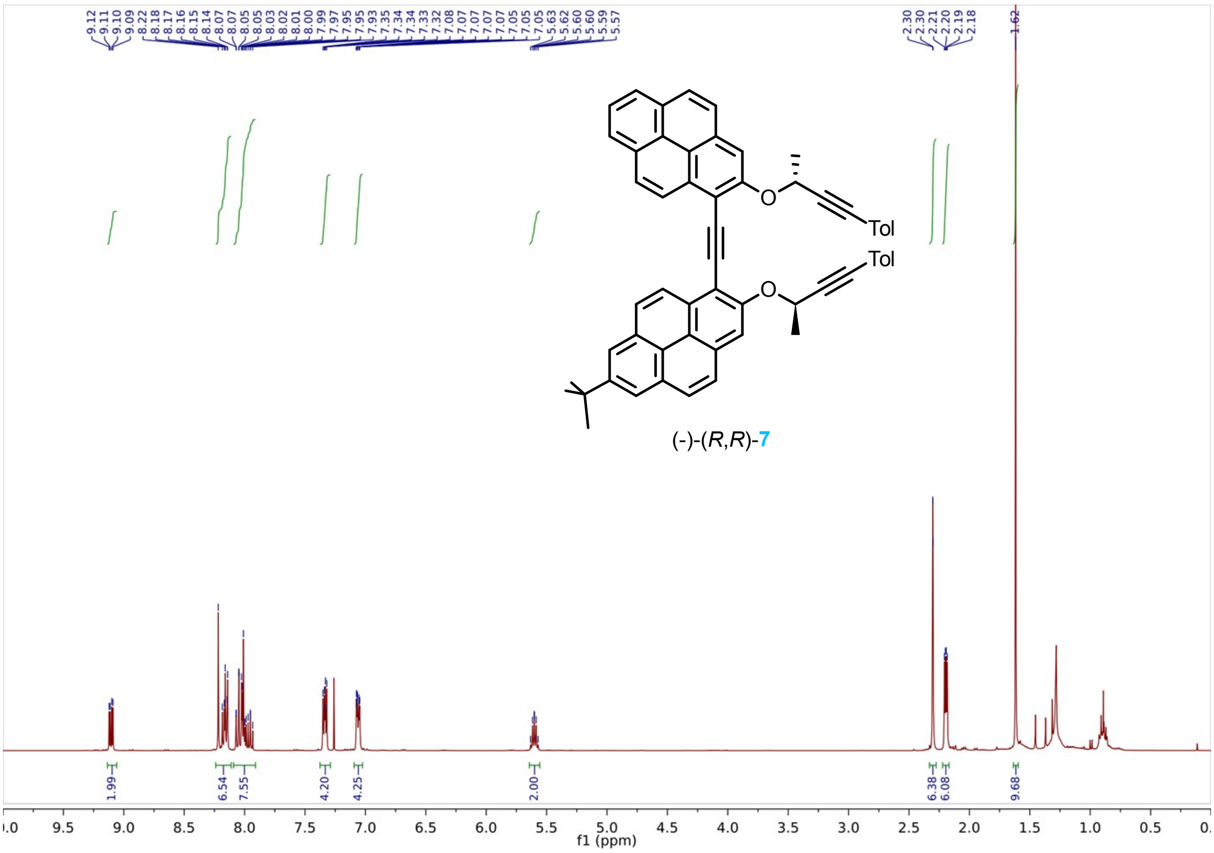
9.12 9.11 9.10 9.09 8.22 8.18 8.17 8.16 8.15 8.14 8.07 8.07 8.05 8.05 8.03 8.02 8.01 8.00 7.99 7.97 7.95 7.95 7.93 7.35 7.34 7.34 7.33 7.32 7.08 7.07 7.07 7.07 7.05 7.05 5.63 5.62 5.60 5.59 5.57
2.30 2.30 2.21 2.20 2.19 2.18
1.62
O
Tol
(-)-(R,R)-7
1.99 6.54 7.55 4.20 4.25 2.00 6.38 6.08 9.68
f1 (ppm)

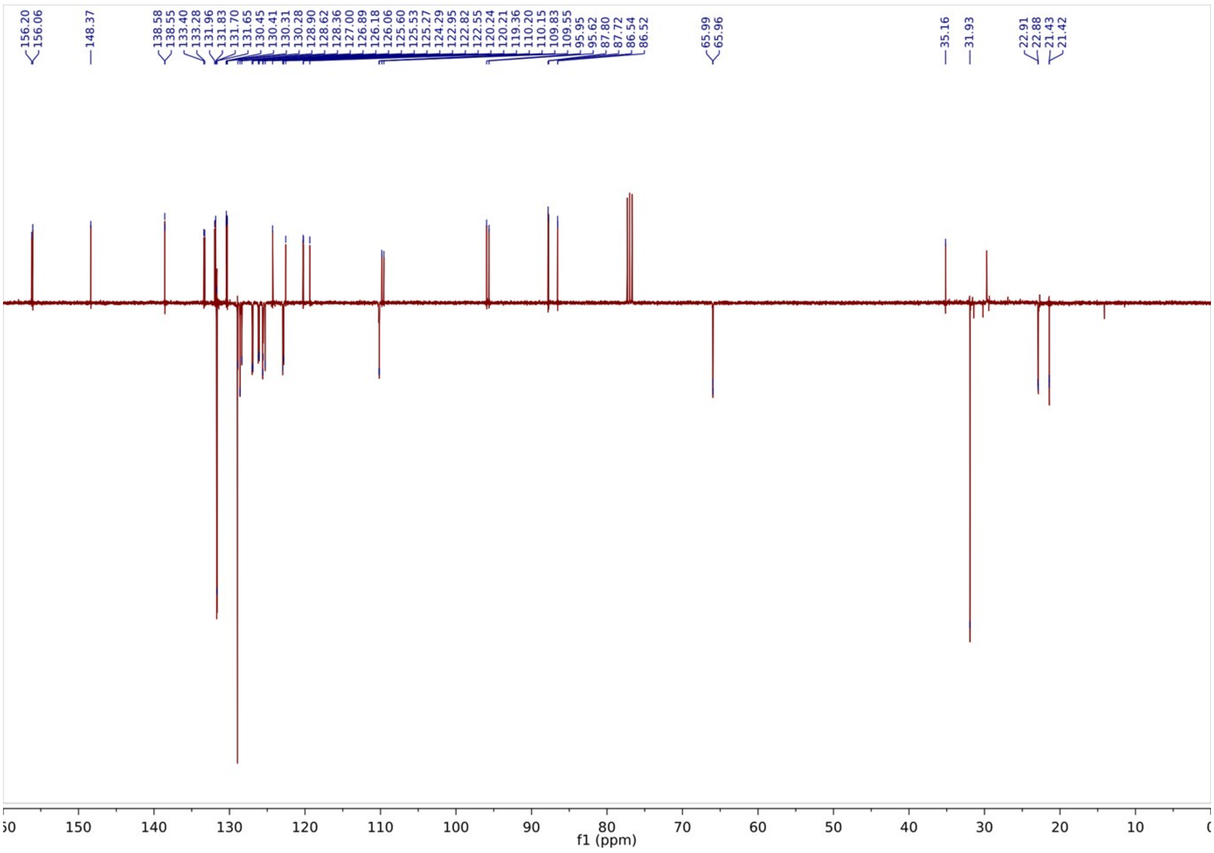
156.20 156.06
148.37
138.58 138.55 133.40 133.28 131.96 131.83 131.70 131.65 130.45 130.41 130.31 130.28 128.90 128.62 128.36 127.00 126.89 126.18 126.06 125.60 125.53 125.27 124.29 122.95 122.82 122.55 120.24 120.21 119.36 110.20 110.15 109.83 109.55 95.95 95.62 87.80 87.72 86.54 86.52
65.99 65.96
35.16
31.93
22.91 22.88 21.43 21.42
f1 (ppm)

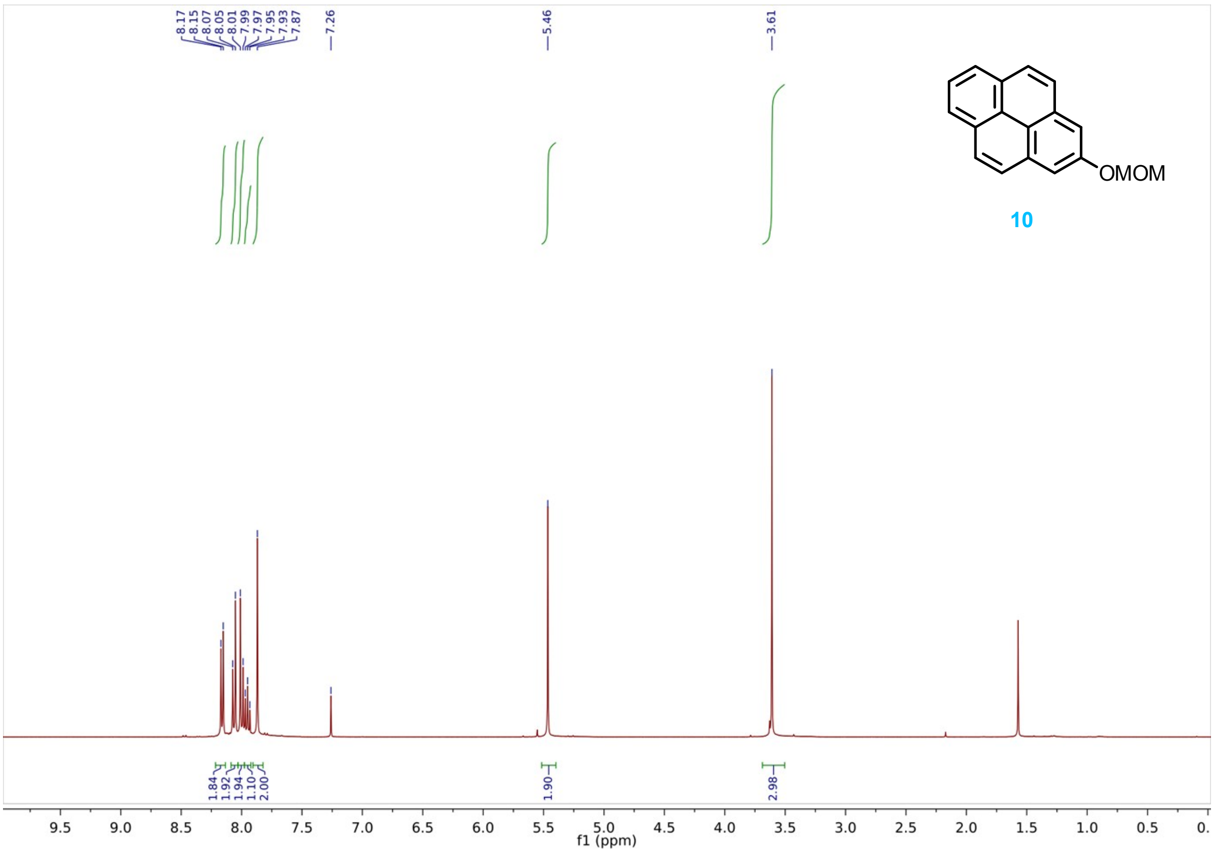
8.17
8.15
8.07
8.05
8.01
7.99
7.97
7.95
7.93
7.87
7.26
5.46
3.61
OMOM
10
1.84
1.92
1.94
1.10
2.00
1.90
2.98
9.5 9.0 8.5 8.0 7.5 7.0 6.5 6.0 5.5 5.0 4.5 4.0 3.5 3.0 2.5 2.0 1.5 1.0 0.5 0.
f1 (ppm)

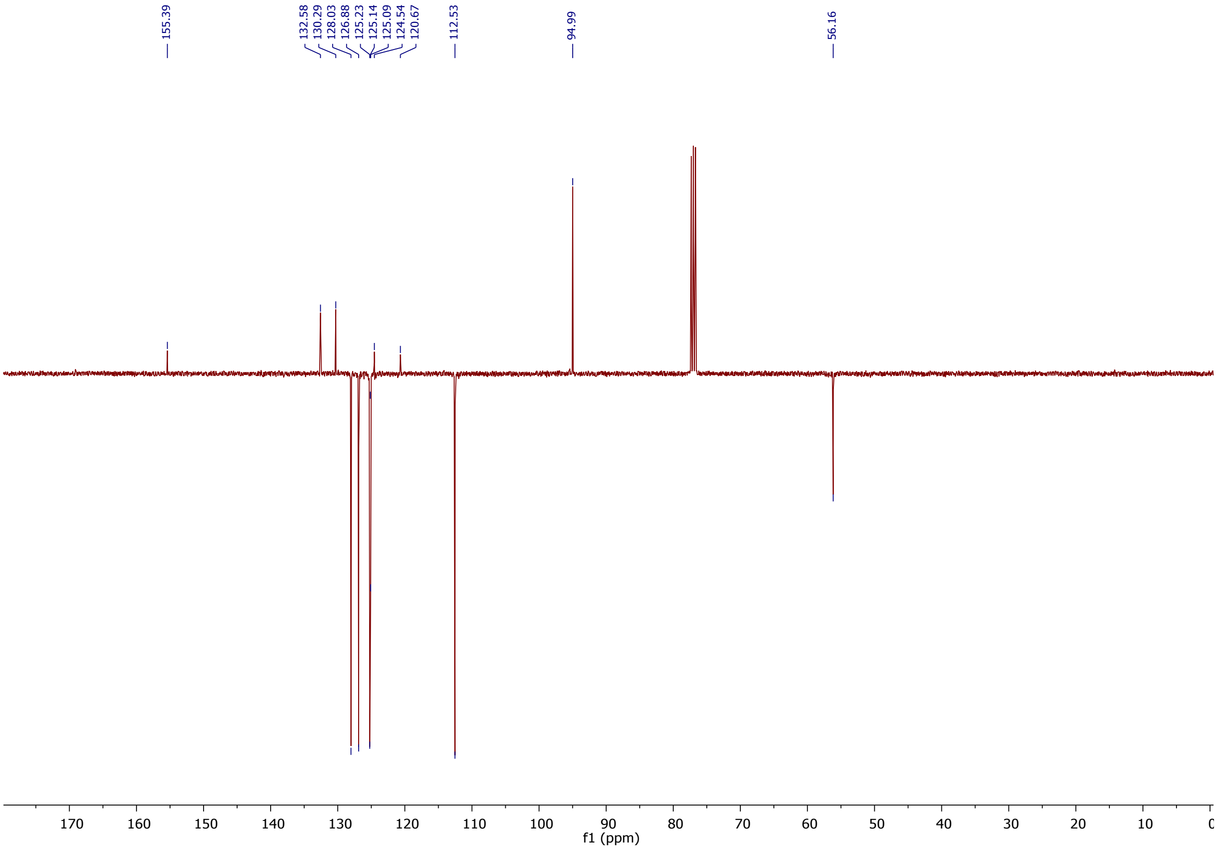
155.39
132.58
130.29
128.03
126.88
125.23
125.14
125.09
124.54
120.67
112.53
94.99
56.16
170 160 150 140 130 120 110 100 90 80 70 60 50 40 30 20 10 0
f1 (ppm)

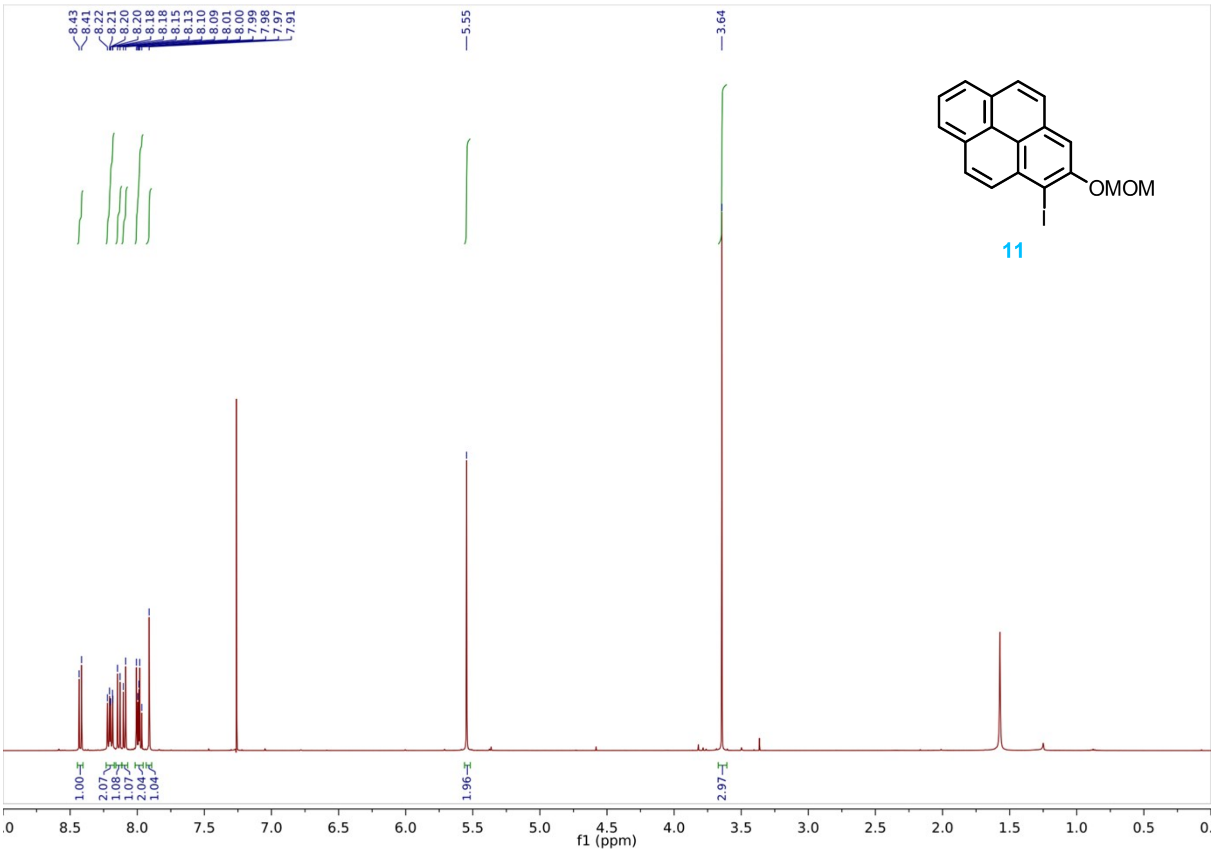
8.43
8.41
8.22
8.21
8.20
8.20
8.18
8.18
8.15
8.13
8.10
8.09
8.01
8.00
7.99
7.98
7.97
7.91
5.55
3.64
OMOM
I
11
1.00
2.07
1.08
1.07
2.04
1.04
1.96
2.97
f1 (ppm)

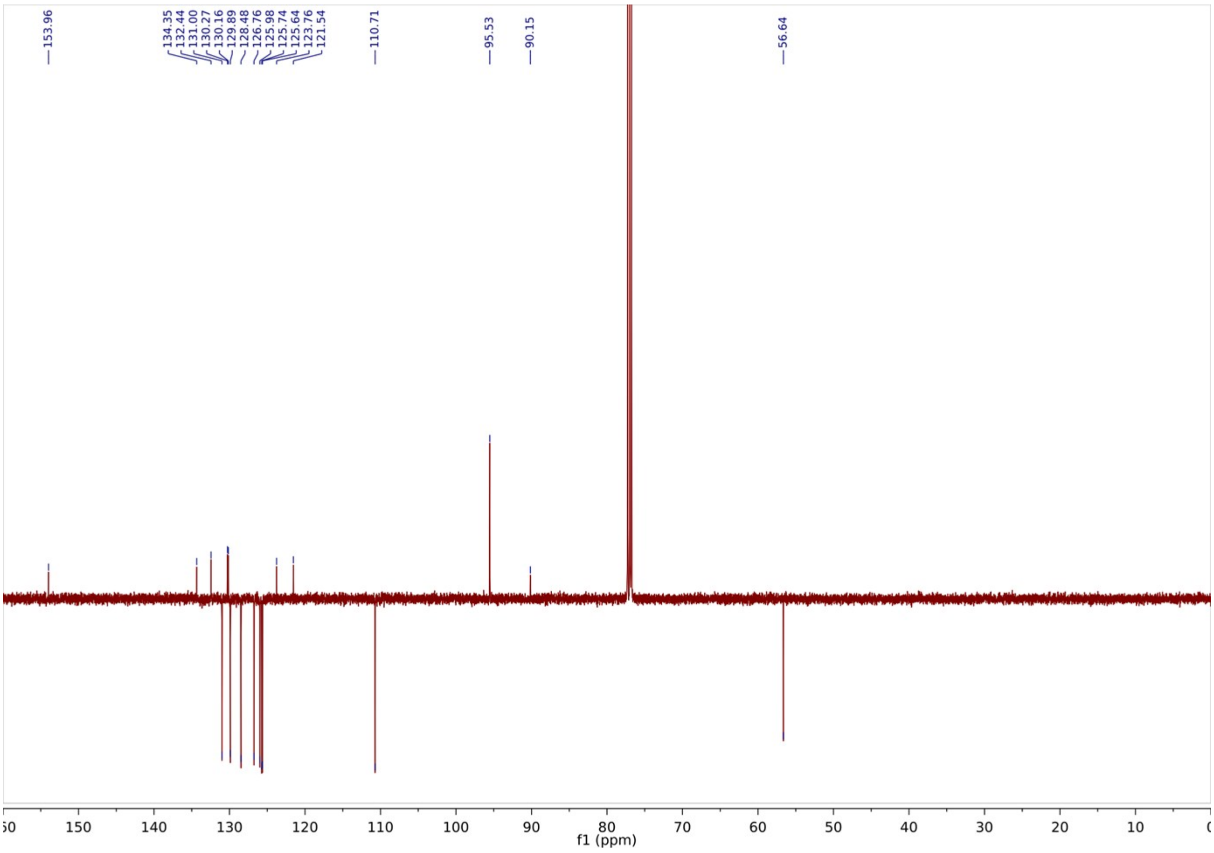
153.96
134.35
132.44
131.00
130.27
130.16
129.89
128.48
126.76
125.98
125.74
125.64
123.76
121.54
110.71
95.53
90.15
56.64
f1 (ppm)

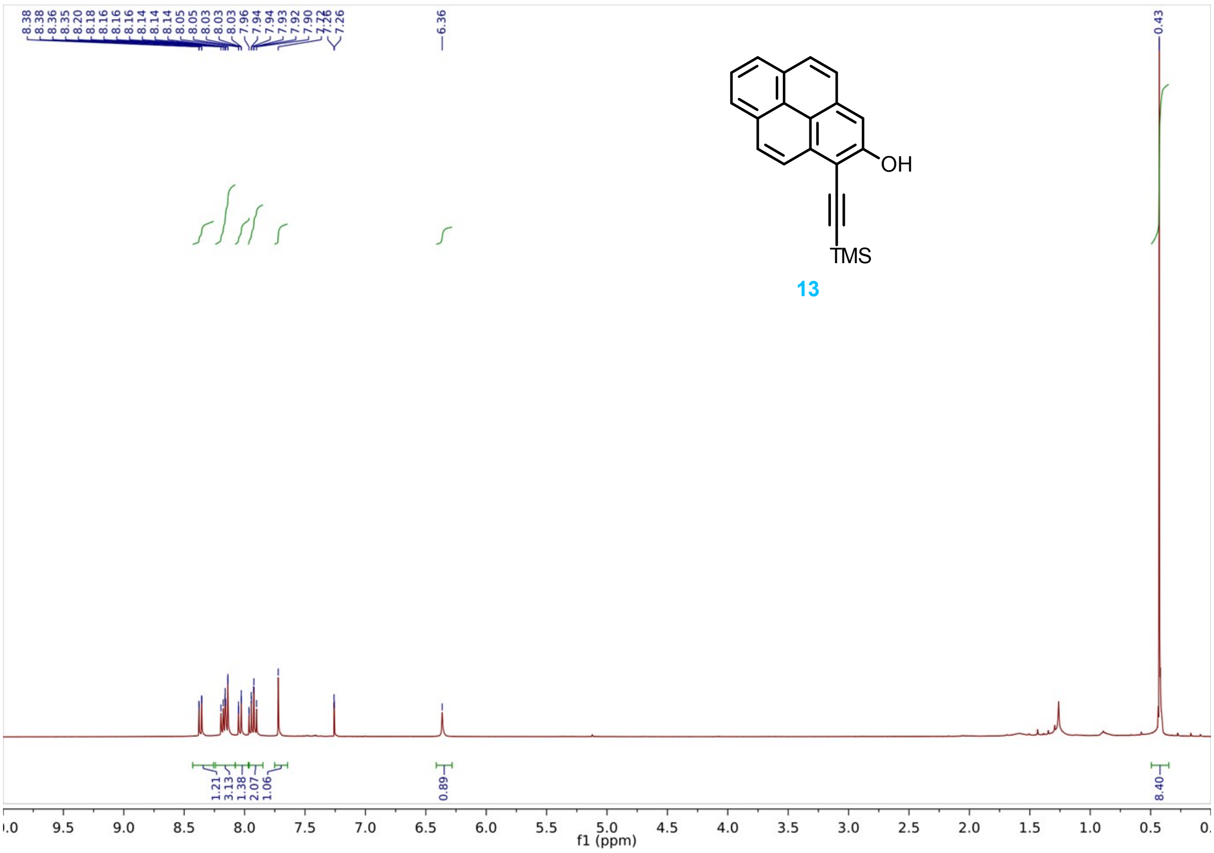

OH
TMS
13
f1 (ppm)


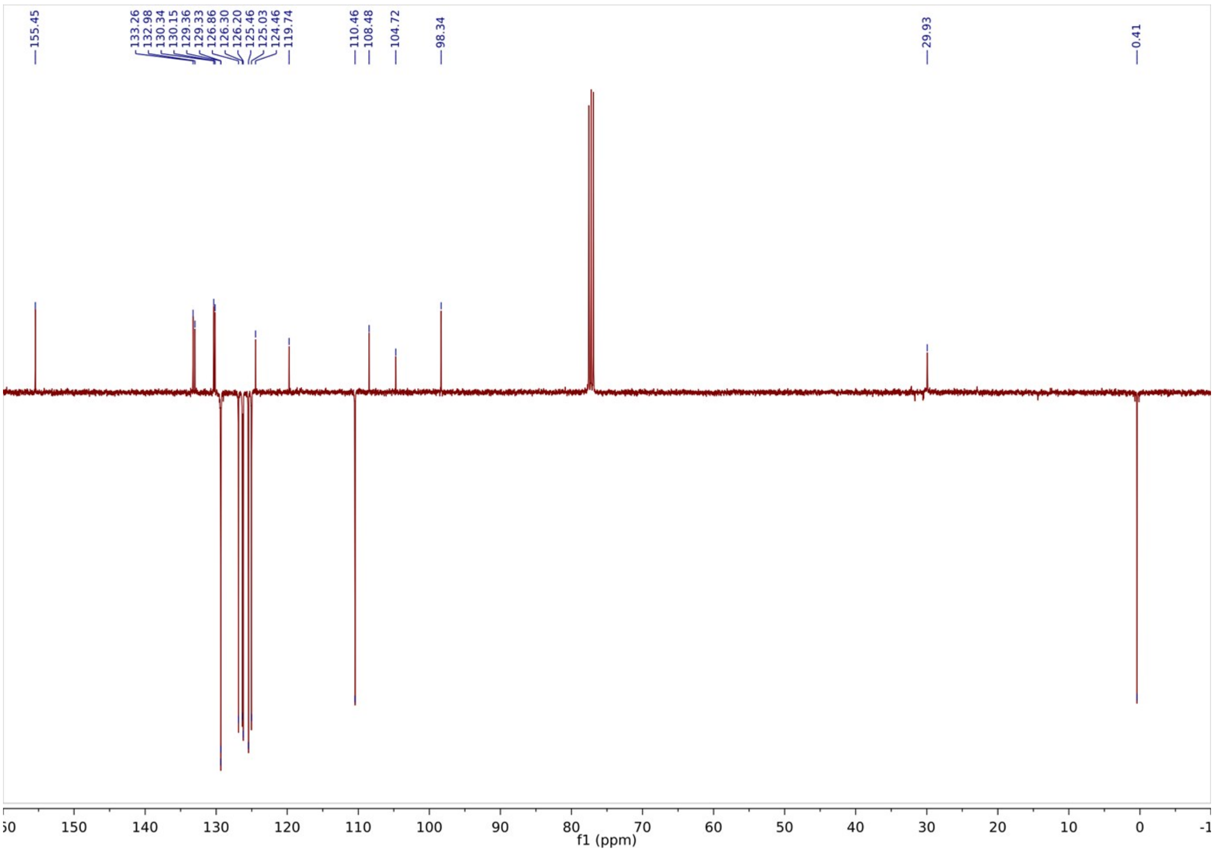

155.45
133.26
132.98
130.34
130.15
129.36
129.33
126.86
126.30
126.20
125.46
125.03
124.46
119.74
110.46
108.48
104.72
98.34
29.93
0.41
f1 (ppm)

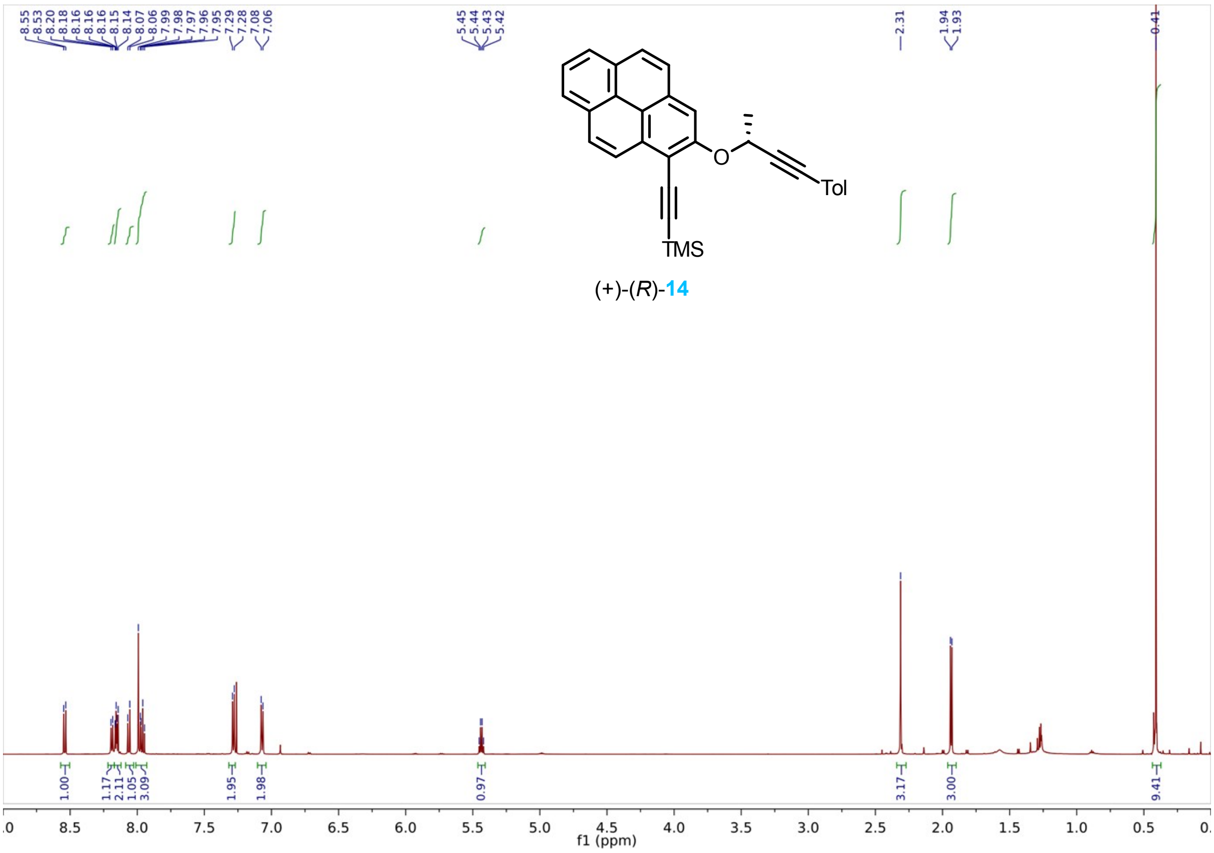
TMS
Tol
(+)-(R)-14
f1 (ppm)

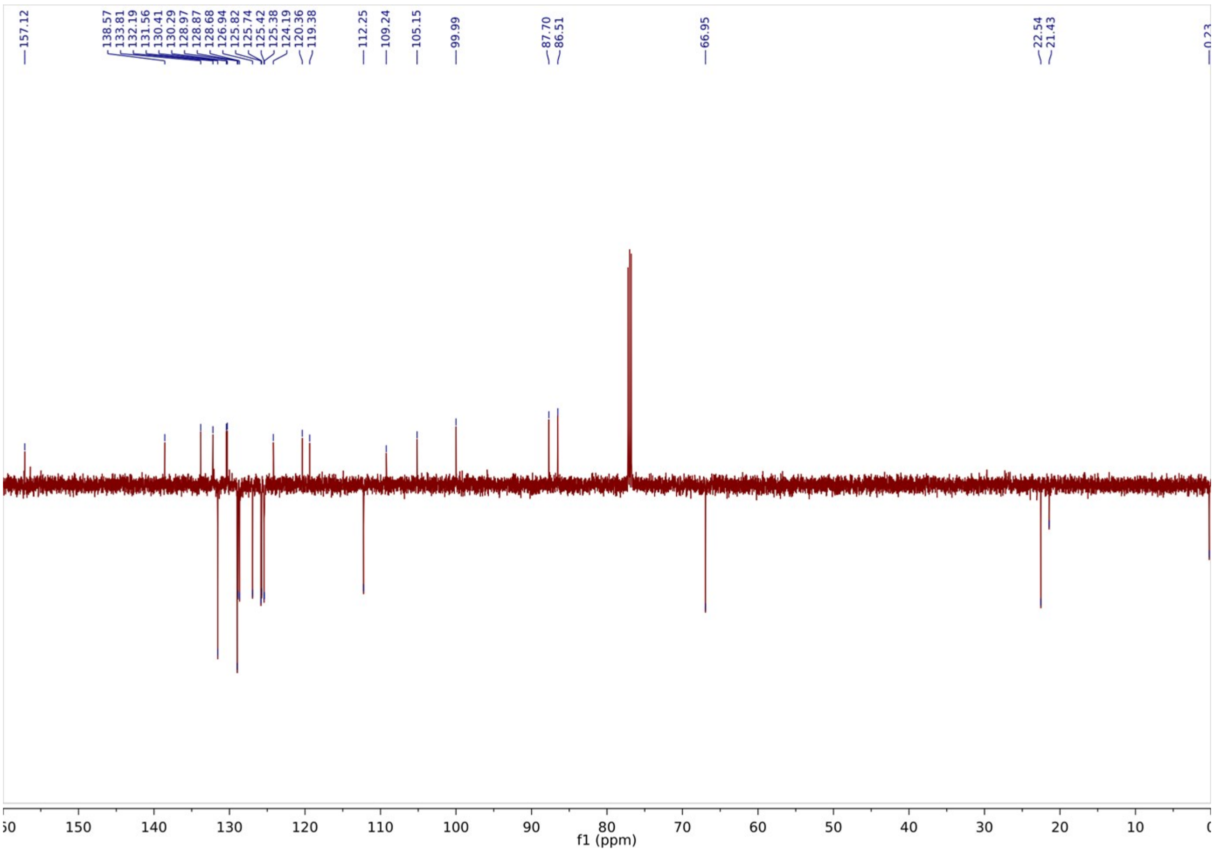
f1 (ppm)

## References


1. Geny, A.; Agenet, N.; Iannazzo, L.; Malacria, M.; Aubert, C.; Gandon, V. Air-Stable {(C5h5)Co} Catalysts for [2+2+2] Cycloadditions. *Angew. Chem. Int. Ed.* **2009,** *48*, 1810-1813.
2. Alexandrová, Z.; Stará, I. G.; Sehnal, P.; Teplý, F.; Starý, I.; Šaman, D.; Fiedler, P. Synthetic Studies toward Chiral Aromatic Triynes as Key Substrates for the Asymmetric Synthesis of Helicene-Like Molecules. *Collect. Czech. Chem. Commun.* **2004,** *69*, 2193-2211.
3. Coventry, D. N.; Batsanov, A. S.; Goeta, A. E.; Howard, J. A. K.; Marder, T. B.; Perutz, R. N. Selective Ir-Catalysed Borylation of Polycyclic Aromatic Hydrocarbons: Structures of Naphthalene-2,6-Bis(Boronate), Pyrene-2,7-Bis(Boronate) and Perylene-2,5,8,11-Tetra(Boronate) Esters. *Chem. Commun.* **2005,** 2172-2174.
4. Buchta, M.; Rybáček, J.; Jančařík, A.; Kudale, A. A.; Buděšínský, M.; Chocholoušová, J. V.; Vacek, J.; Bednárova, L.; Císařová, I.; Bodwell, G. J.; Starý, I.; Stará, I. G. Chimerical Pyrene-Based [7]Helicenes as Twisted Polycondensed Aromatics. *Chem. Eur. J.* **2015,** *21*, 8910-8917.
5. Žádný, J.; Jančařík, A.; Andronova, A.; Šámal, M.; Chocholoušová, J. V.; Vacek, J.; Pohl, R.; Šaman, D.; Císařová, I.; Stará, I. G.; Starý, I. A General Approach to Optically Pure [5]-, [6]-, and [7]Heterohelicenes. *Angew. Chem. Int. Ed.* **2012,** *51*, 5857-5861.
6. Šámal, M.; Chercheja, S.; Rybáček, J.; Chocholoušová, J. V.; Vacek, J.; Bednárova, L.; Šaman, D.; Stará, I. G.; Starý, I. An Ultimate Stereocontrol in Asymmetric Synthesis of Optically Pure Fully Aromatic Helicenes. *J. Am. Chem. Soc.* **2015,** *137*, 8469-8474.
7. Nejedlý, J.; Šámal, M.; Rybáček, J.; Tobrmanová, M.; Szydlo, F.; Coudret, C.; Neumeier, M.; Vacek, J.; Chocholoušova, J. V.; Buděšínský, M.; Šaman, D.; Bednárova, L.; Sieger, L.; Stará, I. G.; Starý, I. Synthesis of Long Oxahelicenes by Polycyclization in a Flow Reactor. *Angew. Chem. Int. Ed.* **2017,** *56*, 5839-5843.
8. Crawford, A. G.; Liu, Z. Q.; Mkhalid, I. A. I.; Thibault, M. H.; Schwarz, N.; Alcaraz, G.; Steffen, A.; Collings, J. C.; Batsanov, A. S.; Howard, J. A. K.; Marder, T. B. Synthesis of 2-and 2,7-Functionalized Pyrene Derivatives: An Application of Selective C-H Borylation. *Chem. Eur. J.* **2012,** *18*, 5022-5035.
9. Kolehmainen, E.; Laihia, K.; Laatikainen, R.; Vepsalainen, J.; Niemitz, M.; Suontamo, R. Complete Spectral Analysis of the H-1 Nmr 16-Spin System of Beta-Pinene. *Magn. Reson. Chem.* **1997,** *35*, 463-467.
10. Frisch, M. J.; Trucks, G. W.; Schlegel, H. B.; Scuseria, G. E.; Robb, M. A.; Cheeseman, J. R.; Scalmani, G.; Barone, V.; Mennucci, B.; Petersson, G. A.; Nakatsuji, H.; Caricato, X.; Li, X.; Hratchian, H. P.; Izmaylov, A. F.; Bloino, J.; Zheng, G.; Sonnenberg, J. L.; Hada, M.; Ehara, M.; Toyota, K.; Fukuda, R.; Hasegawa, J.; Ishida, M.; Nakajima, T.; Honda, Y.; Kitao, O.; Nakai, H.; Vreven, T.; Montgomery, J., J. A.; Peralta, J. E.; Ogliaro, F.; Bearpark, M.; Heyd, J. J.; Brothers, E.; Kudin, K. N.; Staroverov, V. N.; Kobayashi, R.; Normand, J.; Raghavachari, K.; Rendell, A.; Burant, J. C.; Iyengar, S. S.; Tomasi, J.; Cossi, M.; Rega, N.; Millam, J. M.; Klene, M.; Knox, J. E.; Cross, J. B.; Bakken, V.; Adamo, C.; Jaramillo, J.; Gomperts, R.; Stratmann, R. E.; Yazyev, O.; Austin, A. J.; Cammi, R.; Pomelli, C.; Ochterski, J. W.; Martin, R. L.; Morokuma, K.; Zakrzewski, V. G.; Voth, G. A.; Salvador, P.; Dannenberg, J. J.; Dapprich, S.; Daniels, A. D.; Farkas, O.; Foresman, J. B.; Ortiz, J. V.; Cioslowski, J.; Fox, D. J. *Gaussian 09, Revision A.02*, Gaussian, Inc.: Wallingford CT, 2009.
11. Becke, A. D. Density-Functional Thermochemistry 3. The Role of Exact Exchange. *J. Chem. Phys.* **1993,** *98*, 5648-5652.
12. Lee, C. T.; Yang, W. T.; Parr, R. G. Development of the Colle-Salvetti Correlation-Energy Formula into a Functional of the Electron-Density. *Phys. Rev. B* **1988,** *37*, 785-789.
13. Auer, A. A.; Gauss, J. Triple Excitation Effects in Coupled-Cluster Calculations of Indirect Spin-Spin Coupling Constants. *J. Chem. Phys.* **2001,** *115*, 1619-1622.
14. CFOUR, Coupled-Cluster techniques for Computational Chemistry, a quantum-chemical program package by J.F. Stanton, J. Gauss, L. Cheng, M.E. Harding, D.A. Matthews, P.G. Szalay with contributions from A.A. Auer, R.J. Bartlett, U. Benedikt, C. Berger, D.E. Bernholdt, Y.J. Bomble, O. Christiansen, F. Engel, R. Faber, M. Heckert, O. Heun, M. Hilgenberg, C. Huber, T.-C. Jagau, D. Jonsson, J. Jusélius, T. Kirsch, K. Klein, W.J. Lauderdale, F. Lipparini, T. Metzroth, L.A. Mück, D.P.

O'Neill, D.R. Price, E. Prochnow, C. Puzzarini, K. Ruud, F. Schiffmann, W. Schwalbach, C. Simmons, S. Stopkowicz, A. Tajti, J. Vázquez, F. Wang, J.D. Watts and the integral packages MOLECULE (J. Almlöf and P.R. Taylor), PROPS (P.R. Taylor), ABACUS (T. Helgaker, H.J. Aa. Jensen, P. Jørgensen, and J. Olsen), and ECP routines by A. V. Mitin and C. van Wüllen. For the current version, see http://www.cfour.de.
15. Salahub, D. R.; Fournier, R.; Mlynarski, P.; Papai, I.; St-Amant, A.; Ushio, J., Gaussian-Based Density Functional Methodology, Software, and Applications. In *Density Functional Methods in Chemistry*, Labanowski, J. K.; Andzelm, J. W., Eds. Springer: Berlin, 1991; pp 77; Modified version: Malkin V.G., Malkina O.L., deMon-NMR program, Bratislava, version 2016.
16. Perdew, J. P.; Yue, W. Accurate and Simple Density Functional for the Electronic Exchange Energy - Generalized Gradient Approximation. *Phys. Rev. B* **1986,** *33*, 8800-8802.
17. Perdew, J. P. Correction. *Phys. Rev. B* **1989,** *40*, 3399-3399.
18. Perdew, J. P. Density-Functional Approximation for the Correlation-Energy of the Inhomogeneous Electron-Gas. *Phys. Rev. B* **1986,** *33*, 8822-8824.
19. Perdew, J. P. Correction. *Phys. Rev. B* **1986,** *34*, 7406-7406.
20. Kutzelnigg, W.; Fleischer, U.; Schindler, M., *Al - Cl: The Iglo-Method: Ab Initio Calculation and Interpretation of Nmr Chemical Shifts and Magnetic Susceptibilities*. Springer-Verlag: Heidelberg, 1990; Vol. 23.
21. Hanwell, M. D.; Curtis, D. E.; Lonie, D. C.; Vandermeersch, T.; Zurek, E.; Hutchison, G. R. Avogadro: An Advanced Semantic Chemical Editor, Visualization, and Analysis Platform. *J. Cheminformatics* **2012,** *4*, 17.
22. Pipek, J.; Mezey, P. G. A Fast Intrinsic Localization Procedure Applicable for Abinitio and Semiempirical Linear Combination of Atomic Orbital Wave-Functions. *J. Chem. Phys.* **1989,** *90*, 4916-4926.
23. Malkina, O. L.; Křístková, A.; Malkin, E.; Komorovský, S.; Malkin, V. G. Illumination of the Effect of the Overlap of Lone-Pairs on Indirect Nuclear Spin-Spin Coupling Constants. *Phys. Chem. Chem. Phys.* **2011,** *13*, 16015-16021.
24. Bader, R. F. W. A Quantum-Theory of Molecular-Structure and Its Applications. *Chem. Rev.* **1991,** *91*, 893-928.
25. Aimall (Version 17.11.14), Todd A. Keith, Tk Gristmill Software, Overland Park Ks, USA, 2017 (Aim.Tkgristmill.Com).
26. Grimme, S. Semiempirical Gga-Type Density Functional Constructed with a Long-Range Dispersion Correction. *J. Comput. Chem.* **2006,** *27*, 1787-1799.
27. Grimme, S.; Antony, J.; Ehrlich, S.; Krieg, H. A Consistent and Accurate Ab Initio Parametrization of Density Functional Dispersion Correction (Dft-D) for the 94 Elements H-Pu. *J. Chem. Phys.* **2010,** *132*, 154104.
